# A Plant Virus Movement Protein Regulates the Gcn2p Kinase in Budding Yeast

**DOI:** 10.1371/journal.pone.0027409

**Published:** 2011-11-08

**Authors:** Frederic Aparicio, Rafael Aparicio-Sanchis, José Gadea, Jesús Ángel Sánchez-Navarro, Vicente Pallás, José Ramón Murguía

**Affiliations:** Department of Stress Biology, Instituto de Biología Molecular y Celular de Plantas (UPV-CSIC), Valencia, Spain; Ecole Normale Superieure, France

## Abstract

Virus life cycle heavily depends on their ability to command the host machinery in order to translate their genomes. Animal viruses have been shown to interfere with host translation machinery by expressing viral proteins that either maintain or inhibit eIF2α function by phosphorylation. However, this interference mechanism has not been described for any plant virus yet. *Prunnus necrotic ringspot virus* (PNRSV) is a serious pathogen of cultivated stone fruit trees. The movement protein (MP) of PNRSV is necessary for the cell-to-cell movement of the virus. By using a yeast-based approach we have found that over-expression of the PNRSV MP caused a severe growth defect in yeast cells. cDNA microarrays analysis carried out to characterise at the molecular level the growth interference phenotype reported the induction of genes related to amino acid deprivation suggesting that expression of MP activates the GCN pathway in yeast cells. Accordingly, PNRSV MP triggered activation of the Gcn2p kinase, as judged by increased eIF2α phosphorylation. Activation of Gcn2p by MP expression required a functional Tor1p kinase, since rapamycin treatment alleviated the yeast cell growth defect and blocked eIF2α phosphorylation triggered by MP expression. Overall, these findings uncover a previously uncharacterised function for PNRSV MP viral protein, and point out at Tor1p and Gcn2p kinases as candidate susceptibility factors for plant viral infections.

## Introduction

*Saccharomyces cerevisiae* cells transiently inhibit initiation of protein synthesis under environmental stress to avoid misfolding of proteins which could compromise cell viability. Inhibition of translation is achieved by phosphorylation of the alpha subunit of the eukaryotic translation initiation factor-2 (eIF2α). The sole eIF2α kinase present in budding yeast is encoded by the *GCN2* gene. The general control non- repressible 2 protein (Gcn2p) regulates the selective translation of the Gcn4p transcription factor upon nutrient deprivation conditions. This regulation is exerted by four uORFs located in the 5′ untranslated region of the *GCN4* mRNA, which makes it hypersensitive to eIF2 levels. When there is no amino acid limitation, the levels of active eIF2 are high and the uORFs block translation of *GCN4* mRNA. In cells under amino acid starvation there is an accumulation of uncharged tRNAs that stimulates Gcn2p kinase activity. Phosphorylation of eIF2α diminishes the levels of active eIF2 and alleviates inhibition by uORFs, thus favouring *GCN4* mRNA translation as well as blocking general translation [1]. High levels of Gcn4p then activate the expression of genes encoding amino acid biosynthesis pathways. Increased eIF2α phosphorylation also occurs by starvation for purines, glucose, growth on ethanol, high salinity in the growth medium and treatment with methyl methanesulfonate [2], [3]. Thus, Gcn2p seems to be a master regulator of gene expression in yeast and in response to various kinds of stresses. The fundamental nature of the GCN pathway is reflected by the fact that Gcn2p function is conserved throughout evolution. In mammals four protein kinases are known to phosphorylate eIF2α, namely GCN2, PKR, HRI and PERK. In plants, *Arabidopsis* and rice genome analysis revealed that plants apparently encode a single eIF2α kinase orthologue of yeast GCN2 [4], [5].

Virus life cycle heavily depends on their ability to command the host machinery in order to translate their genomes. This implies that viral RNAs must compete with cellular mRNAs for the host translation apparatus. To achieve this, viruses have devised strategies to inhibit cellular protein synthesis while ensuring efficient translation of their cognate RNAs. Indeed, different viral proteins have been shown to mimic host nutrient/growth signals which activate signaling pathways that ensure viral replication. For example, adenoviruses have evolved proteins that activate the mTOR pathway, irrespective of the cellular microenvironment [6]. mTOR integrates nutrient and growth factor signals to regulate the translation initiation of mRNAs important for cell growth [7]. Another strategy consists of regulating eIF2 function by phosphorylation/dephosphorylation. Thus, the g34.5 protein from HSV-1 complexes with the cellular protein phosphatase 1a (PP1a) and directs dephosphorylation of eIF2α [8]. Vaccinia virus K3L polypeptide reduces the level of eIF2α phosphorylation by GCN2 [9]. Rotaviruses cause eIF2α phosphorylation depending on the synthesis of three viral proteins, VP2, NSP2, and NSP5 [10], [11]. In Arabidopsis, phosphorylation of eIF2α by GCN2 kinase occurs in response to environmental stress such as amino acid starvation, cold shock, and wounding [12], [13]. However, increase in eIF2α phosphorylation by plant virus infection remains controversial. On one hand, *Tobacco mosaic virus* (TMV) infection in plants silenced for p58IPK, the plant ortholog of the mammalian double-stranded RNA-dependent protein kinase PKR inhibitor leads to host death. This host cell death is associated with phosphorylation of eIF2α [14]. In addition, expression of the non phosphorylatable eIF2α (S51A) mutant rescues silenced plants from death induced by viruses [14]. On the other, AteIF2α is not phosphorylated in plants infected with *Turnip yellow mosaic virus* (TuMV) or *Turnip crinkle virus* (TCV) suggesting that phosphorylation of this factor is not a plant response to infection of these two viruses [13].

To establish a systemic infection, viruses invade neighboring cells via cell to cell movement trough plasmodesmata channels until they reach the vascular system [15], [16]. This cell to cell movement is an active process involving one o more movement proteins (MP) encoded by the virus which have to interact with other viral and host factors [17]. In agreement with its essential role in cell to cell movement, diverse host proteins involved in the regulation of the exclusion limit of plasmosdesmata, vesicle trafficking and association to the cytoskeleton have been found to interact to several MPs of the 30K superfamily, a group of 18 different genera which MPs are related to the 30 kD MP of *Tobacco mosaic virus* (TMV) [18]. Interactions with other host factors as regulating enzymes and members of the heat stress family, which in some cases influence viral movement, have been also reported [19]–[25]. All together, these published data point to a scenario where the MPs would be multifunctional proteins acting at different levels. Interestingly, virus interference trough their MPs of the host transport process would represent a potentially effective means of disrupting host physiology [26].

*Prunus necrotic ringspot rvirus* (PNRSV) is a serious pathogen of cultivated stone fruit trees [27] that belongs to the Ilarvirus genus [28], [29]. PNRSV MP (thereafter MPpnrsv) is classified into the 30K superfamily [18] and contains a hydrophobic region preceding to a RNA-binding domain, both necessary for the cell-to-cell movement of the virus [30]–[34]. Budding yeast has been largely recognized as a model host to shed light on fundamental processes of plant virus replication, pathology and evolution. The invaluable advantages of using this versatile eukaryotic system to study plant virus interactions have been reviewed in detail [35], [36].

In the present work we implemented a genetic strategy for the identification of yeast factors/pathways affected by the MPpnrsv. We found that the over-expression of the MPpnrsv in budding yeast induced a severe growth defect in yeast cells. A similar growth defect was also observed when expressing other MPs of the 30K family. Transcriptional profiling revealed that MPpnrsv triggered the expression of amino acid biosynthesis genes, and incremented eIF2α phosphorylation. Furthermore, deletion of *GCN2* and *GCN4* genes alleviated the slow growth phenotype associated with MPpnrsv expression. Most interestingly, Gcn2p activation seemed to be dependent on the nutrient regulated kinase TOR1 since rapamycin treatment alleviated both growth defects and eIF2 phosphorylation in a dose-dependent manner. Therefore, MPpnrsv triggers several nutrient-regulated pathways which would i) uncover a new function for this viral protein and ii) suggest Tor1p and Gcn2p kinases as candidate susceptibility factors for plant viral infections.

## Results

### Overexpression of PNRSV MP negatively affects growth in yeast

**Figure 1:**
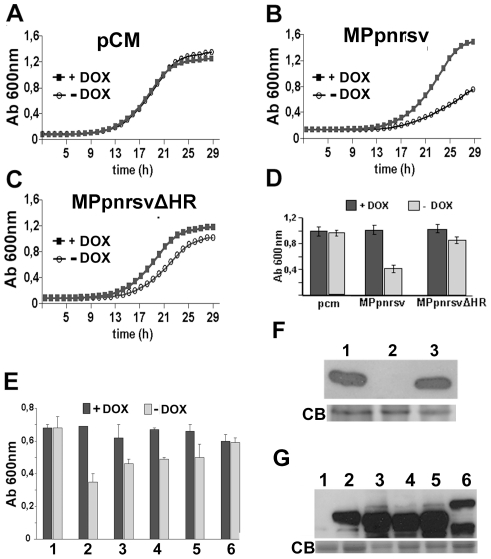
Expression of viral MPs of the 30 K superfamily interferes with yeast cell growth. Growth kinetics of WT yeast cells cultured in SD medium +/- Doxycycline (DOX) and transformed with empty vector (pCM) (A), a full-length MPpnrsv expressing plasmid (B) or a truncated MPpnrsvΔHR expressing plasmid (C). (D) Quantitation of cell growth in SD medium+/- DOX of yeast cultures transformed with vector alone or with expressing MPpnrsv and MPpnrsvΔH plasmids. (E) Quantitation of cell growth in SD medium+/- DOX, of WT yeasts transformed with either pCM (lane 1) or with plasmids expressing PNRSV, BMV, CMV, TMV and GLFV MPs (lanes 2, 3, 4, 5 and 6, respectively). Data in D and E, represent the average +/- standard error (s.e.) of at least three independent experiments, each one done in triplicate (p<0.001). (F) Immunodetection of MPpnrsv (lane 3) and MPpnrsvΔHR (lane 1) in protein extracts from yeast cells transformed with the corresponding expression vectors or pCM as negative control (lane 2). (G) The same as in (F), but in protein extracts from yeast cells transformed with pCM (lane 1) or plasmids expressing PNRSV, BMV, CMV, TMV or GLFV MPs (lanes 2, 3, 4, 5 and 6, respectively) Coomassie Blue (CB) stained gel used as loading control).

The PNRSV MP (MPpnrsv) protein is 282 amino acids long, and contains the characteristic hydrophobic region preceded by an RNA-binding domain. Both regions are present in all members of the ilarvirus genus and are necessary for the cell-to-cell viral transport [28], [33], [34]. We used a yeast-based approach to decipher how MPpnrsv affects and/or modulates host factors. HA-tagged versions of MPpnrsv and MPpnrsvΔHR, a non-functional MPpnrsv mutant lacking its hydrophobic region [34], were over-expressed in wild type (WT) yeast cells under the control of a yeast plasmid with a doxycycline-repressible promoter, and yeast cell growth was monitored in the absence/presence of doxycycline. As expected, doxycycline treatment did not affect yeast cell growth (Fig. 1A). Interestingly, MPpnrsv-expressing yeast cells exhibited a severe growth defect in a doxycycline-dependent manner (Fig. 1B). Expression of MPpnrsvΔHR also induced a slight growth defect, but not to such extent as MPpnrsv (Figs. 1C, D). Similar growth defects have been reported in yeasts expressing foreign proteins [37]. It is possible that a similar scenario occurs when expressing MPpnrsvΔHR in yeast, although we cannot rule out that other mechanisms may be involved. Differences in growth were not due to expression levels as both MPpnrsv and MPpnrsvΔHR proteins accumulated similarly (Fig. 1F). Therefore, the strong growth interference phenotype required a functional MPpnrsv. To further analyze the specific MPpnrsv-induced growth interference phenotype, we explored the effect on yeast cell growth of other MPs from the 30 K superfamily. Those included MPs from two phylogenetically related viruses as *Brome mosaic virus* (BMV) and *Cucumber mosaic virus* (CMV) and two non-related viruses as *Tobacco mosaic virus* (TMV) and *Grapevine fanleaf virus* (GFLV) respectively. HA-tagged versions of these MPs were also expressed in yeast under the control of the yeast plasmid doxycycline-repressible promoter. Measurement of the yeast cell growth at the exponential phase with or without doxycycline showed that BMV, CMV and TMV MPs expression elicited a growth interference phenotype that was totally absent in the case of GFLV MP (Fig. 1E). Western blot analysis confirmed that all MPs accumulated at similar levels in yeast cells (Fig. 1G). Interestingly, all the MPs analyzed, except the GFLV MP, are associated to the membrane of the endoplasmic reticulum (ER) suggesting that the growth defect observed in yeast was somehow linked to MPs that move trough an ER associated pathway.

### MPpnrsv overexpression triggers growth defect in yeast cells by activating the GCN pathway

**Figure 2:**
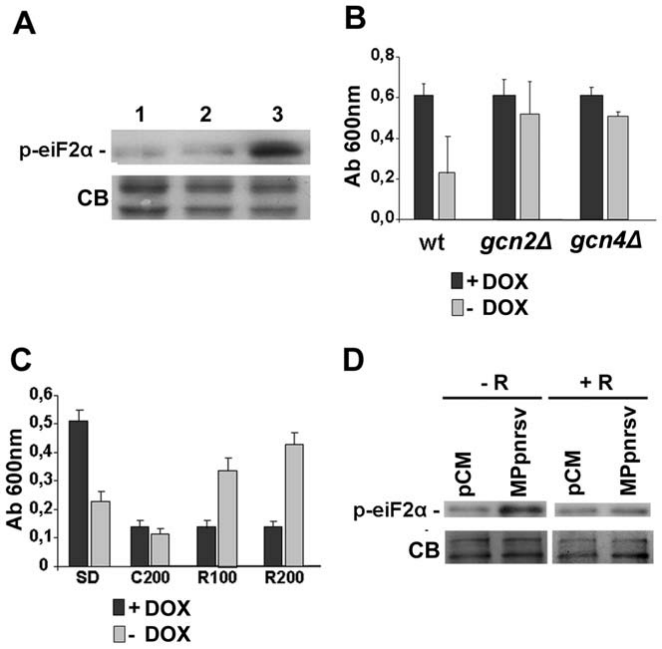
MPpnrsv expression activates the GCN pathway in a TOR pathway-dependent manner. (A) Immunodetection of phospho- eIF-2α levels in protein extracts from yeast cells transformed with pCM (lane 1), MPpnrsvΔHR (lane 2) and MPpnrsv (lane 3) expressing plasmids. Yeast cells were growth for 3 hours in SD medium without DOX. Even loading of gels was confirmed by CB staining of membranes after transfer. (B) Disruption of the GCN pathway alleviates the growth defect caused by MPpnrsv expression in yeast. Quantitation of cell growth in SD medium+/- DOX, of *WT*, *gcn2Δ* and *gcn4Δ* yeast strains expressing the MPpnrsv. (C) Rapamycin treatment alleviates the growth defect caused by MPpnrsv expression in yeast. Yeast cells expressing MPpnrsv were grown in SD medium +/- DOX (SD), the same medium with 200 µg/ml cycloheximide (C200), and with 100 µg/ml (R100) or 200 µg/ml (R200) Rapamycin respectively. Data in B and C represent the average +/- s.e. of at least three independent experiments, each one done in triplicate. (p<0.005) (D) Rapamycin treatment blocks phosphorylation of yeast eIF-2α triggered by MPpnrsv expression. Immunodetection of phospho- eIF-2α levels in protein extracts from yeast cells transformed with pCM and MPpnrsv expressing plasmids, grown in SD medium with (+R) or without (-R) 200 µg/ml Rapamycin for three hours. Coomassie Blue (CB) stained gel used as loading control.

To further characterize at the molecular level the growth interference phenotype induced by MPpnrsv expression, we compared the gene expression profile of the MPpnrsv-expressing strain with that of the wild type (WT) strain using cDNA microarrays. We used Significance Analysis of Microarrays to identify differentially expressed genes between the WT and MPpnrsv strains. Any given gene was considered to be differentially regulated when its expression was at least 2 fold higher or lower in the MPpnrsv strain than in the WT strain (FDR<6%). Following these criteria, 324 genes were induced by MPpnrsv over-expression. Surprisingly, no gene was found to be differentially repressed. We performed a functional classification of induced genes according to Gene Ontology (GO) terms using the GO Term Finder tool. Interestingly, functional categories such as glutamine and nitrogen metabolism were significantly enriched in the set of induced genes (see Table S1). Nutrient deprivation activates glutamine and nitrogen metabolism gene expression in budding yeast. Nutrient deprivation conditions trigger the GCN pathway, a nutrient-sensing pathway that transiently inhibits translation initiation and simultaneously favors selective translation of the GCN4 mRNA. GCN4 encodes a transcriptional activator of amino acid biosynthesis genes. The major transducer of the GCN pathway is the Gcn2p kinase which phosphorylates eIF2α. To explore whether over expression of MPpnrsv was able to activate the GCN pathway we measured the phosphorylation status of eIF2α in WT yeast cells by immunoblot analysis using a commercially available polyclonal antibody that specifically recognizes eIF2α phosphorylated at serine 51 (see methods). As shown in Figure 2A, overexpression of MPpnrsv, but not the mutant MPpnrsvΔHR, incremented eIF2α phosphorylation in WT yeast cells, thus showing that MPpnrsv expression activated the Gcn2p kinase. Furthermore, the growth interference phenotype associated with MPpnrsv overexpression was alleviated in *gcn2Δ* or *gcn4Δ* yeast genetic background (Fig. 2B). Given that eIF2α phosphorylation was not reversed in the *gcn4Δ* mutant (see Fig. S1), these data indicated that MPpnrsv expression triggered a growth defect in yeast cells likely by activating the GCN pathway.

To gain further insights into the mechanism by which MPpnrsv activated the GCN pathway, we performed a microarray experiment to identify differentially expressed genes between the MPpnrsv and the mutant MPpnrsvΔHR overexpressing strains. Data analysis using the GO Term Finder tool revealed that functional categories such as ribosome biogenesis and initiation of protein synthesis were enriched in the set of differentially expressed genes (see Table S2 and S3). These functional categories are mainly regulated by the TOR1 nutrient sensing kinase. Therefore, we explored the involvement of TOR1 in MPpnrsv-dependent yeast growth defect using rapamycin, a highly selective inhibitor of the Torp1 kinase. Yeast cells expressing MPpnrsv were grown in minimal medium with or without doxycycline and increasing doses of rapamycin. As shown in Figure 2C, treatment with rapamycin alleviated the yeast cell growth defect induced by MPpnrsv expression in a dose dependent manner. Rapamycin effect was specific as cycloheximide treatment blocked yeast growth regardless the presence of MPpnrsv. Most remarkably, rapamycin treatment blocked eIF2α phosphorylation triggered by MPpnrsv expression (Fig. 2D). Hence, activation of Gcn2p by MPpnrsv expression required a functional Tor1p kinase, thus uncovering a functional link between both nutrient-sensing pathways.

## Discussion

By using a yeast-based approach we have identified that the PNRSV MP is able to specifically activate the nutrient regulated kinase Gcn2p. Gcn2p activation seemed to be dependent on the nutrient regulated kinase Tor1p, as rapamycin treatment alleviated the growth defects and eIF2α phosphorylation in a dose-dependent manner. Therefore, the data here suggest that MPpnrsv has the ability to activate Gcn2p and Tor1p nutrient-regulated pathways. These findings uncover a previously uncharacterized function of MPpnrsv, and highlight Tor1p and Gcn2p kinases as candidate susceptibility factors for plant viral infections.

It is widely accepted that the main function of MPs is to assist virus in cell-to-cell movement across plant tissues [17]. In addition, plant virus MPs have been shown to be pathogenicity determinants in some virus-host interactions and in some instances cause developmental and metabolic alterations [26]. The increasing number of host virus factors interacting with viral MPs [20], [21] indicate their relevant role in the viral infection process. The demonstration that the MPpnrsv is able to increase eIF2α phosphorylation in a Tor1p–dependent manner might help to uncover an intriguing function of plant virus MP in this relevant cellular route. Our data indicate that MPpnrsv expression increases eIF2α phosphoryaltion by activating the Gcn2p kinase (Figure 2A, B). How Gcn2p is activated by MPpnrsv remains to be determined. Interestingly, the hydrophobic region (HR) of the protein seems to be required for Gcn2p activation. It is worth noting that the HR of the PNRSV MP has been shown to be essential for a complete viral life cycle [34]. Gcn2p activation was shown to be dependent on the nutrient-regulated kinase Tor1p. Tor1p integrates nutrient and growth factor signals to regulate the translation initiation of mRNAs important for cell growth and proliferation. Although still controversial, Tor1p seems to favour selective translation of TOP mRNAs (a class of mRNAs bearing 5-terminal oligopyrimidine tracts which encode components of the translational machinery) by signaling through ribosomal protein S6 (rpS6) kinase (S6K1) to rpS6. [38], [39]. As genomic PNRSV RNAs could be considered as TOP-like RNAs [30], [40], it is tempting to speculate that MPpnrsv could enhance selective viral mRNA expression and viral replication by activating the Tor1p pathway, similarly as was recently described for adenoviral E4-ORF1 and E4-ORF4 proteins [6]. Along the same line, the plant viral reinitiation factor transactivator–viroplasmin (TAV) has been shown to recruit the TOR/S6K1 signaling complex, with TAV-TOR binding being critical for both translation re-initiation and viral fitness [41].

The functional linkage that connects Tor1p with Gcn2p activation by MPpnrsv expression remains unanswered. Recent studies in yeast have found that the GCN pathway is a major effector of the TOR pathway [42]. Under nutrient stress, Gcn2p was induced by the release of TOR repression through a mechanism involving Sit4p protein phosphatase. This mechanism might well be triggered by expression of MPpnrsv in yeast. Indeed, the functional categories enriched in transcriptome of yeast expressing MPpnrsv resembled those of yeast cells subjected to nutrient stress [42]. Alternatively, constitutive hyper stimulation of Tor1p by MPpnrsv expression regardless the nutrient status, would favor precocious cell entry into S-phase thus leading to replicative stress. It is well established that Gcn2p negatively regulates cell cycle progression in response to replicative stress [43], [44]. The growth interference phenotype elicited by MPpnrsv, could then be interpreted as a consequence of the cell cycle delay triggered by Gcn2p activation. Indeed, exponential yeast cultures overexpressing MPpnrsv exhibited an increased number of unbudded cells, thus reflecting a delay at the G1/S transition (Table S4). In addition, the fact that rapamycin treatment relieved the MPpnrsv-dependent growth defect in yeast is also consistent with this hypothesis. We cannot discard as well, that the activation of the two opposite pathways, Tor1p and Gcn2p, could be considered a compensative effect derived from the cell location of the viral MPs. It is well know that all eukaryotes respond to ER stress through a set of pathways known as the unfolded protein response (UPR) [45], [46] which include the eIF2α phosphorylation. The up-regulation of the Tor1p pathway could be response to mitigate the UPR triggered by the viral MPs that are associated to the membrane of ER, avoiding the transient decrease in global translation. In this sense, we do not expect a growth interference phenotype using viral MP that are not located to the ER as we observed with the MPpnrsvΔHR or the MP of GFLV.

Taken together, our results point out at Tor1p and Gcn2p kinases as candidate susceptibility host factors during plant viral infections. Considering that both kinases are conserved throughout evolution, experimental validation of these candidate factors in a plant model of viral infection would further support this possibility, as recently described for other viral factors[41]. To test this hypothesis, we are currently analyzing the phosphorylation status of Gcn2p/eIF2α and TOR/S6K1 signaling proteins in transgenic *A. thaliana* plants overexpressing MPpnrsv. Successful completion of these experiments would definitively open a new research area on viral MPs functions and plant-virus interactions.

## Methods

### Strains, plasmids, media and general methods

Specific primers were used to amplify the MP of PNRSV, mutant lacking its hydrophobic domain (MPpnrsvΔHR) and MPs of BMV, CMV,TMV and GFLV (see Table S5). PCR products were inserted between *Pme* I-*Pst* I sites of the centromeric plasmid pCM265 to express the corresponding proteins in yeast cells. In all these constructs, MP ORF is in frame with three copies of the HA plus a histidine epitope. Plasmids were confirmed by DNA sequencing. All yeast strains used in this study are listed in Table S6. Standard methods for yeast culture and manipulations were used (minimal medium (SD) contained 2% glucose, 0.67% yeast nitrogen base and the appropriate amino acid supplements to maintain selection of URA3 and cultures were routinely grown at 30°C). In growth curve analyses, cells were grown in microtiter plates using the Bioscreen C system (Thermo) with or without Doxycycline. For cycloheximide and rapamycin experiments, cells were grown in presence of 100 and 200 µg/ml of each drug, respectively.

### Immunoblotting analysis

Yeast strains were grown in minimal medium without Doxycycline. At the indicated times cells were centrifuged. Cell pellets were then resuspended in 5X Laemmli buffer and boiled for 10 min. Total cellular protein was subjected to SDS Polyacrylamide Gel Electrophoresis (SDS-PAGE) and transferred to PVDF membranes (Amersham). Viral proteins were detected with anti HA-antibody (ROCHE) whereas a specific phospho-eIF2a (Ser51) antibody (Cell Signalling) was used to analyze the phosphorylation state of eIF2α. In all cases, western-blots were carried out following manufacturer's recommendations. Immunocomplexes were visualized by enhanced chemiluminescence detection (ECL Amersham) using anti-Rabbit IgG HRP conjugated (Amersham). Uniform gel loading was confirmed by Coomassie blue staining of membranes after immunoblot studies.

### Microarray assay

Yeast cell cultures were grown 24 h in minimal medium with doxycycline (100 mg/ml). These cultures were then pelleted and diluted to an OD600 nm of 0.1 into fresh minimal medium without doxycycline. At 6 h total RNA was extracted from yeast cells transformed with the empty pCM262 vector (pCM construct), pCM:MPpnrsv and pCM:MPpnrsvΔHR. RNA samples were amplified using Message Amp II amplification kit (Ambion). 7.5 µg of UTP-aminoallyl-amplified RNA (aRNA) were labelled using Cy3or Cy5 dye (GE Healthcare), purified using Megaclear columns (Ambion), and quantified using Nanodrop spectrophotometer. 150 pmol of labeled-aRNA were dried and resuspended in hybridization buffer containing 3×SSC, 0.1% SDS, 0.1% salmon sperm DNA and 50% formamide. Microarray hybridization was performed manually using Telechem Hybridization Chambers, following Corning instructions. Slides were scanned using a GenePix 4000B scanner and analyzed using GenePix 6.0 software (Molecular Devices). Data were normalized by mean global intensity and Lowess correction. Spots with a net intensity in both channels lower than twice the median signal background were removed as low-signal spots, and were not considered for further analysis. Significance analysis of microarrays (SAM) [47] was performed on the pre-processed and normalized data sets to identify differentially expressed genes. A 5% false discovery rate (FDR) and 2-fold expression cut off was considered to determine up-regulated and down-regulated genes. A functional category analysis of the up-regulated or down-regulated genes was carried out using The GO Term Finder utility (www.http://go.princeton.edu/cgi-bin/GOTermFinder). Enriched categories with *P* values smaller than 0.05 were further considered. The raw data discussed in this publication is MIAME compliant has been deposited in NCBIs Gene Expression Omnibus (GEO, http://www.ncbi.nlm.nih.gov/geo/) and are accessible through the GEO Series accession numbers GSE26119 and GSE26120, respectively.

## Supporting Information

Figure S1 **Analysis of eIF-2α phosphorylation in yeast** ***gcn2Δ and gcn4Δ*** **mutants**. Immunodetection of phospho-eIF2α (p-eIF2α) and MPpnrsv (MPp) levels in protein extracts from wt, *gcn4Δ* and *gcn2Δ* yeast strains expressing MPpnrsv. Yeast cells were grown for3 hours in SD medium without DOX. MPpnrsv and phosphorylated eIF2α were detected as described in materials and methods. Coomassie Blue (CB) stained gel used as loading control.
(TIF)

Table S1 **Functional classification of induced genes in MPpnrsv versus the empty vector expressing yeast strains.**
(DOC)

Table S2 **Functional classification of induced genes in MPpnrsv versus MPpnrsvΔHR expressing yeast strains.**
(DOC)

Table S3 **Functional classification of repressed genes in MPpnrsv versus MPpnrsvΔHR expressing yeast strains.**
(DOC)

Table S4 **Percentage of unbudded cells in yeasts transformed with the empty vector (pCM) and MPpnrsv (MP) expressing plasmids.**
(DOC)

Table S5 **Primers used in this study. Restriction sites** ***Pme*** **I** ***and Pst*** **I are underlined.**
(DOC)

Table S6 **Yeast Strains used in this study.**
(DOC)
