# Supplementary figures and images for: A Plant Virus Movement Protein Regulates the Gcn2p Kinase in Budding Yeast
Source: PLoS One. 2011 Nov 8;6(11):e27409. doi: 10.1371/journal.pone.0027409 (PMC3210792; doi:10.1371/journal.pone.0027409)

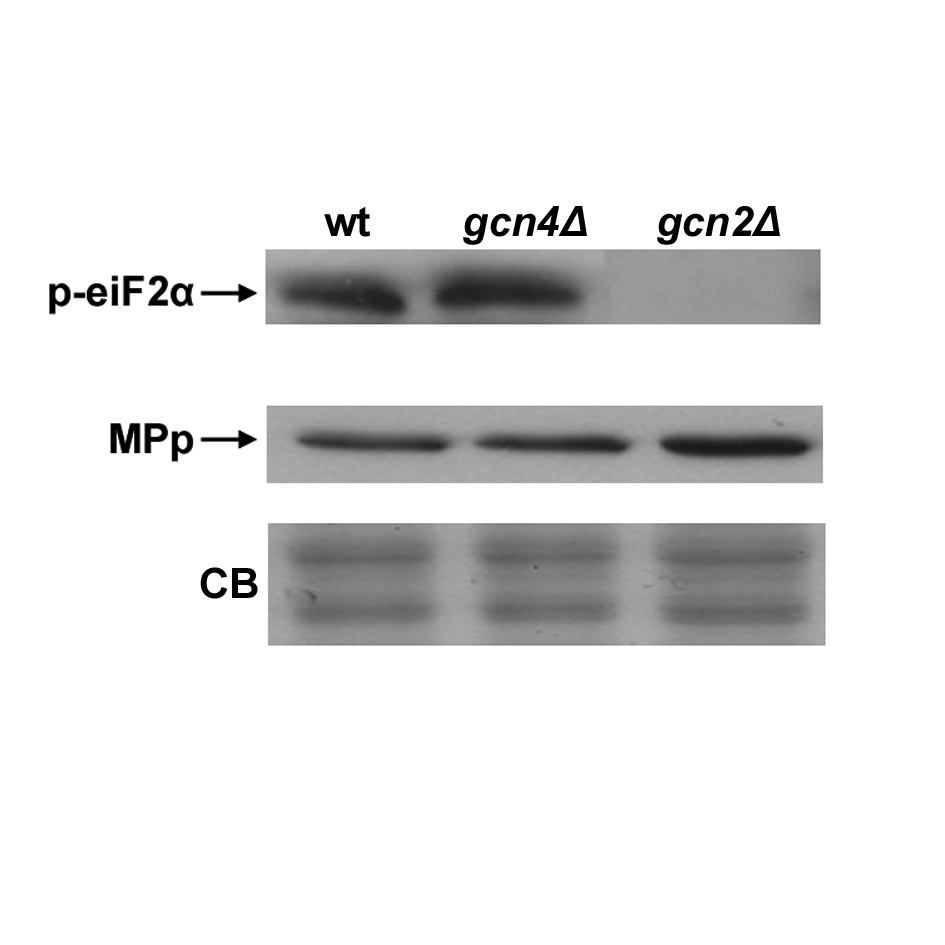

Supplement: Figure S1 — Analysis of eIF-2α phosphorylation in yeast gcn2Δ and gcn4Δ mutants. Immunodetection of phospho-eIF2α (p-eIF2α) and MPpnrsv (MPp) levels in protein extracts from wt, gcn4Δ and gcn2Δ yeast strains expressing MPpnrsv. Yeast cells were grown for3 hours in SD medium without DOX. MPpnrsv and phosphorylated eIF2α were detected as described in materials and methods. Coomassie Blue (CB) stained gel used as loading control. (TIF) [file pone.0027409.s001.tif]
