# Supplementary material for: A Plant Virus Movement Protein Regulates the Gcn2p Kinase in Budding Yeast
Source: PLoS One. 2011 Nov 8;6(11):e27409. doi: 10.1371/journal.pone.0027409 (PMC3210792; doi:10.1371/journal.pone.0027409)
Supplement: Table S1 — Functional classification of induced genes in MPpnrsv versus the empty vector expressing yeast strains. (DOC) [file pone.0027409.s002.doc]

**TABLE S1.** Functional classification of induced genes in MPpnrsv versus the empty vector expressing yeast strains

| ***Gene Ontology term*** | ***Cluster frequency*** | ***Genome frequency of use*** | ***Corrected***  ***P-value*** | ***Genes annotated to the term*** |
| --- | --- | --- | --- | --- |
| [*glutamine family amino acid metabolic process*](http://www.yeastgenome.org/cgi-bin/GO/goTerm.pl?goid=9064) | 12 out of 79 genes, 15.2% | 44 out of 7167 genes, 0.6% | 5.23e-12 | [GDH3/YAL062W](http://www.yeastgenome.org/cgi-bin/locus.fpl?locus=GDH3), [CIT2/YCR005C](http://www.yeastgenome.org/cgi-bin/locus.fpl?locus=CIT2), [GAP1/YKR039W](http://www.yeastgenome.org/cgi-bin/locus.fpl?locus=GAP1), [PUT1/YLR142W](http://www.yeastgenome.org/cgi-bin/locus.fpl?locus=PUT1), [ASP3-1/YLR155C](http://www.yeastgenome.org/cgi-bin/locus.fpl?locus=ASP3), [ASP3-2/YLR157C](http://www.yeastgenome.org/cgi-bin/locus.fpl?locus=ASP3), [ASP3-3/YLR158C](http://www.yeastgenome.org/cgi-bin/locus.fpl?locus=ASP3), [ASP3-4/YLR160C](http://www.yeastgenome.org/cgi-bin/locus.fpl?locus=ASP3), [CAR2/YLR438W](http://www.yeastgenome.org/cgi-bin/locus.fpl?locus=CAR2), [PUT4/YOR348C](http://www.yeastgenome.org/cgi-bin/locus.fpl?locus=PUT4), [GDH1/YOR375C](http://www.yeastgenome.org/cgi-bin/locus.fpl?locus=GDH1), [CAR1/YPL111W](http://www.yeastgenome.org/cgi-bin/locus.fpl?locus=CAR1) |
| [*amine metabolic process*](http://www.yeastgenome.org/cgi-bin/GO/goTerm.pl?goid=9308) | 22 out of 79 genes, 27.8% | 262 out of 7167 genes, 3.7% | 7.48e-12 | [GDH3/YAL062W](http://www.yeastgenome.org/cgi-bin/locus.fpl?locus=GDH3), [YAT1/YAR035W](http://www.yeastgenome.org/cgi-bin/locus.fpl?locus=YAT1), [DUR1,2/YBR208C](http://www.yeastgenome.org/cgi-bin/locus.fpl?locus=DUR1), [CIT2/YCR005C](http://www.yeastgenome.org/cgi-bin/locus.fpl?locus=CIT2), [UGA3/YDL170W](http://www.yeastgenome.org/cgi-bin/locus.fpl?locus=UGA3), [YAT2/YER024W](http://www.yeastgenome.org/cgi-bin/locus.fpl?locus=YAT2), [MET28/YIR017C](http://www.yeastgenome.org/cgi-bin/locus.fpl?locus=MET28), [DAL2/YIR029W](http://www.yeastgenome.org/cgi-bin/locus.fpl?locus=DAL2), [DAL7/YIR031C](http://www.yeastgenome.org/cgi-bin/locus.fpl?locus=DAL7), [DAL3/YIR032C](http://www.yeastgenome.org/cgi-bin/locus.fpl?locus=DAL3), [BAT2/YJR148W](http://www.yeastgenome.org/cgi-bin/locus.fpl?locus=BAT2), [GAP1/YKR039W](http://www.yeastgenome.org/cgi-bin/locus.fpl?locus=GAP1), [ALT1/YLR089C](http://www.yeastgenome.org/cgi-bin/locus.fpl?locus=ALT1), [PUT1/YLR142W](http://www.yeastgenome.org/cgi-bin/locus.fpl?locus=PUT1), [ASP3-1/YLR155C](http://www.yeastgenome.org/cgi-bin/locus.fpl?locus=ASP3), [ASP3-2/YLR157C](http://www.yeastgenome.org/cgi-bin/locus.fpl?locus=ASP3), [ASP3-3/YLR158C](http://www.yeastgenome.org/cgi-bin/locus.fpl?locus=ASP3), [ASP3-4/YLR160C](http://www.yeastgenome.org/cgi-bin/locus.fpl?locus=ASP3), [CAR2/YLR438W](http://www.yeastgenome.org/cgi-bin/locus.fpl?locus=CAR2), [PUT4/YOR348C](http://www.yeastgenome.org/cgi-bin/locus.fpl?locus=PUT4), [GDH1/YOR375C](http://www.yeastgenome.org/cgi-bin/locus.fpl?locus=GDH1), [CAR1/YPL111W](http://www.yeastgenome.org/cgi-bin/locus.fpl?locus=CAR1) |
| [*glutamine family amino acid catabolic process*](http://www.yeastgenome.org/cgi-bin/GO/goTerm.pl?goid=9065) | 8 out of 79 genes, 10.1% | 14 out of 7167 genes, 0.2% | 1.04e-10 | [PUT1/YLR142W](http://www.yeastgenome.org/cgi-bin/locus.fpl?locus=PUT1), [ASP3-1/YLR155C](http://www.yeastgenome.org/cgi-bin/locus.fpl?locus=ASP3), [ASP3-2/YLR157C](http://www.yeastgenome.org/cgi-bin/locus.fpl?locus=ASP3), [ASP3-3/YLR158C](http://www.yeastgenome.org/cgi-bin/locus.fpl?locus=ASP3), [ASP3-4/YLR160C](http://www.yeastgenome.org/cgi-bin/locus.fpl?locus=ASP3), [CAR2/YLR438W](http://www.yeastgenome.org/cgi-bin/locus.fpl?locus=CAR2), [PUT4/YOR348C](http://www.yeastgenome.org/cgi-bin/locus.fpl?locus=PUT4), [CAR1/YPL111W](http://www.yeastgenome.org/cgi-bin/locus.fpl?locus=CAR1) |
| [*organic acid catabolic process*](http://www.yeastgenome.org/cgi-bin/GO/goTerm.pl?goid=16054) | 11 out of 79 genes, 13.9% | 53 out of 7167 genes, 0.7% | 1.81e-09 | [BAT2/YJR148W](http://www.yeastgenome.org/cgi-bin/locus.fpl?locus=BAT2), [ALT1/YLR089C](http://www.yeastgenome.org/cgi-bin/locus.fpl?locus=ALT1), [PUT1/YLR142W](http://www.yeastgenome.org/cgi-bin/locus.fpl?locus=PUT1), [ASP3-1/YLR155C](http://www.yeastgenome.org/cgi-bin/locus.fpl?locus=ASP3), [ASP3-2/YLR157C](http://www.yeastgenome.org/cgi-bin/locus.fpl?locus=ASP3), [ASP3-3/YLR158C](http://www.yeastgenome.org/cgi-bin/locus.fpl?locus=ASP3), [ASP3-4/YLR160C](http://www.yeastgenome.org/cgi-bin/locus.fpl?locus=ASP3), [CAR2/YLR438W](http://www.yeastgenome.org/cgi-bin/locus.fpl?locus=CAR2), [PUT4/YOR348C](http://www.yeastgenome.org/cgi-bin/locus.fpl?locus=PUT4), [CAR1/YPL111W](http://www.yeastgenome.org/cgi-bin/locus.fpl?locus=CAR1), [CIT3/YPR001W](http://www.yeastgenome.org/cgi-bin/locus.fpl?locus=CIT3) |
| [*carboxylic acid catabolic process*](http://www.yeastgenome.org/cgi-bin/GO/goTerm.pl?goid=46395) | 11 out of 79 genes, 13.9% | 53 out of 7167 genes, 0.7% | 1.81e-09 | [BAT2/YJR148W](http://www.yeastgenome.org/cgi-bin/locus.fpl?locus=BAT2), [ALT1/YLR089C](http://www.yeastgenome.org/cgi-bin/locus.fpl?locus=ALT1), [PUT1/YLR142W](http://www.yeastgenome.org/cgi-bin/locus.fpl?locus=PUT1), [ASP3-1/YLR155C](http://www.yeastgenome.org/cgi-bin/locus.fpl?locus=ASP3), [ASP3-2/YLR157C](http://www.yeastgenome.org/cgi-bin/locus.fpl?locus=ASP3), [ASP3-3/YLR158C](http://www.yeastgenome.org/cgi-bin/locus.fpl?locus=ASP3), [ASP3-4/YLR160C](http://www.yeastgenome.org/cgi-bin/locus.fpl?locus=ASP3), [CAR2/YLR438W](http://www.yeastgenome.org/cgi-bin/locus.fpl?locus=CAR2), [PUT4/YOR348C](http://www.yeastgenome.org/cgi-bin/locus.fpl?locus=PUT4), [CAR1/YPL111W](http://www.yeastgenome.org/cgi-bin/locus.fpl?locus=CAR1), [CIT3/YPR001W](http://www.yeastgenome.org/cgi-bin/locus.fpl?locus=CIT3) |
| [*carboxylic acid metabolic process*](http://www.yeastgenome.org/cgi-bin/GO/goTerm.pl?goid=19752) | 22 out of 79 genes, 27.8% | 344 out of 7167 genes, 4.8% | 1.94e-09 | [GDH3/YAL062W](http://www.yeastgenome.org/cgi-bin/locus.fpl?locus=GDH3), [YAT1/YAR035W](http://www.yeastgenome.org/cgi-bin/locus.fpl?locus=YAT1), [ACH1/YBL015W](http://www.yeastgenome.org/cgi-bin/locus.fpl?locus=ACH1), [CIT2/YCR005C](http://www.yeastgenome.org/cgi-bin/locus.fpl?locus=CIT2), [DLD3/YEL071W](http://www.yeastgenome.org/cgi-bin/locus.fpl?locus=DLD3), [YAT2/YER024W](http://www.yeastgenome.org/cgi-bin/locus.fpl?locus=YAT2), [PYC1/YGL062W](http://www.yeastgenome.org/cgi-bin/locus.fpl?locus=PYC1), [MET28/YIR017C](http://www.yeastgenome.org/cgi-bin/locus.fpl?locus=MET28), [BAT2/YJR148W](http://www.yeastgenome.org/cgi-bin/locus.fpl?locus=BAT2), [GAP1/YKR039W](http://www.yeastgenome.org/cgi-bin/locus.fpl?locus=GAP1), [ALT1/YLR089C](http://www.yeastgenome.org/cgi-bin/locus.fpl?locus=ALT1), [PUT1/YLR142W](http://www.yeastgenome.org/cgi-bin/locus.fpl?locus=PUT1), [ASP3-1/YLR155C](http://www.yeastgenome.org/cgi-bin/locus.fpl?locus=ASP3), [ASP3-2/YLR157C](http://www.yeastgenome.org/cgi-bin/locus.fpl?locus=ASP3), [ASP3-3/YLR158C](http://www.yeastgenome.org/cgi-bin/locus.fpl?locus=ASP3), [ASP3-4/YLR160C](http://www.yeastgenome.org/cgi-bin/locus.fpl?locus=ASP3), [CAR2/YLR438W](http://www.yeastgenome.org/cgi-bin/locus.fpl?locus=CAR2), [MDH2/YOL126C](http://www.yeastgenome.org/cgi-bin/locus.fpl?locus=MDH2), [PUT4/YOR348C](http://www.yeastgenome.org/cgi-bin/locus.fpl?locus=PUT4), [GDH1/YOR375C](http://www.yeastgenome.org/cgi-bin/locus.fpl?locus=GDH1), [CAR1/YPL111W](http://www.yeastgenome.org/cgi-bin/locus.fpl?locus=CAR1), [CIT3/YPR001W](http://www.yeastgenome.org/cgi-bin/locus.fpl?locus=CIT3) |
| [*oxoacid metabolic process*](http://www.yeastgenome.org/cgi-bin/GO/goTerm.pl?goid=43436) | 22 out of 79 genes, 27.8% | 344 out of 7167 genes, 4.8% | 1.94e-09 | [GDH3/YAL062W](http://www.yeastgenome.org/cgi-bin/locus.fpl?locus=GDH3), [YAT1/YAR035W](http://www.yeastgenome.org/cgi-bin/locus.fpl?locus=YAT1), [ACH1/YBL015W](http://www.yeastgenome.org/cgi-bin/locus.fpl?locus=ACH1), [CIT2/YCR005C](http://www.yeastgenome.org/cgi-bin/locus.fpl?locus=CIT2), [DLD3/YEL071W](http://www.yeastgenome.org/cgi-bin/locus.fpl?locus=DLD3), [YAT2/YER024W](http://www.yeastgenome.org/cgi-bin/locus.fpl?locus=YAT2), [PYC1/YGL062W](http://www.yeastgenome.org/cgi-bin/locus.fpl?locus=PYC1), [MET28/YIR017C](http://www.yeastgenome.org/cgi-bin/locus.fpl?locus=MET28), [BAT2/YJR148W](http://www.yeastgenome.org/cgi-bin/locus.fpl?locus=BAT2), [GAP1/YKR039W](http://www.yeastgenome.org/cgi-bin/locus.fpl?locus=GAP1), [ALT1/YLR089C](http://www.yeastgenome.org/cgi-bin/locus.fpl?locus=ALT1), [PUT1/YLR142W](http://www.yeastgenome.org/cgi-bin/locus.fpl?locus=PUT1), [ASP3-1/YLR155C](http://www.yeastgenome.org/cgi-bin/locus.fpl?locus=ASP3), [ASP3-2/YLR157C](http://www.yeastgenome.org/cgi-bin/locus.fpl?locus=ASP3), [ASP3-3/YLR158C](http://www.yeastgenome.org/cgi-bin/locus.fpl?locus=ASP3), [ASP3-4/YLR160C](http://www.yeastgenome.org/cgi-bin/locus.fpl?locus=ASP3), [CAR2/YLR438W](http://www.yeastgenome.org/cgi-bin/locus.fpl?locus=CAR2), [MDH2/YOL126C](http://www.yeastgenome.org/cgi-bin/locus.fpl?locus=MDH2), [PUT4/YOR348C](http://www.yeastgenome.org/cgi-bin/locus.fpl?locus=PUT4), [GDH1/YOR375C](http://www.yeastgenome.org/cgi-bin/locus.fpl?locus=GDH1), [CAR1/YPL111W](http://www.yeastgenome.org/cgi-bin/locus.fpl?locus=CAR1), [CIT3/YPR001W](http://www.yeastgenome.org/cgi-bin/locus.fpl?locus=CIT3) |
| [*organic acid metabolic process*](http://www.yeastgenome.org/cgi-bin/GO/goTerm.pl?goid=6082) | 22 out of 79 genes, 27.8% | 345 out of 7167 genes, 4.8% | 2.06e-09 | [GDH3/YAL062W](http://www.yeastgenome.org/cgi-bin/locus.fpl?locus=GDH3), [YAT1/YAR035W](http://www.yeastgenome.org/cgi-bin/locus.fpl?locus=YAT1), [ACH1/YBL015W](http://www.yeastgenome.org/cgi-bin/locus.fpl?locus=ACH1), [CIT2/YCR005C](http://www.yeastgenome.org/cgi-bin/locus.fpl?locus=CIT2), [DLD3/YEL071W](http://www.yeastgenome.org/cgi-bin/locus.fpl?locus=DLD3), [YAT2/YER024W](http://www.yeastgenome.org/cgi-bin/locus.fpl?locus=YAT2), [PYC1/YGL062W](http://www.yeastgenome.org/cgi-bin/locus.fpl?locus=PYC1), [MET28/YIR017C](http://www.yeastgenome.org/cgi-bin/locus.fpl?locus=MET28), [BAT2/YJR148W](http://www.yeastgenome.org/cgi-bin/locus.fpl?locus=BAT2), [GAP1/YKR039W](http://www.yeastgenome.org/cgi-bin/locus.fpl?locus=GAP1), [ALT1/YLR089C](http://www.yeastgenome.org/cgi-bin/locus.fpl?locus=ALT1), [PUT1/YLR142W](http://www.yeastgenome.org/cgi-bin/locus.fpl?locus=PUT1), [ASP3-1/YLR155C](http://www.yeastgenome.org/cgi-bin/locus.fpl?locus=ASP3), [ASP3-2/YLR157C](http://www.yeastgenome.org/cgi-bin/locus.fpl?locus=ASP3), [ASP3-3/YLR158C](http://www.yeastgenome.org/cgi-bin/locus.fpl?locus=ASP3), [ASP3-4/YLR160C](http://www.yeastgenome.org/cgi-bin/locus.fpl?locus=ASP3), [CAR2/YLR438W](http://www.yeastgenome.org/cgi-bin/locus.fpl?locus=CAR2), [MDH2/YOL126C](http://www.yeastgenome.org/cgi-bin/locus.fpl?locus=MDH2), [PUT4/YOR348C](http://www.yeastgenome.org/cgi-bin/locus.fpl?locus=PUT4), [GDH1/YOR375C](http://www.yeastgenome.org/cgi-bin/locus.fpl?locus=GDH1), [CAR1/YPL111W](http://www.yeastgenome.org/cgi-bin/locus.fpl?locus=CAR1), [CIT3/YPR001W](http://www.yeastgenome.org/cgi-bin/locus.fpl?locus=CIT3) |
| [*cellular amino acid catabolic process*](http://www.yeastgenome.org/cgi-bin/GO/goTerm.pl?goid=9063) | 10 out of 79 genes, 12.7% | 41 out of 7167 genes, 0.6% | 3.05e-09 | [BAT2/YJR148W](http://www.yeastgenome.org/cgi-bin/locus.fpl?locus=BAT2), [ALT1/YLR089C](http://www.yeastgenome.org/cgi-bin/locus.fpl?locus=ALT1), [PUT1/YLR142W](http://www.yeastgenome.org/cgi-bin/locus.fpl?locus=PUT1), [ASP3-1/YLR155C](http://www.yeastgenome.org/cgi-bin/locus.fpl?locus=ASP3), [ASP3-2/YLR157C](http://www.yeastgenome.org/cgi-bin/locus.fpl?locus=ASP3), [ASP3-3/YLR158C](http://www.yeastgenome.org/cgi-bin/locus.fpl?locus=ASP3), [ASP3-4/YLR160C](http://www.yeastgenome.org/cgi-bin/locus.fpl?locus=ASP3), [CAR2/YLR438W](http://www.yeastgenome.org/cgi-bin/locus.fpl?locus=CAR2), [PUT4/YOR348C](http://www.yeastgenome.org/cgi-bin/locus.fpl?locus=PUT4), [CAR1/YPL111W](http://www.yeastgenome.org/cgi-bin/locus.fpl?locus=CAR1) |
| [*cellular ketone metabolic process*](http://www.yeastgenome.org/cgi-bin/GO/goTerm.pl?goid=42180) | 22 out of 79 genes, 27.8% | 359 out of 7167 genes, 5.0% | 4.55e-09 | [GDH3/YAL062W](http://www.yeastgenome.org/cgi-bin/locus.fpl?locus=GDH3), [YAT1/YAR035W](http://www.yeastgenome.org/cgi-bin/locus.fpl?locus=YAT1), [ACH1/YBL015W](http://www.yeastgenome.org/cgi-bin/locus.fpl?locus=ACH1), [CIT2/YCR005C](http://www.yeastgenome.org/cgi-bin/locus.fpl?locus=CIT2), [DLD3/YEL071W](http://www.yeastgenome.org/cgi-bin/locus.fpl?locus=DLD3), [YAT2/YER024W](http://www.yeastgenome.org/cgi-bin/locus.fpl?locus=YAT2), [PYC1/YGL062W](http://www.yeastgenome.org/cgi-bin/locus.fpl?locus=PYC1), [MET28/YIR017C](http://www.yeastgenome.org/cgi-bin/locus.fpl?locus=MET28), [BAT2/YJR148W](http://www.yeastgenome.org/cgi-bin/locus.fpl?locus=BAT2), [GAP1/YKR039W](http://www.yeastgenome.org/cgi-bin/locus.fpl?locus=GAP1), [ALT1/YLR089C](http://www.yeastgenome.org/cgi-bin/locus.fpl?locus=ALT1), [PUT1/YLR142W](http://www.yeastgenome.org/cgi-bin/locus.fpl?locus=PUT1), [ASP3-1/YLR155C](http://www.yeastgenome.org/cgi-bin/locus.fpl?locus=ASP3), [ASP3-2/YLR157C](http://www.yeastgenome.org/cgi-bin/locus.fpl?locus=ASP3), [ASP3-3/YLR158C](http://www.yeastgenome.org/cgi-bin/locus.fpl?locus=ASP3), [ASP3-4/YLR160C](http://www.yeastgenome.org/cgi-bin/locus.fpl?locus=ASP3), [CAR2/YLR438W](http://www.yeastgenome.org/cgi-bin/locus.fpl?locus=CAR2), [MDH2/YOL126C](http://www.yeastgenome.org/cgi-bin/locus.fpl?locus=MDH2), [PUT4/YOR348C](http://www.yeastgenome.org/cgi-bin/locus.fpl?locus=PUT4), [GDH1/YOR375C](http://www.yeastgenome.org/cgi-bin/locus.fpl?locus=GDH1), [CAR1/YPL111W](http://www.yeastgenome.org/cgi-bin/locus.fpl?locus=CAR1), [CIT3/YPR001W](http://www.yeastgenome.org/cgi-bin/locus.fpl?locus=CIT3) |
| [*amine catabolic process*](http://www.yeastgenome.org/cgi-bin/GO/goTerm.pl?goid=9310) | 10 out of 79 genes, 12.7% | 44 out of 7167 genes, 0.6% | 6.57e-09 | [BAT2/YJR148W](http://www.yeastgenome.org/cgi-bin/locus.fpl?locus=BAT2), [ALT1/YLR089C](http://www.yeastgenome.org/cgi-bin/locus.fpl?locus=ALT1), [PUT1/YLR142W](http://www.yeastgenome.org/cgi-bin/locus.fpl?locus=PUT1), [ASP3-1/YLR155C](http://www.yeastgenome.org/cgi-bin/locus.fpl?locus=ASP3), [ASP3-2/YLR157C](http://www.yeastgenome.org/cgi-bin/locus.fpl?locus=ASP3), [ASP3-3/YLR158C](http://www.yeastgenome.org/cgi-bin/locus.fpl?locus=ASP3), [ASP3-4/YLR160C](http://www.yeastgenome.org/cgi-bin/locus.fpl?locus=ASP3), [CAR2/YLR438W](http://www.yeastgenome.org/cgi-bin/locus.fpl?locus=CAR2), [PUT4/YOR348C](http://www.yeastgenome.org/cgi-bin/locus.fpl?locus=PUT4), [CAR1/YPL111W](http://www.yeastgenome.org/cgi-bin/locus.fpl?locus=CAR1) |
| [*cellular amino acid and derivative metabolic process*](http://www.yeastgenome.org/cgi-bin/GO/goTerm.pl?goid=6519) | 18 out of 79 genes, 22.8% | 255 out of 7167 genes, 3.6% | 4.39e-08 | [GDH3/YAL062W](http://www.yeastgenome.org/cgi-bin/locus.fpl?locus=GDH3), [YAT1/YAR035W](http://www.yeastgenome.org/cgi-bin/locus.fpl?locus=YAT1), [CIT2/YCR005C](http://www.yeastgenome.org/cgi-bin/locus.fpl?locus=CIT2), [UGA3/YDL170W](http://www.yeastgenome.org/cgi-bin/locus.fpl?locus=UGA3), [YAT2/YER024W](http://www.yeastgenome.org/cgi-bin/locus.fpl?locus=YAT2), [MET28/YIR017C](http://www.yeastgenome.org/cgi-bin/locus.fpl?locus=MET28), [BAT2/YJR148W](http://www.yeastgenome.org/cgi-bin/locus.fpl?locus=BAT2), [GAP1/YKR039W](http://www.yeastgenome.org/cgi-bin/locus.fpl?locus=GAP1), [ALT1/YLR089C](http://www.yeastgenome.org/cgi-bin/locus.fpl?locus=ALT1), [PUT1/YLR142W](http://www.yeastgenome.org/cgi-bin/locus.fpl?locus=PUT1), [ASP3-1/YLR155C](http://www.yeastgenome.org/cgi-bin/locus.fpl?locus=ASP3), [ASP3-2/YLR157C](http://www.yeastgenome.org/cgi-bin/locus.fpl?locus=ASP3), [ASP3-3/YLR158C](http://www.yeastgenome.org/cgi-bin/locus.fpl?locus=ASP3), [ASP3-4/YLR160C](http://www.yeastgenome.org/cgi-bin/locus.fpl?locus=ASP3), [CAR2/YLR438W](http://www.yeastgenome.org/cgi-bin/locus.fpl?locus=CAR2), [PUT4/YOR348C](http://www.yeastgenome.org/cgi-bin/locus.fpl?locus=PUT4), [GDH1/YOR375C](http://www.yeastgenome.org/cgi-bin/locus.fpl?locus=GDH1), [CAR1/YPL111W](http://www.yeastgenome.org/cgi-bin/locus.fpl?locus=CAR1) |
| [*cellular amine metabolic process*](http://www.yeastgenome.org/cgi-bin/GO/goTerm.pl?goid=44106) | 17 out of 79 genes, 21.5% | 232 out of 7167 genes, 3.2% | 8.54e-08 | [GDH3/YAL062W](http://www.yeastgenome.org/cgi-bin/locus.fpl?locus=GDH3), [YAT1/YAR035W](http://www.yeastgenome.org/cgi-bin/locus.fpl?locus=YAT1), [CIT2/YCR005C](http://www.yeastgenome.org/cgi-bin/locus.fpl?locus=CIT2), [YAT2/YER024W](http://www.yeastgenome.org/cgi-bin/locus.fpl?locus=YAT2), [MET28/YIR017C](http://www.yeastgenome.org/cgi-bin/locus.fpl?locus=MET28), [BAT2/YJR148W](http://www.yeastgenome.org/cgi-bin/locus.fpl?locus=BAT2), [GAP1/YKR039W](http://www.yeastgenome.org/cgi-bin/locus.fpl?locus=GAP1), [ALT1/YLR089C](http://www.yeastgenome.org/cgi-bin/locus.fpl?locus=ALT1), [PUT1/YLR142W](http://www.yeastgenome.org/cgi-bin/locus.fpl?locus=PUT1), [ASP3-1/YLR155C](http://www.yeastgenome.org/cgi-bin/locus.fpl?locus=ASP3), [ASP3-2/YLR157C](http://www.yeastgenome.org/cgi-bin/locus.fpl?locus=ASP3), [ASP3-3/YLR158C](http://www.yeastgenome.org/cgi-bin/locus.fpl?locus=ASP3), [ASP3-4/YLR160C](http://www.yeastgenome.org/cgi-bin/locus.fpl?locus=ASP3), [CAR2/YLR438W](http://www.yeastgenome.org/cgi-bin/locus.fpl?locus=CAR2), [PUT4/YOR348C](http://www.yeastgenome.org/cgi-bin/locus.fpl?locus=PUT4), [GDH1/YOR375C](http://www.yeastgenome.org/cgi-bin/locus.fpl?locus=GDH1), [CAR1/YPL111W](http://www.yeastgenome.org/cgi-bin/locus.fpl?locus=CAR1) |
| [*small molecule catabolic process*](http://www.yeastgenome.org/cgi-bin/GO/goTerm.pl?goid=44282) | 14 out of 79 genes, 17.7% | 144 out of 7167 genes, 2.0% | 9.14e-08 | [DUR1,2/YBR208C](http://www.yeastgenome.org/cgi-bin/locus.fpl?locus=DUR1), [UGA3/YDL170W](http://www.yeastgenome.org/cgi-bin/locus.fpl?locus=UGA3), [DUR3/YHL016C](http://www.yeastgenome.org/cgi-bin/locus.fpl?locus=DUR3), [BAT2/YJR148W](http://www.yeastgenome.org/cgi-bin/locus.fpl?locus=BAT2), [ALT1/YLR089C](http://www.yeastgenome.org/cgi-bin/locus.fpl?locus=ALT1), [PUT1/YLR142W](http://www.yeastgenome.org/cgi-bin/locus.fpl?locus=PUT1), [ASP3-1/YLR155C](http://www.yeastgenome.org/cgi-bin/locus.fpl?locus=ASP3), [ASP3-2/YLR157C](http://www.yeastgenome.org/cgi-bin/locus.fpl?locus=ASP3), [ASP3-3/YLR158C](http://www.yeastgenome.org/cgi-bin/locus.fpl?locus=ASP3), [ASP3-4/YLR160C](http://www.yeastgenome.org/cgi-bin/locus.fpl?locus=ASP3), [CAR2/YLR438W](http://www.yeastgenome.org/cgi-bin/locus.fpl?locus=CAR2), [PUT4/YOR348C](http://www.yeastgenome.org/cgi-bin/locus.fpl?locus=PUT4), [CAR1/YPL111W](http://www.yeastgenome.org/cgi-bin/locus.fpl?locus=CAR1), [CIT3/YPR001W](http://www.yeastgenome.org/cgi-bin/locus.fpl?locus=CIT3) |
| [*cellular catabolic process*](http://www.yeastgenome.org/cgi-bin/GO/goTerm.pl?goid=44248) | 23 out of 79 genes, 29.1% | 529 out of 7167 genes, 7.4% | 1.36e-06 | [DUR1,2/YBR208C](http://www.yeastgenome.org/cgi-bin/locus.fpl?locus=DUR1), [UGA3/YDL170W](http://www.yeastgenome.org/cgi-bin/locus.fpl?locus=UGA3), [PRB1/YEL060C](http://www.yeastgenome.org/cgi-bin/locus.fpl?locus=PRB1), [DUR3/YHL016C](http://www.yeastgenome.org/cgi-bin/locus.fpl?locus=DUR3), [MET30/YIL046W](http://www.yeastgenome.org/cgi-bin/locus.fpl?locus=MET30), [DAL2/YIR029W](http://www.yeastgenome.org/cgi-bin/locus.fpl?locus=DAL2), [DAL7/YIR031C](http://www.yeastgenome.org/cgi-bin/locus.fpl?locus=DAL7), [DAL3/YIR032C](http://www.yeastgenome.org/cgi-bin/locus.fpl?locus=DAL3), [BAT2/YJR148W](http://www.yeastgenome.org/cgi-bin/locus.fpl?locus=BAT2), [LAP4/YKL103C](http://www.yeastgenome.org/cgi-bin/locus.fpl?locus=LAP4), [ALT1/YLR089C](http://www.yeastgenome.org/cgi-bin/locus.fpl?locus=ALT1), [TIS11/YLR136C](http://www.yeastgenome.org/cgi-bin/locus.fpl?locus=TIS11), [PUT1/YLR142W](http://www.yeastgenome.org/cgi-bin/locus.fpl?locus=PUT1), [ASP3-1/YLR155C](http://www.yeastgenome.org/cgi-bin/locus.fpl?locus=ASP3), [ASP3-2/YLR157C](http://www.yeastgenome.org/cgi-bin/locus.fpl?locus=ASP3), [ASP3-3/YLR158C](http://www.yeastgenome.org/cgi-bin/locus.fpl?locus=ASP3), [ASP3-4/YLR160C](http://www.yeastgenome.org/cgi-bin/locus.fpl?locus=ASP3), [HMX1/YLR205C](http://www.yeastgenome.org/cgi-bin/locus.fpl?locus=HMX1), [CAR2/YLR438W](http://www.yeastgenome.org/cgi-bin/locus.fpl?locus=CAR2), [DSS1/YMR287C](http://www.yeastgenome.org/cgi-bin/locus.fpl?locus=DSS1), [PUT4/YOR348C](http://www.yeastgenome.org/cgi-bin/locus.fpl?locus=PUT4), [CAR1/YPL111W](http://www.yeastgenome.org/cgi-bin/locus.fpl?locus=CAR1), [CIT3/YPR001W](http://www.yeastgenome.org/cgi-bin/locus.fpl?locus=CIT3) |
| [*cellular amino acid metabolic process*](http://www.yeastgenome.org/cgi-bin/GO/goTerm.pl?goid=6520) | 15 out of 79 genes, 19.0% | 210 out of 7167 genes, 2.9% | 1.52e-06 | [GDH3/YAL062W](http://www.yeastgenome.org/cgi-bin/locus.fpl?locus=GDH3), [CIT2/YCR005C](http://www.yeastgenome.org/cgi-bin/locus.fpl?locus=CIT2), [MET28/YIR017C](http://www.yeastgenome.org/cgi-bin/locus.fpl?locus=MET28), [BAT2/YJR148W](http://www.yeastgenome.org/cgi-bin/locus.fpl?locus=BAT2), [GAP1/YKR039W](http://www.yeastgenome.org/cgi-bin/locus.fpl?locus=GAP1), [ALT1/YLR089C](http://www.yeastgenome.org/cgi-bin/locus.fpl?locus=ALT1), [PUT1/YLR142W](http://www.yeastgenome.org/cgi-bin/locus.fpl?locus=PUT1), [ASP3-1/YLR155C](http://www.yeastgenome.org/cgi-bin/locus.fpl?locus=ASP3), [ASP3-2/YLR157C](http://www.yeastgenome.org/cgi-bin/locus.fpl?locus=ASP3), [ASP3-3/YLR158C](http://www.yeastgenome.org/cgi-bin/locus.fpl?locus=ASP3), [ASP3-4/YLR160C](http://www.yeastgenome.org/cgi-bin/locus.fpl?locus=ASP3), [CAR2/YLR438W](http://www.yeastgenome.org/cgi-bin/locus.fpl?locus=CAR2), [PUT4/YOR348C](http://www.yeastgenome.org/cgi-bin/locus.fpl?locus=PUT4), [GDH1/YOR375C](http://www.yeastgenome.org/cgi-bin/locus.fpl?locus=GDH1), [CAR1/YPL111W](http://www.yeastgenome.org/cgi-bin/locus.fpl?locus=CAR1) |
| [*cellular amide catabolic process*](http://www.yeastgenome.org/cgi-bin/GO/goTerm.pl?goid=43605) | 5 out of 79 genes, 6.3% | 8 out of 7167 genes, 0.1% | 1.89e-06 | [DUR1,2/YBR208C](http://www.yeastgenome.org/cgi-bin/locus.fpl?locus=DUR1), [DUR3/YHL016C](http://www.yeastgenome.org/cgi-bin/locus.fpl?locus=DUR3), [DAL2/YIR029W](http://www.yeastgenome.org/cgi-bin/locus.fpl?locus=DAL2), [DAL7/YIR031C](http://www.yeastgenome.org/cgi-bin/locus.fpl?locus=DAL7), [DAL3/YIR032C](http://www.yeastgenome.org/cgi-bin/locus.fpl?locus=DAL3) |
| [*glutamate metabolic process*](http://www.yeastgenome.org/cgi-bin/GO/goTerm.pl?goid=6536) | 6 out of 79 genes, 7.6% | 17 out of 7167 genes, 0.2% | 4.04e-06 | [GDH3/YAL062W](http://www.yeastgenome.org/cgi-bin/locus.fpl?locus=GDH3), [CIT2/YCR005C](http://www.yeastgenome.org/cgi-bin/locus.fpl?locus=CIT2), [GAP1/YKR039W](http://www.yeastgenome.org/cgi-bin/locus.fpl?locus=GAP1), [PUT1/YLR142W](http://www.yeastgenome.org/cgi-bin/locus.fpl?locus=PUT1), [PUT4/YOR348C](http://www.yeastgenome.org/cgi-bin/locus.fpl?locus=PUT4), [GDH1/YOR375C](http://www.yeastgenome.org/cgi-bin/locus.fpl?locus=GDH1) |
| [*ammonia assimilation cycle*](http://www.yeastgenome.org/cgi-bin/GO/goTerm.pl?goid=19676) | 4 out of 79 genes, 5.1% | 5 out of 7167 genes, 0.1% | 1.64e-05 | [GDH3/YAL062W](http://www.yeastgenome.org/cgi-bin/locus.fpl?locus=GDH3), [GAP1/YKR039W](http://www.yeastgenome.org/cgi-bin/locus.fpl?locus=GAP1), [PUT4/YOR348C](http://www.yeastgenome.org/cgi-bin/locus.fpl?locus=PUT4), [GDH1/YOR375C](http://www.yeastgenome.org/cgi-bin/locus.fpl?locus=GDH1) |
| [*asparagine catabolic process*](http://www.yeastgenome.org/cgi-bin/GO/goTerm.pl?goid=6530) | 4 out of 79 genes, 5.1% | 5 out of 7167 genes, 0.1% | 1.64e-05 | [ASP3-1/YLR155C](http://www.yeastgenome.org/cgi-bin/locus.fpl?locus=ASP3), [ASP3-2/YLR157C](http://www.yeastgenome.org/cgi-bin/locus.fpl?locus=ASP3), [ASP3-3/YLR158C](http://www.yeastgenome.org/cgi-bin/locus.fpl?locus=ASP3), [ASP3-4/YLR160C](http://www.yeastgenome.org/cgi-bin/locus.fpl?locus=ASP3) |
| [*small molecule metabolic process*](http://www.yeastgenome.org/cgi-bin/GO/goTerm.pl?goid=44281) | 27 out of 79 genes, 34.2% | 839 out of 7167 genes, 11.7% | 2.87e-05 | [GDH3/YAL062W](http://www.yeastgenome.org/cgi-bin/locus.fpl?locus=GDH3), [YAT1/YAR035W](http://www.yeastgenome.org/cgi-bin/locus.fpl?locus=YAT1), [ACH1/YBL015W](http://www.yeastgenome.org/cgi-bin/locus.fpl?locus=ACH1), [DUR1,2/YBR208C](http://www.yeastgenome.org/cgi-bin/locus.fpl?locus=DUR1), [CIT2/YCR005C](http://www.yeastgenome.org/cgi-bin/locus.fpl?locus=CIT2), [UGA3/YDL170W](http://www.yeastgenome.org/cgi-bin/locus.fpl?locus=UGA3), [DLD3/YEL071W](http://www.yeastgenome.org/cgi-bin/locus.fpl?locus=DLD3), [YAT2/YER024W](http://www.yeastgenome.org/cgi-bin/locus.fpl?locus=YAT2), [PYC1/YGL062W](http://www.yeastgenome.org/cgi-bin/locus.fpl?locus=PYC1), [DUR3/YHL016C](http://www.yeastgenome.org/cgi-bin/locus.fpl?locus=DUR3), [MET28/YIR017C](http://www.yeastgenome.org/cgi-bin/locus.fpl?locus=MET28), [BAT2/YJR148W](http://www.yeastgenome.org/cgi-bin/locus.fpl?locus=BAT2), [URA1/YKL216W](http://www.yeastgenome.org/cgi-bin/locus.fpl?locus=URA1), [GAP1/YKR039W](http://www.yeastgenome.org/cgi-bin/locus.fpl?locus=GAP1), [ALT1/YLR089C](http://www.yeastgenome.org/cgi-bin/locus.fpl?locus=ALT1), [PUT1/YLR142W](http://www.yeastgenome.org/cgi-bin/locus.fpl?locus=PUT1), [ASP3-1/YLR155C](http://www.yeastgenome.org/cgi-bin/locus.fpl?locus=ASP3), [ASP3-2/YLR157C](http://www.yeastgenome.org/cgi-bin/locus.fpl?locus=ASP3), [ASP3-3/YLR158C](http://www.yeastgenome.org/cgi-bin/locus.fpl?locus=ASP3), [ASP3-4/YLR160C](http://www.yeastgenome.org/cgi-bin/locus.fpl?locus=ASP3), [CAR2/YLR438W](http://www.yeastgenome.org/cgi-bin/locus.fpl?locus=CAR2), [MDH2/YOL126C](http://www.yeastgenome.org/cgi-bin/locus.fpl?locus=MDH2), [RIB4/YOL143C](http://www.yeastgenome.org/cgi-bin/locus.fpl?locus=RIB4), [PUT4/YOR348C](http://www.yeastgenome.org/cgi-bin/locus.fpl?locus=PUT4), [GDH1/YOR375C](http://www.yeastgenome.org/cgi-bin/locus.fpl?locus=GDH1), [CAR1/YPL111W](http://www.yeastgenome.org/cgi-bin/locus.fpl?locus=CAR1), [CIT3/YPR001W](http://www.yeastgenome.org/cgi-bin/locus.fpl?locus=CIT3) |
| [*catabolic process*](http://www.yeastgenome.org/cgi-bin/GO/goTerm.pl?goid=9056) | 23 out of 79 genes, 29.1% | 624 out of 7167 genes, 8.7% | 3.05e-05 | [DUR1,2/YBR208C](http://www.yeastgenome.org/cgi-bin/locus.fpl?locus=DUR1), [UGA3/YDL170W](http://www.yeastgenome.org/cgi-bin/locus.fpl?locus=UGA3), [PRB1/YEL060C](http://www.yeastgenome.org/cgi-bin/locus.fpl?locus=PRB1), [DUR3/YHL016C](http://www.yeastgenome.org/cgi-bin/locus.fpl?locus=DUR3), [MET30/YIL046W](http://www.yeastgenome.org/cgi-bin/locus.fpl?locus=MET30), [DAL2/YIR029W](http://www.yeastgenome.org/cgi-bin/locus.fpl?locus=DAL2), [DAL7/YIR031C](http://www.yeastgenome.org/cgi-bin/locus.fpl?locus=DAL7), [DAL3/YIR032C](http://www.yeastgenome.org/cgi-bin/locus.fpl?locus=DAL3), [BAT2/YJR148W](http://www.yeastgenome.org/cgi-bin/locus.fpl?locus=BAT2), [LAP4/YKL103C](http://www.yeastgenome.org/cgi-bin/locus.fpl?locus=LAP4), [ALT1/YLR089C](http://www.yeastgenome.org/cgi-bin/locus.fpl?locus=ALT1), [TIS11/YLR136C](http://www.yeastgenome.org/cgi-bin/locus.fpl?locus=TIS11), [PUT1/YLR142W](http://www.yeastgenome.org/cgi-bin/locus.fpl?locus=PUT1), [ASP3-1/YLR155C](http://www.yeastgenome.org/cgi-bin/locus.fpl?locus=ASP3), [ASP3-2/YLR157C](http://www.yeastgenome.org/cgi-bin/locus.fpl?locus=ASP3), [ASP3-3/YLR158C](http://www.yeastgenome.org/cgi-bin/locus.fpl?locus=ASP3), [ASP3-4/YLR160C](http://www.yeastgenome.org/cgi-bin/locus.fpl?locus=ASP3), [HMX1/YLR205C](http://www.yeastgenome.org/cgi-bin/locus.fpl?locus=HMX1), [CAR2/YLR438W](http://www.yeastgenome.org/cgi-bin/locus.fpl?locus=CAR2), [DSS1/YMR287C](http://www.yeastgenome.org/cgi-bin/locus.fpl?locus=DSS1), [PUT4/YOR348C](http://www.yeastgenome.org/cgi-bin/locus.fpl?locus=PUT4), [CAR1/YPL111W](http://www.yeastgenome.org/cgi-bin/locus.fpl?locus=CAR1), [CIT3/YPR001W](http://www.yeastgenome.org/cgi-bin/locus.fpl?locus=CIT3) |
| [*cellular response to nitrogen levels*](http://www.yeastgenome.org/cgi-bin/GO/goTerm.pl?goid=43562) | 4 out of 79 genes, 5.1% | 6 out of 7167 genes, 0.1% | 4.90e-05 | [ASP3-1/YLR155C](http://www.yeastgenome.org/cgi-bin/locus.fpl?locus=ASP3), [ASP3-2/YLR157C](http://www.yeastgenome.org/cgi-bin/locus.fpl?locus=ASP3), [ASP3-3/YLR158C](http://www.yeastgenome.org/cgi-bin/locus.fpl?locus=ASP3), [ASP3-4/YLR160C](http://www.yeastgenome.org/cgi-bin/locus.fpl?locus=ASP3) |
| [*cellular response to nitrogen starvation*](http://www.yeastgenome.org/cgi-bin/GO/goTerm.pl?goid=6995) | 4 out of 79 genes, 5.1% | 6 out of 7167 genes, 0.1% | 4.90e-05 | [ASP3-1/YLR155C](http://www.yeastgenome.org/cgi-bin/locus.fpl?locus=ASP3), [ASP3-2/YLR157C](http://www.yeastgenome.org/cgi-bin/locus.fpl?locus=ASP3), [ASP3-3/YLR158C](http://www.yeastgenome.org/cgi-bin/locus.fpl?locus=ASP3), [ASP3-4/YLR160C](http://www.yeastgenome.org/cgi-bin/locus.fpl?locus=ASP3) |
| [*carboxylic acid transport*](http://www.yeastgenome.org/cgi-bin/GO/goTerm.pl?goid=46942) | 8 out of 79 genes, 10.1% | 62 out of 7167 genes, 0.9% | 7.72e-05 | [AGP1/YCL025C](http://www.yeastgenome.org/cgi-bin/locus.fpl?locus=AGP1), [CAN1/YEL063C](http://www.yeastgenome.org/cgi-bin/locus.fpl?locus=CAN1), [VHT1/YGR065C](http://www.yeastgenome.org/cgi-bin/locus.fpl?locus=VHT1), [DAL5/YJR152W](http://www.yeastgenome.org/cgi-bin/locus.fpl?locus=DAL5), [GAP1/YKR039W](http://www.yeastgenome.org/cgi-bin/locus.fpl?locus=GAP1), [VBA1/YMR088C](http://www.yeastgenome.org/cgi-bin/locus.fpl?locus=VBA1), [PUT4/YOR348C](http://www.yeastgenome.org/cgi-bin/locus.fpl?locus=PUT4), [DIP5/YPL265W](http://www.yeastgenome.org/cgi-bin/locus.fpl?locus=DIP5) |
| [*organic acid transport*](http://www.yeastgenome.org/cgi-bin/GO/goTerm.pl?goid=15849) | 8 out of 79 genes, 10.1% | 63 out of 7167 genes, 0.9% | 8.77e-05 | [AGP1/YCL025C](http://www.yeastgenome.org/cgi-bin/locus.fpl?locus=AGP1), [CAN1/YEL063C](http://www.yeastgenome.org/cgi-bin/locus.fpl?locus=CAN1), [VHT1/YGR065C](http://www.yeastgenome.org/cgi-bin/locus.fpl?locus=VHT1), [DAL5/YJR152W](http://www.yeastgenome.org/cgi-bin/locus.fpl?locus=DAL5), [GAP1/YKR039W](http://www.yeastgenome.org/cgi-bin/locus.fpl?locus=GAP1), [VBA1/YMR088C](http://www.yeastgenome.org/cgi-bin/locus.fpl?locus=VBA1), [PUT4/YOR348C](http://www.yeastgenome.org/cgi-bin/locus.fpl?locus=PUT4), [DIP5/YPL265W](http://www.yeastgenome.org/cgi-bin/locus.fpl?locus=DIP5) |
| [*allantoin metabolic process*](http://www.yeastgenome.org/cgi-bin/GO/goTerm.pl?goid=255) | 4 out of 79 genes, 5.1% | 7 out of 7167 genes, 0.1% | 0.00011 | [DUR1,2/YBR208C](http://www.yeastgenome.org/cgi-bin/locus.fpl?locus=DUR1), [DAL2/YIR029W](http://www.yeastgenome.org/cgi-bin/locus.fpl?locus=DAL2), [DAL7/YIR031C](http://www.yeastgenome.org/cgi-bin/locus.fpl?locus=DAL7), [DAL3/YIR032C](http://www.yeastgenome.org/cgi-bin/locus.fpl?locus=DAL3) |
| [*allantoin catabolic process*](http://www.yeastgenome.org/cgi-bin/GO/goTerm.pl?goid=256) | 4 out of 79 genes, 5.1% | 7 out of 7167 genes, 0.1% | 0.00011 | [DUR1,2/YBR208C](http://www.yeastgenome.org/cgi-bin/locus.fpl?locus=DUR1), [DAL2/YIR029W](http://www.yeastgenome.org/cgi-bin/locus.fpl?locus=DAL2), [DAL7/YIR031C](http://www.yeastgenome.org/cgi-bin/locus.fpl?locus=DAL7), [DAL3/YIR032C](http://www.yeastgenome.org/cgi-bin/locus.fpl?locus=DAL3) |
| [*glutamine metabolic process*](http://www.yeastgenome.org/cgi-bin/GO/goTerm.pl?goid=6541) | 4 out of 79 genes, 5.1% | 7 out of 7167 genes, 0.1% | 0.00011 | [GDH3/YAL062W](http://www.yeastgenome.org/cgi-bin/locus.fpl?locus=GDH3), [GAP1/YKR039W](http://www.yeastgenome.org/cgi-bin/locus.fpl?locus=GAP1), [PUT4/YOR348C](http://www.yeastgenome.org/cgi-bin/locus.fpl?locus=PUT4), [GDH1/YOR375C](http://www.yeastgenome.org/cgi-bin/locus.fpl?locus=GDH1) |
| [*heterocycle catabolic process*](http://www.yeastgenome.org/cgi-bin/GO/goTerm.pl?goid=46700) | 7 out of 79 genes, 8.9% | 45 out of 7167 genes, 0.6% | 0.00012 | [DUR1,2/YBR208C](http://www.yeastgenome.org/cgi-bin/locus.fpl?locus=DUR1), [DAL2/YIR029W](http://www.yeastgenome.org/cgi-bin/locus.fpl?locus=DAL2), [DAL7/YIR031C](http://www.yeastgenome.org/cgi-bin/locus.fpl?locus=DAL7), [DAL3/YIR032C](http://www.yeastgenome.org/cgi-bin/locus.fpl?locus=DAL3), [PUT1/YLR142W](http://www.yeastgenome.org/cgi-bin/locus.fpl?locus=PUT1), [HMX1/YLR205C](http://www.yeastgenome.org/cgi-bin/locus.fpl?locus=HMX1), [PUT4/YOR348C](http://www.yeastgenome.org/cgi-bin/locus.fpl?locus=PUT4) |
