# Supplementary material for: A Plant Virus Movement Protein Regulates the Gcn2p Kinase in Budding Yeast
Source: PLoS One. 2011 Nov 8;6(11):e27409. doi: 10.1371/journal.pone.0027409 (PMC3210792; doi:10.1371/journal.pone.0027409)
Supplement: Table S2 — Functional classification of induced genes in MPpnrsv versus MPpnrsvΔHR expressing yeast strains. (DOC) [file pone.0027409.s003.doc]

**TABLE S2.** Functional classification of induced genes in MPpnrsv versus MPpnrsvΔHR expressing yeast strains.

| ***Gene Ontology term*** | ***Cluster frequency*** | ***Genome frequency of use*** | ***Corrected***  ***P-value*** | ***Genes annotated to the term*** |
| --- | --- | --- | --- | --- |
| [*ribosome biogenesis*](http://www.yeastgenome.org/cgi-bin/GO/goTerm.pl?goid=42254) | 79 out of 292 genes, 27.1% | 409 out of 7167 genes, 5.7% | 3.93e-31 | [UTP20/YBL004W](http://www.yeastgenome.org/cgi-bin/locus.fpl?locus=UTP20), [MAK5/YBR142W](http://www.yeastgenome.org/cgi-bin/locus.fpl?locus=MAK5), [ENP1/YBR247C](http://www.yeastgenome.org/cgi-bin/locus.fpl?locus=ENP1), [SPB1/YCL054W](http://www.yeastgenome.org/cgi-bin/locus.fpl?locus=SPB1), [TSR1/YDL060W](http://www.yeastgenome.org/cgi-bin/locus.fpl?locus=TSR1), [YDL063C](http://www.yeastgenome.org/cgi-bin/locus.fpl?locus=YDL063C), [NOP14/YDL148C](http://www.yeastgenome.org/cgi-bin/locus.fpl?locus=NOP14), [SAS10/YDL153C](http://www.yeastgenome.org/cgi-bin/locus.fpl?locus=SAS10), [NOP6/YDL213C](http://www.yeastgenome.org/cgi-bin/locus.fpl?locus=NOP6), [MAK21/YDR060W](http://www.yeastgenome.org/cgi-bin/locus.fpl?locus=MAK21), [RRP1/YDR087C](http://www.yeastgenome.org/cgi-bin/locus.fpl?locus=RRP1), [ARX1/YDR101C](http://www.yeastgenome.org/cgi-bin/locus.fpl?locus=ARX1), [SSF2/YDR312W](http://www.yeastgenome.org/cgi-bin/locus.fpl?locus=SSF2), [UTP4/YDR324C](http://www.yeastgenome.org/cgi-bin/locus.fpl?locus=UTP4), [UTP5/YDR398W](http://www.yeastgenome.org/cgi-bin/locus.fpl?locus=UTP5), [RRP17/YDR412W](http://www.yeastgenome.org/cgi-bin/locus.fpl?locus=RRP17), [UTP6/YDR449C](http://www.yeastgenome.org/cgi-bin/locus.fpl?locus=UTP6), [PUF6/YDR496C](http://www.yeastgenome.org/cgi-bin/locus.fpl?locus=PUF6), [SNU13/YEL026W](http://www.yeastgenome.org/cgi-bin/locus.fpl?locus=SNU13), [NUG1/YER006W](http://www.yeastgenome.org/cgi-bin/locus.fpl?locus=NUG1), [ARB1/YER036C](http://www.yeastgenome.org/cgi-bin/locus.fpl?locus=ARB1), [UTP7/YER082C](http://www.yeastgenome.org/cgi-bin/locus.fpl?locus=UTP7), [DBP3/YGL078C](http://www.yeastgenome.org/cgi-bin/locus.fpl?locus=DBP3), [LSG1/YGL099W](http://www.yeastgenome.org/cgi-bin/locus.fpl?locus=LSG1), [PRP43/YGL120C](http://www.yeastgenome.org/cgi-bin/locus.fpl?locus=PRP43), [NOP7/YGR103W](http://www.yeastgenome.org/cgi-bin/locus.fpl?locus=NOP7), [ENP2/YGR145W](http://www.yeastgenome.org/cgi-bin/locus.fpl?locus=ENP2), [NSR1/YGR159C](http://www.yeastgenome.org/cgi-bin/locus.fpl?locus=NSR1), [TIF4631/YGR162W](http://www.yeastgenome.org/cgi-bin/locus.fpl?locus=TIF4631), [SDA1/YGR245C](http://www.yeastgenome.org/cgi-bin/locus.fpl?locus=SDA1), [CIC1/YHR052W](http://www.yeastgenome.org/cgi-bin/locus.fpl?locus=CIC1), [GAR1/YHR089C](http://www.yeastgenome.org/cgi-bin/locus.fpl?locus=GAR1), [NMD3/YHR170W](http://www.yeastgenome.org/cgi-bin/locus.fpl?locus=NMD3), [RIX1/YHR197W](http://www.yeastgenome.org/cgi-bin/locus.fpl?locus=RIX1), [SQT1/YIR012W](http://www.yeastgenome.org/cgi-bin/locus.fpl?locus=SQT1), [MTR4/YJL050W](http://www.yeastgenome.org/cgi-bin/locus.fpl?locus=MTR4), [UTP18/YJL069C](http://www.yeastgenome.org/cgi-bin/locus.fpl?locus=UTP18), [ALB1/YJL122W](http://www.yeastgenome.org/cgi-bin/locus.fpl?locus=ALB1), [MPP10/YJR002W](http://www.yeastgenome.org/cgi-bin/locus.fpl?locus=MPP10), [MAK11/YKL021C](http://www.yeastgenome.org/cgi-bin/locus.fpl?locus=MAK11), [LTV1/YKL143W](http://www.yeastgenome.org/cgi-bin/locus.fpl?locus=LTV1), [EBP2/YKL172W](http://www.yeastgenome.org/cgi-bin/locus.fpl?locus=EBP2), [RPF2/YKR081C](http://www.yeastgenome.org/cgi-bin/locus.fpl?locus=RPF2), [RIX7/YLL034C](http://www.yeastgenome.org/cgi-bin/locus.fpl?locus=RIX7), [RLP24/YLR009W](http://www.yeastgenome.org/cgi-bin/locus.fpl?locus=RLP24), [MDN1/YLR106C](http://www.yeastgenome.org/cgi-bin/locus.fpl?locus=MDN1), [CBF5/YLR175W](http://www.yeastgenome.org/cgi-bin/locus.fpl?locus=CBF5), [PWP1/YLR196W](http://www.yeastgenome.org/cgi-bin/locus.fpl?locus=PWP1), [NOP56/YLR197W](http://www.yeastgenome.org/cgi-bin/locus.fpl?locus=NOP56), [UTP13/YLR222C](http://www.yeastgenome.org/cgi-bin/locus.fpl?locus=UTP13), [DBP9/YLR276C](http://www.yeastgenome.org/cgi-bin/locus.fpl?locus=DBP9), [GSP1/YLR293C](http://www.yeastgenome.org/cgi-bin/locus.fpl?locus=GSP1), [SGD1/YLR336C](http://www.yeastgenome.org/cgi-bin/locus.fpl?locus=SGD1), [ERB1/YMR049C](http://www.yeastgenome.org/cgi-bin/locus.fpl?locus=ERB1), [UTP15/YMR093W](http://www.yeastgenome.org/cgi-bin/locus.fpl?locus=UTP15), [ECM16/YMR128W](http://www.yeastgenome.org/cgi-bin/locus.fpl?locus=ECM16), [RRB1/YMR131C](http://www.yeastgenome.org/cgi-bin/locus.fpl?locus=RRB1), [NOP2/YNL061W](http://www.yeastgenome.org/cgi-bin/locus.fpl?locus=NOP2), [NOP15/YNL110C](http://www.yeastgenome.org/cgi-bin/locus.fpl?locus=NOP15), [DBP2/YNL112W](http://www.yeastgenome.org/cgi-bin/locus.fpl?locus=DBP2), [KRE33/YNL132W](http://www.yeastgenome.org/cgi-bin/locus.fpl?locus=KRE33), [IPI3/YNL182C](http://www.yeastgenome.org/cgi-bin/locus.fpl?locus=IPI3), [KRI1/YNL308C](http://www.yeastgenome.org/cgi-bin/locus.fpl?locus=KRI1), [NOG2/YNR053C](http://www.yeastgenome.org/cgi-bin/locus.fpl?locus=NOG2), [BRX1/YOL077C](http://www.yeastgenome.org/cgi-bin/locus.fpl?locus=BRX1), [RRP6/YOR001W](http://www.yeastgenome.org/cgi-bin/locus.fpl?locus=RRP6), [PNO1/YOR145C](http://www.yeastgenome.org/cgi-bin/locus.fpl?locus=PNO1), [NOC2/YOR206W](http://www.yeastgenome.org/cgi-bin/locus.fpl?locus=NOC2), [YTM1/YOR272W](http://www.yeastgenome.org/cgi-bin/locus.fpl?locus=YTM1), [RRS1/YOR294W](http://www.yeastgenome.org/cgi-bin/locus.fpl?locus=RRS1), [NOP58/YOR310C](http://www.yeastgenome.org/cgi-bin/locus.fpl?locus=NOP58), [RRP12/YPL012W](http://www.yeastgenome.org/cgi-bin/locus.fpl?locus=RRP12), [NOP4/YPL043W](http://www.yeastgenome.org/cgi-bin/locus.fpl?locus=NOP4), [NOG1/YPL093W](http://www.yeastgenome.org/cgi-bin/locus.fpl?locus=NOG1), [NAN1/YPL126W](http://www.yeastgenome.org/cgi-bin/locus.fpl?locus=NAN1), [NOP53/YPL146C](http://www.yeastgenome.org/cgi-bin/locus.fpl?locus=NOP53), [NIP7/YPL211W](http://www.yeastgenome.org/cgi-bin/locus.fpl?locus=NIP7), [BMS1/YPL217C](http://www.yeastgenome.org/cgi-bin/locus.fpl?locus=BMS1), [NEW1/YPL226W](http://www.yeastgenome.org/cgi-bin/locus.fpl?locus=NEW1) |
| [*ribonucleoprotein complex biogenesis*](http://www.yeastgenome.org/cgi-bin/GO/goTerm.pl?goid=22613) | 80 out of 292 genes, 27.4% | 467 out of 7167 genes, 6.5% | 1.02e-27 | [UTP20/YBL004W](http://www.yeastgenome.org/cgi-bin/locus.fpl?locus=UTP20), [MAK5/YBR142W](http://www.yeastgenome.org/cgi-bin/locus.fpl?locus=MAK5), [ENP1/YBR247C](http://www.yeastgenome.org/cgi-bin/locus.fpl?locus=ENP1), [SPB1/YCL054W](http://www.yeastgenome.org/cgi-bin/locus.fpl?locus=SPB1), [TSR1/YDL060W](http://www.yeastgenome.org/cgi-bin/locus.fpl?locus=TSR1), [YDL063C](http://www.yeastgenome.org/cgi-bin/locus.fpl?locus=YDL063C), [NOP14/YDL148C](http://www.yeastgenome.org/cgi-bin/locus.fpl?locus=NOP14), [SAS10/YDL153C](http://www.yeastgenome.org/cgi-bin/locus.fpl?locus=SAS10), [NOP6/YDL213C](http://www.yeastgenome.org/cgi-bin/locus.fpl?locus=NOP6), [MAK21/YDR060W](http://www.yeastgenome.org/cgi-bin/locus.fpl?locus=MAK21), [RRP1/YDR087C](http://www.yeastgenome.org/cgi-bin/locus.fpl?locus=RRP1), [ARX1/YDR101C](http://www.yeastgenome.org/cgi-bin/locus.fpl?locus=ARX1), [SSF2/YDR312W](http://www.yeastgenome.org/cgi-bin/locus.fpl?locus=SSF2), [UTP4/YDR324C](http://www.yeastgenome.org/cgi-bin/locus.fpl?locus=UTP4), [UTP5/YDR398W](http://www.yeastgenome.org/cgi-bin/locus.fpl?locus=UTP5), [RRP17/YDR412W](http://www.yeastgenome.org/cgi-bin/locus.fpl?locus=RRP17), [UTP6/YDR449C](http://www.yeastgenome.org/cgi-bin/locus.fpl?locus=UTP6), [PUF6/YDR496C](http://www.yeastgenome.org/cgi-bin/locus.fpl?locus=PUF6), [SNU13/YEL026W](http://www.yeastgenome.org/cgi-bin/locus.fpl?locus=SNU13), [NUG1/YER006W](http://www.yeastgenome.org/cgi-bin/locus.fpl?locus=NUG1), [ARB1/YER036C](http://www.yeastgenome.org/cgi-bin/locus.fpl?locus=ARB1), [UTP7/YER082C](http://www.yeastgenome.org/cgi-bin/locus.fpl?locus=UTP7), [BUD27/YFL023W](http://www.yeastgenome.org/cgi-bin/locus.fpl?locus=BUD27), [DBP3/YGL078C](http://www.yeastgenome.org/cgi-bin/locus.fpl?locus=DBP3), [LSG1/YGL099W](http://www.yeastgenome.org/cgi-bin/locus.fpl?locus=LSG1), [PRP43/YGL120C](http://www.yeastgenome.org/cgi-bin/locus.fpl?locus=PRP43), [NOP7/YGR103W](http://www.yeastgenome.org/cgi-bin/locus.fpl?locus=NOP7), [ENP2/YGR145W](http://www.yeastgenome.org/cgi-bin/locus.fpl?locus=ENP2), [NSR1/YGR159C](http://www.yeastgenome.org/cgi-bin/locus.fpl?locus=NSR1), [TIF4631/YGR162W](http://www.yeastgenome.org/cgi-bin/locus.fpl?locus=TIF4631), [SDA1/YGR245C](http://www.yeastgenome.org/cgi-bin/locus.fpl?locus=SDA1), [CIC1/YHR052W](http://www.yeastgenome.org/cgi-bin/locus.fpl?locus=CIC1), [GAR1/YHR089C](http://www.yeastgenome.org/cgi-bin/locus.fpl?locus=GAR1), [NMD3/YHR170W](http://www.yeastgenome.org/cgi-bin/locus.fpl?locus=NMD3), [RIX1/YHR197W](http://www.yeastgenome.org/cgi-bin/locus.fpl?locus=RIX1), [SQT1/YIR012W](http://www.yeastgenome.org/cgi-bin/locus.fpl?locus=SQT1), [MTR4/YJL050W](http://www.yeastgenome.org/cgi-bin/locus.fpl?locus=MTR4), [UTP18/YJL069C](http://www.yeastgenome.org/cgi-bin/locus.fpl?locus=UTP18), [ALB1/YJL122W](http://www.yeastgenome.org/cgi-bin/locus.fpl?locus=ALB1), [MPP10/YJR002W](http://www.yeastgenome.org/cgi-bin/locus.fpl?locus=MPP10), [MAK11/YKL021C](http://www.yeastgenome.org/cgi-bin/locus.fpl?locus=MAK11), [LTV1/YKL143W](http://www.yeastgenome.org/cgi-bin/locus.fpl?locus=LTV1), [EBP2/YKL172W](http://www.yeastgenome.org/cgi-bin/locus.fpl?locus=EBP2), [RPF2/YKR081C](http://www.yeastgenome.org/cgi-bin/locus.fpl?locus=RPF2), [RIX7/YLL034C](http://www.yeastgenome.org/cgi-bin/locus.fpl?locus=RIX7), [RLP24/YLR009W](http://www.yeastgenome.org/cgi-bin/locus.fpl?locus=RLP24), [MDN1/YLR106C](http://www.yeastgenome.org/cgi-bin/locus.fpl?locus=MDN1), [CBF5/YLR175W](http://www.yeastgenome.org/cgi-bin/locus.fpl?locus=CBF5), [PWP1/YLR196W](http://www.yeastgenome.org/cgi-bin/locus.fpl?locus=PWP1), [NOP56/YLR197W](http://www.yeastgenome.org/cgi-bin/locus.fpl?locus=NOP56), [UTP13/YLR222C](http://www.yeastgenome.org/cgi-bin/locus.fpl?locus=UTP13), [DBP9/YLR276C](http://www.yeastgenome.org/cgi-bin/locus.fpl?locus=DBP9), [GSP1/YLR293C](http://www.yeastgenome.org/cgi-bin/locus.fpl?locus=GSP1), [SGD1/YLR336C](http://www.yeastgenome.org/cgi-bin/locus.fpl?locus=SGD1), [ERB1/YMR049C](http://www.yeastgenome.org/cgi-bin/locus.fpl?locus=ERB1), [UTP15/YMR093W](http://www.yeastgenome.org/cgi-bin/locus.fpl?locus=UTP15), [ECM16/YMR128W](http://www.yeastgenome.org/cgi-bin/locus.fpl?locus=ECM16), [RRB1/YMR131C](http://www.yeastgenome.org/cgi-bin/locus.fpl?locus=RRB1), [NOP2/YNL061W](http://www.yeastgenome.org/cgi-bin/locus.fpl?locus=NOP2), [NOP15/YNL110C](http://www.yeastgenome.org/cgi-bin/locus.fpl?locus=NOP15), [DBP2/YNL112W](http://www.yeastgenome.org/cgi-bin/locus.fpl?locus=DBP2), [KRE33/YNL132W](http://www.yeastgenome.org/cgi-bin/locus.fpl?locus=KRE33), [IPI3/YNL182C](http://www.yeastgenome.org/cgi-bin/locus.fpl?locus=IPI3), [KRI1/YNL308C](http://www.yeastgenome.org/cgi-bin/locus.fpl?locus=KRI1), [NOG2/YNR053C](http://www.yeastgenome.org/cgi-bin/locus.fpl?locus=NOG2), [BRX1/YOL077C](http://www.yeastgenome.org/cgi-bin/locus.fpl?locus=BRX1), [RRP6/YOR001W](http://www.yeastgenome.org/cgi-bin/locus.fpl?locus=RRP6), [PNO1/YOR145C](http://www.yeastgenome.org/cgi-bin/locus.fpl?locus=PNO1), [NOC2/YOR206W](http://www.yeastgenome.org/cgi-bin/locus.fpl?locus=NOC2), [YTM1/YOR272W](http://www.yeastgenome.org/cgi-bin/locus.fpl?locus=YTM1), [RRS1/YOR294W](http://www.yeastgenome.org/cgi-bin/locus.fpl?locus=RRS1), [NOP58/YOR310C](http://www.yeastgenome.org/cgi-bin/locus.fpl?locus=NOP58), [RRP12/YPL012W](http://www.yeastgenome.org/cgi-bin/locus.fpl?locus=RRP12), [NOP4/YPL043W](http://www.yeastgenome.org/cgi-bin/locus.fpl?locus=NOP4), [NOG1/YPL093W](http://www.yeastgenome.org/cgi-bin/locus.fpl?locus=NOG1), [NAN1/YPL126W](http://www.yeastgenome.org/cgi-bin/locus.fpl?locus=NAN1), [NOP53/YPL146C](http://www.yeastgenome.org/cgi-bin/locus.fpl?locus=NOP53), [NIP7/YPL211W](http://www.yeastgenome.org/cgi-bin/locus.fpl?locus=NIP7), [BMS1/YPL217C](http://www.yeastgenome.org/cgi-bin/locus.fpl?locus=BMS1), [NEW1/YPL226W](http://www.yeastgenome.org/cgi-bin/locus.fpl?locus=NEW1) |
| [*ribosomal large subunit biogenesis*](http://www.yeastgenome.org/cgi-bin/GO/goTerm.pl?goid=42273) | 30 out of 292 genes, 10.3% | 72 out of 7167 genes, 1.0% | 1.07e-20 | [MAK5/YBR142W](http://www.yeastgenome.org/cgi-bin/locus.fpl?locus=MAK5), [YDL063C](http://www.yeastgenome.org/cgi-bin/locus.fpl?locus=YDL063C), [MAK21/YDR060W](http://www.yeastgenome.org/cgi-bin/locus.fpl?locus=MAK21), [ARX1/YDR101C](http://www.yeastgenome.org/cgi-bin/locus.fpl?locus=ARX1), [SSF2/YDR312W](http://www.yeastgenome.org/cgi-bin/locus.fpl?locus=SSF2), [PUF6/YDR496C](http://www.yeastgenome.org/cgi-bin/locus.fpl?locus=PUF6), [DBP3/YGL078C](http://www.yeastgenome.org/cgi-bin/locus.fpl?locus=DBP3), [PRP43/YGL120C](http://www.yeastgenome.org/cgi-bin/locus.fpl?locus=PRP43), [NOP7/YGR103W](http://www.yeastgenome.org/cgi-bin/locus.fpl?locus=NOP7), [TIF4631/YGR162W](http://www.yeastgenome.org/cgi-bin/locus.fpl?locus=TIF4631), [SDA1/YGR245C](http://www.yeastgenome.org/cgi-bin/locus.fpl?locus=SDA1), [CIC1/YHR052W](http://www.yeastgenome.org/cgi-bin/locus.fpl?locus=CIC1), [NMD3/YHR170W](http://www.yeastgenome.org/cgi-bin/locus.fpl?locus=NMD3), [RIX1/YHR197W](http://www.yeastgenome.org/cgi-bin/locus.fpl?locus=RIX1), [SQT1/YIR012W](http://www.yeastgenome.org/cgi-bin/locus.fpl?locus=SQT1), [ALB1/YJL122W](http://www.yeastgenome.org/cgi-bin/locus.fpl?locus=ALB1), [MAK11/YKL021C](http://www.yeastgenome.org/cgi-bin/locus.fpl?locus=MAK11), [RPF2/YKR081C](http://www.yeastgenome.org/cgi-bin/locus.fpl?locus=RPF2), [RIX7/YLL034C](http://www.yeastgenome.org/cgi-bin/locus.fpl?locus=RIX7), [RLP24/YLR009W](http://www.yeastgenome.org/cgi-bin/locus.fpl?locus=RLP24), [MDN1/YLR106C](http://www.yeastgenome.org/cgi-bin/locus.fpl?locus=MDN1), [DBP9/YLR276C](http://www.yeastgenome.org/cgi-bin/locus.fpl?locus=DBP9), [ERB1/YMR049C](http://www.yeastgenome.org/cgi-bin/locus.fpl?locus=ERB1), [NOP15/YNL110C](http://www.yeastgenome.org/cgi-bin/locus.fpl?locus=NOP15), [IPI3/YNL182C](http://www.yeastgenome.org/cgi-bin/locus.fpl?locus=IPI3), [BRX1/YOL077C](http://www.yeastgenome.org/cgi-bin/locus.fpl?locus=BRX1), [YTM1/YOR272W](http://www.yeastgenome.org/cgi-bin/locus.fpl?locus=YTM1), [RRS1/YOR294W](http://www.yeastgenome.org/cgi-bin/locus.fpl?locus=RRS1), [NOG1/YPL093W](http://www.yeastgenome.org/cgi-bin/locus.fpl?locus=NOG1), [NIP7/YPL211W](http://www.yeastgenome.org/cgi-bin/locus.fpl?locus=NIP7) |
| [*ncRNA processing*](http://www.yeastgenome.org/cgi-bin/GO/goTerm.pl?goid=34470) | 62 out of 292 genes, 21.2% | 386 out of 7167 genes, 5.4% | 4.87e-19 | [UTP20/YBL004W](http://www.yeastgenome.org/cgi-bin/locus.fpl?locus=UTP20), [NCL1/YBL024W](http://www.yeastgenome.org/cgi-bin/locus.fpl?locus=NCL1), [MAK5/YBR142W](http://www.yeastgenome.org/cgi-bin/locus.fpl?locus=MAK5), [ENP1/YBR247C](http://www.yeastgenome.org/cgi-bin/locus.fpl?locus=ENP1), [SPB1/YCL054W](http://www.yeastgenome.org/cgi-bin/locus.fpl?locus=SPB1), [TSR1/YDL060W](http://www.yeastgenome.org/cgi-bin/locus.fpl?locus=TSR1), [NOP14/YDL148C](http://www.yeastgenome.org/cgi-bin/locus.fpl?locus=NOP14), [SAS10/YDL153C](http://www.yeastgenome.org/cgi-bin/locus.fpl?locus=SAS10), [TRM8/YDL201W](http://www.yeastgenome.org/cgi-bin/locus.fpl?locus=TRM8), [RRP1/YDR087C](http://www.yeastgenome.org/cgi-bin/locus.fpl?locus=RRP1), [UTP4/YDR324C](http://www.yeastgenome.org/cgi-bin/locus.fpl?locus=UTP4), [UTP5/YDR398W](http://www.yeastgenome.org/cgi-bin/locus.fpl?locus=UTP5), [RRP17/YDR412W](http://www.yeastgenome.org/cgi-bin/locus.fpl?locus=RRP17), [UTP6/YDR449C](http://www.yeastgenome.org/cgi-bin/locus.fpl?locus=UTP6), [SNU13/YEL026W](http://www.yeastgenome.org/cgi-bin/locus.fpl?locus=SNU13), [NUG1/YER006W](http://www.yeastgenome.org/cgi-bin/locus.fpl?locus=NUG1), [UTP7/YER082C](http://www.yeastgenome.org/cgi-bin/locus.fpl?locus=UTP7), [DBP3/YGL078C](http://www.yeastgenome.org/cgi-bin/locus.fpl?locus=DBP3), [PRP43/YGL120C](http://www.yeastgenome.org/cgi-bin/locus.fpl?locus=PRP43), [NOP7/YGR103W](http://www.yeastgenome.org/cgi-bin/locus.fpl?locus=NOP7), [ENP2/YGR145W](http://www.yeastgenome.org/cgi-bin/locus.fpl?locus=ENP2), [NSR1/YGR159C](http://www.yeastgenome.org/cgi-bin/locus.fpl?locus=NSR1), [PXR1/YGR280C](http://www.yeastgenome.org/cgi-bin/locus.fpl?locus=PXR1), [GAR1/YHR089C](http://www.yeastgenome.org/cgi-bin/locus.fpl?locus=GAR1), [RIX1/YHR197W](http://www.yeastgenome.org/cgi-bin/locus.fpl?locus=RIX1), [MTR4/YJL050W](http://www.yeastgenome.org/cgi-bin/locus.fpl?locus=MTR4), [UTP18/YJL069C](http://www.yeastgenome.org/cgi-bin/locus.fpl?locus=UTP18), [MPP10/YJR002W](http://www.yeastgenome.org/cgi-bin/locus.fpl?locus=MPP10), [MAK11/YKL021C](http://www.yeastgenome.org/cgi-bin/locus.fpl?locus=MAK11), [EBP2/YKL172W](http://www.yeastgenome.org/cgi-bin/locus.fpl?locus=EBP2), [RPF2/YKR081C](http://www.yeastgenome.org/cgi-bin/locus.fpl?locus=RPF2), [MDN1/YLR106C](http://www.yeastgenome.org/cgi-bin/locus.fpl?locus=MDN1), [CBF5/YLR175W](http://www.yeastgenome.org/cgi-bin/locus.fpl?locus=CBF5), [PWP1/YLR196W](http://www.yeastgenome.org/cgi-bin/locus.fpl?locus=PWP1), [NOP56/YLR197W](http://www.yeastgenome.org/cgi-bin/locus.fpl?locus=NOP56), [UTP13/YLR222C](http://www.yeastgenome.org/cgi-bin/locus.fpl?locus=UTP13), [DBP9/YLR276C](http://www.yeastgenome.org/cgi-bin/locus.fpl?locus=DBP9), [GSP1/YLR293C](http://www.yeastgenome.org/cgi-bin/locus.fpl?locus=GSP1), [DUS3/YLR401C](http://www.yeastgenome.org/cgi-bin/locus.fpl?locus=DUS3), [ERB1/YMR049C](http://www.yeastgenome.org/cgi-bin/locus.fpl?locus=ERB1), [UTP15/YMR093W](http://www.yeastgenome.org/cgi-bin/locus.fpl?locus=UTP15), [ECM16/YMR128W](http://www.yeastgenome.org/cgi-bin/locus.fpl?locus=ECM16), [NOP2/YNL061W](http://www.yeastgenome.org/cgi-bin/locus.fpl?locus=NOP2), [DBP2/YNL112W](http://www.yeastgenome.org/cgi-bin/locus.fpl?locus=DBP2), [IPI3/YNL182C](http://www.yeastgenome.org/cgi-bin/locus.fpl?locus=IPI3), [PUS4/YNL292W](http://www.yeastgenome.org/cgi-bin/locus.fpl?locus=PUS4), [KRI1/YNL308C](http://www.yeastgenome.org/cgi-bin/locus.fpl?locus=KRI1), [TRM112/YNR046W](http://www.yeastgenome.org/cgi-bin/locus.fpl?locus=TRM112), [TRM11/YOL124C](http://www.yeastgenome.org/cgi-bin/locus.fpl?locus=TRM11), [RRP6/YOR001W](http://www.yeastgenome.org/cgi-bin/locus.fpl?locus=RRP6), [PNO1/YOR145C](http://www.yeastgenome.org/cgi-bin/locus.fpl?locus=PNO1), [RRS1/YOR294W](http://www.yeastgenome.org/cgi-bin/locus.fpl?locus=RRS1), [NOP58/YOR310C](http://www.yeastgenome.org/cgi-bin/locus.fpl?locus=NOP58), [RRP12/YPL012W](http://www.yeastgenome.org/cgi-bin/locus.fpl?locus=RRP12), [NOP4/YPL043W](http://www.yeastgenome.org/cgi-bin/locus.fpl?locus=NOP4), [ELP3/YPL086C](http://www.yeastgenome.org/cgi-bin/locus.fpl?locus=ELP3), [NOG1/YPL093W](http://www.yeastgenome.org/cgi-bin/locus.fpl?locus=NOG1), [NAN1/YPL126W](http://www.yeastgenome.org/cgi-bin/locus.fpl?locus=NAN1), [NOP53/YPL146C](http://www.yeastgenome.org/cgi-bin/locus.fpl?locus=NOP53), [TYW1/YPL207W](http://www.yeastgenome.org/cgi-bin/locus.fpl?locus=TYW1), [NIP7/YPL211W](http://www.yeastgenome.org/cgi-bin/locus.fpl?locus=NIP7), [BMS1/YPL217C](http://www.yeastgenome.org/cgi-bin/locus.fpl?locus=BMS1) |
| [*rRNA processing*](http://www.yeastgenome.org/cgi-bin/GO/goTerm.pl?goid=6364) | 53 out of 292 genes, 18.2% | 286 out of 7167 genes, 4.0% | 8.49e-19 | [UTP20/YBL004W](http://www.yeastgenome.org/cgi-bin/locus.fpl?locus=UTP20), [MAK5/YBR142W](http://www.yeastgenome.org/cgi-bin/locus.fpl?locus=MAK5), [ENP1/YBR247C](http://www.yeastgenome.org/cgi-bin/locus.fpl?locus=ENP1), [SPB1/YCL054W](http://www.yeastgenome.org/cgi-bin/locus.fpl?locus=SPB1), [TSR1/YDL060W](http://www.yeastgenome.org/cgi-bin/locus.fpl?locus=TSR1), [NOP14/YDL148C](http://www.yeastgenome.org/cgi-bin/locus.fpl?locus=NOP14), [SAS10/YDL153C](http://www.yeastgenome.org/cgi-bin/locus.fpl?locus=SAS10), [RRP1/YDR087C](http://www.yeastgenome.org/cgi-bin/locus.fpl?locus=RRP1), [UTP4/YDR324C](http://www.yeastgenome.org/cgi-bin/locus.fpl?locus=UTP4), [UTP5/YDR398W](http://www.yeastgenome.org/cgi-bin/locus.fpl?locus=UTP5), [RRP17/YDR412W](http://www.yeastgenome.org/cgi-bin/locus.fpl?locus=RRP17), [UTP6/YDR449C](http://www.yeastgenome.org/cgi-bin/locus.fpl?locus=UTP6), [SNU13/YEL026W](http://www.yeastgenome.org/cgi-bin/locus.fpl?locus=SNU13), [NUG1/YER006W](http://www.yeastgenome.org/cgi-bin/locus.fpl?locus=NUG1), [UTP7/YER082C](http://www.yeastgenome.org/cgi-bin/locus.fpl?locus=UTP7), [DBP3/YGL078C](http://www.yeastgenome.org/cgi-bin/locus.fpl?locus=DBP3), [PRP43/YGL120C](http://www.yeastgenome.org/cgi-bin/locus.fpl?locus=PRP43), [NOP7/YGR103W](http://www.yeastgenome.org/cgi-bin/locus.fpl?locus=NOP7), [ENP2/YGR145W](http://www.yeastgenome.org/cgi-bin/locus.fpl?locus=ENP2), [NSR1/YGR159C](http://www.yeastgenome.org/cgi-bin/locus.fpl?locus=NSR1), [GAR1/YHR089C](http://www.yeastgenome.org/cgi-bin/locus.fpl?locus=GAR1), [RIX1/YHR197W](http://www.yeastgenome.org/cgi-bin/locus.fpl?locus=RIX1), [MTR4/YJL050W](http://www.yeastgenome.org/cgi-bin/locus.fpl?locus=MTR4), [UTP18/YJL069C](http://www.yeastgenome.org/cgi-bin/locus.fpl?locus=UTP18), [MPP10/YJR002W](http://www.yeastgenome.org/cgi-bin/locus.fpl?locus=MPP10), [MAK11/YKL021C](http://www.yeastgenome.org/cgi-bin/locus.fpl?locus=MAK11), [EBP2/YKL172W](http://www.yeastgenome.org/cgi-bin/locus.fpl?locus=EBP2), [RPF2/YKR081C](http://www.yeastgenome.org/cgi-bin/locus.fpl?locus=RPF2), [MDN1/YLR106C](http://www.yeastgenome.org/cgi-bin/locus.fpl?locus=MDN1), [CBF5/YLR175W](http://www.yeastgenome.org/cgi-bin/locus.fpl?locus=CBF5), [PWP1/YLR196W](http://www.yeastgenome.org/cgi-bin/locus.fpl?locus=PWP1), [NOP56/YLR197W](http://www.yeastgenome.org/cgi-bin/locus.fpl?locus=NOP56), [UTP13/YLR222C](http://www.yeastgenome.org/cgi-bin/locus.fpl?locus=UTP13), [DBP9/YLR276C](http://www.yeastgenome.org/cgi-bin/locus.fpl?locus=DBP9), [GSP1/YLR293C](http://www.yeastgenome.org/cgi-bin/locus.fpl?locus=GSP1), [ERB1/YMR049C](http://www.yeastgenome.org/cgi-bin/locus.fpl?locus=ERB1), [UTP15/YMR093W](http://www.yeastgenome.org/cgi-bin/locus.fpl?locus=UTP15), [ECM16/YMR128W](http://www.yeastgenome.org/cgi-bin/locus.fpl?locus=ECM16), [NOP2/YNL061W](http://www.yeastgenome.org/cgi-bin/locus.fpl?locus=NOP2), [DBP2/YNL112W](http://www.yeastgenome.org/cgi-bin/locus.fpl?locus=DBP2), [IPI3/YNL182C](http://www.yeastgenome.org/cgi-bin/locus.fpl?locus=IPI3), [KRI1/YNL308C](http://www.yeastgenome.org/cgi-bin/locus.fpl?locus=KRI1), [RRP6/YOR001W](http://www.yeastgenome.org/cgi-bin/locus.fpl?locus=RRP6), [PNO1/YOR145C](http://www.yeastgenome.org/cgi-bin/locus.fpl?locus=PNO1), [RRS1/YOR294W](http://www.yeastgenome.org/cgi-bin/locus.fpl?locus=RRS1), [NOP58/YOR310C](http://www.yeastgenome.org/cgi-bin/locus.fpl?locus=NOP58), [RRP12/YPL012W](http://www.yeastgenome.org/cgi-bin/locus.fpl?locus=RRP12), [NOP4/YPL043W](http://www.yeastgenome.org/cgi-bin/locus.fpl?locus=NOP4), [NOG1/YPL093W](http://www.yeastgenome.org/cgi-bin/locus.fpl?locus=NOG1), [NAN1/YPL126W](http://www.yeastgenome.org/cgi-bin/locus.fpl?locus=NAN1), [NOP53/YPL146C](http://www.yeastgenome.org/cgi-bin/locus.fpl?locus=NOP53), [NIP7/YPL211W](http://www.yeastgenome.org/cgi-bin/locus.fpl?locus=NIP7), [BMS1/YPL217C](http://www.yeastgenome.org/cgi-bin/locus.fpl?locus=BMS1) |
| [*rRNA metabolic process*](http://www.yeastgenome.org/cgi-bin/GO/goTerm.pl?goid=16072) | 53 out of 292 genes, 18.2% | 298 out of 7167 genes, 4.2% | 6.18e-18 | [UTP20/YBL004W](http://www.yeastgenome.org/cgi-bin/locus.fpl?locus=UTP20), [MAK5/YBR142W](http://www.yeastgenome.org/cgi-bin/locus.fpl?locus=MAK5), [ENP1/YBR247C](http://www.yeastgenome.org/cgi-bin/locus.fpl?locus=ENP1), [SPB1/YCL054W](http://www.yeastgenome.org/cgi-bin/locus.fpl?locus=SPB1), [TSR1/YDL060W](http://www.yeastgenome.org/cgi-bin/locus.fpl?locus=TSR1), [NOP14/YDL148C](http://www.yeastgenome.org/cgi-bin/locus.fpl?locus=NOP14), [SAS10/YDL153C](http://www.yeastgenome.org/cgi-bin/locus.fpl?locus=SAS10), [RRP1/YDR087C](http://www.yeastgenome.org/cgi-bin/locus.fpl?locus=RRP1), [UTP4/YDR324C](http://www.yeastgenome.org/cgi-bin/locus.fpl?locus=UTP4), [UTP5/YDR398W](http://www.yeastgenome.org/cgi-bin/locus.fpl?locus=UTP5), [RRP17/YDR412W](http://www.yeastgenome.org/cgi-bin/locus.fpl?locus=RRP17), [UTP6/YDR449C](http://www.yeastgenome.org/cgi-bin/locus.fpl?locus=UTP6), [SNU13/YEL026W](http://www.yeastgenome.org/cgi-bin/locus.fpl?locus=SNU13), [NUG1/YER006W](http://www.yeastgenome.org/cgi-bin/locus.fpl?locus=NUG1), [UTP7/YER082C](http://www.yeastgenome.org/cgi-bin/locus.fpl?locus=UTP7), [DBP3/YGL078C](http://www.yeastgenome.org/cgi-bin/locus.fpl?locus=DBP3), [PRP43/YGL120C](http://www.yeastgenome.org/cgi-bin/locus.fpl?locus=PRP43), [NOP7/YGR103W](http://www.yeastgenome.org/cgi-bin/locus.fpl?locus=NOP7), [ENP2/YGR145W](http://www.yeastgenome.org/cgi-bin/locus.fpl?locus=ENP2), [NSR1/YGR159C](http://www.yeastgenome.org/cgi-bin/locus.fpl?locus=NSR1), [GAR1/YHR089C](http://www.yeastgenome.org/cgi-bin/locus.fpl?locus=GAR1), [RIX1/YHR197W](http://www.yeastgenome.org/cgi-bin/locus.fpl?locus=RIX1), [MTR4/YJL050W](http://www.yeastgenome.org/cgi-bin/locus.fpl?locus=MTR4), [UTP18/YJL069C](http://www.yeastgenome.org/cgi-bin/locus.fpl?locus=UTP18), [MPP10/YJR002W](http://www.yeastgenome.org/cgi-bin/locus.fpl?locus=MPP10), [MAK11/YKL021C](http://www.yeastgenome.org/cgi-bin/locus.fpl?locus=MAK11), [EBP2/YKL172W](http://www.yeastgenome.org/cgi-bin/locus.fpl?locus=EBP2), [RPF2/YKR081C](http://www.yeastgenome.org/cgi-bin/locus.fpl?locus=RPF2), [MDN1/YLR106C](http://www.yeastgenome.org/cgi-bin/locus.fpl?locus=MDN1), [CBF5/YLR175W](http://www.yeastgenome.org/cgi-bin/locus.fpl?locus=CBF5), [PWP1/YLR196W](http://www.yeastgenome.org/cgi-bin/locus.fpl?locus=PWP1), [NOP56/YLR197W](http://www.yeastgenome.org/cgi-bin/locus.fpl?locus=NOP56), [UTP13/YLR222C](http://www.yeastgenome.org/cgi-bin/locus.fpl?locus=UTP13), [DBP9/YLR276C](http://www.yeastgenome.org/cgi-bin/locus.fpl?locus=DBP9), [GSP1/YLR293C](http://www.yeastgenome.org/cgi-bin/locus.fpl?locus=GSP1), [ERB1/YMR049C](http://www.yeastgenome.org/cgi-bin/locus.fpl?locus=ERB1), [UTP15/YMR093W](http://www.yeastgenome.org/cgi-bin/locus.fpl?locus=UTP15), [ECM16/YMR128W](http://www.yeastgenome.org/cgi-bin/locus.fpl?locus=ECM16), [NOP2/YNL061W](http://www.yeastgenome.org/cgi-bin/locus.fpl?locus=NOP2), [DBP2/YNL112W](http://www.yeastgenome.org/cgi-bin/locus.fpl?locus=DBP2), [IPI3/YNL182C](http://www.yeastgenome.org/cgi-bin/locus.fpl?locus=IPI3), [KRI1/YNL308C](http://www.yeastgenome.org/cgi-bin/locus.fpl?locus=KRI1), [RRP6/YOR001W](http://www.yeastgenome.org/cgi-bin/locus.fpl?locus=RRP6), [PNO1/YOR145C](http://www.yeastgenome.org/cgi-bin/locus.fpl?locus=PNO1), [RRS1/YOR294W](http://www.yeastgenome.org/cgi-bin/locus.fpl?locus=RRS1), [NOP58/YOR310C](http://www.yeastgenome.org/cgi-bin/locus.fpl?locus=NOP58), [RRP12/YPL012W](http://www.yeastgenome.org/cgi-bin/locus.fpl?locus=RRP12), [NOP4/YPL043W](http://www.yeastgenome.org/cgi-bin/locus.fpl?locus=NOP4), [NOG1/YPL093W](http://www.yeastgenome.org/cgi-bin/locus.fpl?locus=NOG1), [NAN1/YPL126W](http://www.yeastgenome.org/cgi-bin/locus.fpl?locus=NAN1), [NOP53/YPL146C](http://www.yeastgenome.org/cgi-bin/locus.fpl?locus=NOP53), [NIP7/YPL211W](http://www.yeastgenome.org/cgi-bin/locus.fpl?locus=NIP7), [BMS1/YPL217C](http://www.yeastgenome.org/cgi-bin/locus.fpl?locus=BMS1) |
| [*ncRNA metabolic process*](http://www.yeastgenome.org/cgi-bin/GO/goTerm.pl?goid=34660) | 64 out of 292 genes, 21.9% | 436 out of 7167 genes, 6.1% | 1.35e-17 | [UTP20/YBL004W](http://www.yeastgenome.org/cgi-bin/locus.fpl?locus=UTP20), [NCL1/YBL024W](http://www.yeastgenome.org/cgi-bin/locus.fpl?locus=NCL1), [MAK5/YBR142W](http://www.yeastgenome.org/cgi-bin/locus.fpl?locus=MAK5), [ENP1/YBR247C](http://www.yeastgenome.org/cgi-bin/locus.fpl?locus=ENP1), [SPB1/YCL054W](http://www.yeastgenome.org/cgi-bin/locus.fpl?locus=SPB1), [TSR1/YDL060W](http://www.yeastgenome.org/cgi-bin/locus.fpl?locus=TSR1), [NOP14/YDL148C](http://www.yeastgenome.org/cgi-bin/locus.fpl?locus=NOP14), [SAS10/YDL153C](http://www.yeastgenome.org/cgi-bin/locus.fpl?locus=SAS10), [TRM8/YDL201W](http://www.yeastgenome.org/cgi-bin/locus.fpl?locus=TRM8), [RRP1/YDR087C](http://www.yeastgenome.org/cgi-bin/locus.fpl?locus=RRP1), [UTP4/YDR324C](http://www.yeastgenome.org/cgi-bin/locus.fpl?locus=UTP4), [UTP5/YDR398W](http://www.yeastgenome.org/cgi-bin/locus.fpl?locus=UTP5), [RRP17/YDR412W](http://www.yeastgenome.org/cgi-bin/locus.fpl?locus=RRP17), [UTP6/YDR449C](http://www.yeastgenome.org/cgi-bin/locus.fpl?locus=UTP6), [SNU13/YEL026W](http://www.yeastgenome.org/cgi-bin/locus.fpl?locus=SNU13), [NUG1/YER006W](http://www.yeastgenome.org/cgi-bin/locus.fpl?locus=NUG1), [UTP7/YER082C](http://www.yeastgenome.org/cgi-bin/locus.fpl?locus=UTP7), [DBP3/YGL078C](http://www.yeastgenome.org/cgi-bin/locus.fpl?locus=DBP3), [PRP43/YGL120C](http://www.yeastgenome.org/cgi-bin/locus.fpl?locus=PRP43), [NOP7/YGR103W](http://www.yeastgenome.org/cgi-bin/locus.fpl?locus=NOP7), [ENP2/YGR145W](http://www.yeastgenome.org/cgi-bin/locus.fpl?locus=ENP2), [NSR1/YGR159C](http://www.yeastgenome.org/cgi-bin/locus.fpl?locus=NSR1), [PXR1/YGR280C](http://www.yeastgenome.org/cgi-bin/locus.fpl?locus=PXR1), [GAR1/YHR089C](http://www.yeastgenome.org/cgi-bin/locus.fpl?locus=GAR1), [RIX1/YHR197W](http://www.yeastgenome.org/cgi-bin/locus.fpl?locus=RIX1), [MTR4/YJL050W](http://www.yeastgenome.org/cgi-bin/locus.fpl?locus=MTR4), [UTP18/YJL069C](http://www.yeastgenome.org/cgi-bin/locus.fpl?locus=UTP18), [MPP10/YJR002W](http://www.yeastgenome.org/cgi-bin/locus.fpl?locus=MPP10), [MAK11/YKL021C](http://www.yeastgenome.org/cgi-bin/locus.fpl?locus=MAK11), [EBP2/YKL172W](http://www.yeastgenome.org/cgi-bin/locus.fpl?locus=EBP2), [RPF2/YKR081C](http://www.yeastgenome.org/cgi-bin/locus.fpl?locus=RPF2), [FRS1/YLR060W](http://www.yeastgenome.org/cgi-bin/locus.fpl?locus=FRS1), [MDN1/YLR106C](http://www.yeastgenome.org/cgi-bin/locus.fpl?locus=MDN1), [CBF5/YLR175W](http://www.yeastgenome.org/cgi-bin/locus.fpl?locus=CBF5), [PWP1/YLR196W](http://www.yeastgenome.org/cgi-bin/locus.fpl?locus=PWP1), [NOP56/YLR197W](http://www.yeastgenome.org/cgi-bin/locus.fpl?locus=NOP56), [UTP13/YLR222C](http://www.yeastgenome.org/cgi-bin/locus.fpl?locus=UTP13), [DBP9/YLR276C](http://www.yeastgenome.org/cgi-bin/locus.fpl?locus=DBP9), [GSP1/YLR293C](http://www.yeastgenome.org/cgi-bin/locus.fpl?locus=GSP1), [DUS3/YLR401C](http://www.yeastgenome.org/cgi-bin/locus.fpl?locus=DUS3), [ERB1/YMR049C](http://www.yeastgenome.org/cgi-bin/locus.fpl?locus=ERB1), [UTP15/YMR093W](http://www.yeastgenome.org/cgi-bin/locus.fpl?locus=UTP15), [ECM16/YMR128W](http://www.yeastgenome.org/cgi-bin/locus.fpl?locus=ECM16), [NOP2/YNL061W](http://www.yeastgenome.org/cgi-bin/locus.fpl?locus=NOP2), [DBP2/YNL112W](http://www.yeastgenome.org/cgi-bin/locus.fpl?locus=DBP2), [IPI3/YNL182C](http://www.yeastgenome.org/cgi-bin/locus.fpl?locus=IPI3), [PUS4/YNL292W](http://www.yeastgenome.org/cgi-bin/locus.fpl?locus=PUS4), [KRI1/YNL308C](http://www.yeastgenome.org/cgi-bin/locus.fpl?locus=KRI1), [TRM112/YNR046W](http://www.yeastgenome.org/cgi-bin/locus.fpl?locus=TRM112), [TRM11/YOL124C](http://www.yeastgenome.org/cgi-bin/locus.fpl?locus=TRM11), [RRP6/YOR001W](http://www.yeastgenome.org/cgi-bin/locus.fpl?locus=RRP6), [PNO1/YOR145C](http://www.yeastgenome.org/cgi-bin/locus.fpl?locus=PNO1), [GLN4/YOR168W](http://www.yeastgenome.org/cgi-bin/locus.fpl?locus=GLN4), [RRS1/YOR294W](http://www.yeastgenome.org/cgi-bin/locus.fpl?locus=RRS1), [NOP58/YOR310C](http://www.yeastgenome.org/cgi-bin/locus.fpl?locus=NOP58), [RRP12/YPL012W](http://www.yeastgenome.org/cgi-bin/locus.fpl?locus=RRP12), [NOP4/YPL043W](http://www.yeastgenome.org/cgi-bin/locus.fpl?locus=NOP4), [ELP3/YPL086C](http://www.yeastgenome.org/cgi-bin/locus.fpl?locus=ELP3), [NOG1/YPL093W](http://www.yeastgenome.org/cgi-bin/locus.fpl?locus=NOG1), [NAN1/YPL126W](http://www.yeastgenome.org/cgi-bin/locus.fpl?locus=NAN1), [NOP53/YPL146C](http://www.yeastgenome.org/cgi-bin/locus.fpl?locus=NOP53), [TYW1/YPL207W](http://www.yeastgenome.org/cgi-bin/locus.fpl?locus=TYW1), [NIP7/YPL211W](http://www.yeastgenome.org/cgi-bin/locus.fpl?locus=NIP7), [BMS1/YPL217C](http://www.yeastgenome.org/cgi-bin/locus.fpl?locus=BMS1) |
| [*nitrogen compound metabolic process*](http://www.yeastgenome.org/cgi-bin/GO/goTerm.pl?goid=6807) | 140 out of 292 genes, 47.9% | 1877 out of 7167 genes, 26.2% | 2.07e-13 | [GDH3/YAL062W](http://www.yeastgenome.org/cgi-bin/locus.fpl?locus=GDH3), [ADE1/YAR015W](http://www.yeastgenome.org/cgi-bin/locus.fpl?locus=ADE1), [UTP20/YBL004W](http://www.yeastgenome.org/cgi-bin/locus.fpl?locus=UTP20), [NCL1/YBL024W](http://www.yeastgenome.org/cgi-bin/locus.fpl?locus=NCL1), [URA7/YBL039C](http://www.yeastgenome.org/cgi-bin/locus.fpl?locus=URA7), [TOD6/YBL054W](http://www.yeastgenome.org/cgi-bin/locus.fpl?locus=TOD6), [HMT1/YBR034C](http://www.yeastgenome.org/cgi-bin/locus.fpl?locus=HMT1), [POL30/YBR088C](http://www.yeastgenome.org/cgi-bin/locus.fpl?locus=POL30), [PHO3/YBR092C](http://www.yeastgenome.org/cgi-bin/locus.fpl?locus=PHO3), [TKL2/YBR117C](http://www.yeastgenome.org/cgi-bin/locus.fpl?locus=TKL2), [MAK5/YBR142W](http://www.yeastgenome.org/cgi-bin/locus.fpl?locus=MAK5), [DUR1,2/YBR208C](http://www.yeastgenome.org/cgi-bin/locus.fpl?locus=DUR1), [ENP1/YBR247C](http://www.yeastgenome.org/cgi-bin/locus.fpl?locus=ENP1), [SHM1/YBR263W](http://www.yeastgenome.org/cgi-bin/locus.fpl?locus=SHM1), [SPB1/YCL054W](http://www.yeastgenome.org/cgi-bin/locus.fpl?locus=SPB1), [MATALPHA1/YCR040W](http://www.yeastgenome.org/cgi-bin/locus.fpl?locus=MATALPHA1), [BUD31/YCR063W](http://www.yeastgenome.org/cgi-bin/locus.fpl?locus=BUD31), [MCD1/YDL003W](http://www.yeastgenome.org/cgi-bin/locus.fpl?locus=MCD1), [TSR1/YDL060W](http://www.yeastgenome.org/cgi-bin/locus.fpl?locus=TSR1), [NOP14/YDL148C](http://www.yeastgenome.org/cgi-bin/locus.fpl?locus=NOP14), [SAS10/YDL153C](http://www.yeastgenome.org/cgi-bin/locus.fpl?locus=SAS10), [TRM8/YDL201W](http://www.yeastgenome.org/cgi-bin/locus.fpl?locus=TRM8), [GDH2/YDL215C](http://www.yeastgenome.org/cgi-bin/locus.fpl?locus=GDH2), [RRP1/YDR087C](http://www.yeastgenome.org/cgi-bin/locus.fpl?locus=RRP1), [SUP35/YDR172W](http://www.yeastgenome.org/cgi-bin/locus.fpl?locus=SUP35), [LYS4/YDR234W](http://www.yeastgenome.org/cgi-bin/locus.fpl?locus=LYS4), [ASP1/YDR321W](http://www.yeastgenome.org/cgi-bin/locus.fpl?locus=ASP1), [UTP4/YDR324C](http://www.yeastgenome.org/cgi-bin/locus.fpl?locus=UTP4), [NCB2/YDR397C](http://www.yeastgenome.org/cgi-bin/locus.fpl?locus=NCB2), [UTP5/YDR398W](http://www.yeastgenome.org/cgi-bin/locus.fpl?locus=UTP5), [HPT1/YDR399W](http://www.yeastgenome.org/cgi-bin/locus.fpl?locus=HPT1), [RRP17/YDR412W](http://www.yeastgenome.org/cgi-bin/locus.fpl?locus=RRP17), [DOT1/YDR440W](http://www.yeastgenome.org/cgi-bin/locus.fpl?locus=DOT1), [UTP6/YDR449C](http://www.yeastgenome.org/cgi-bin/locus.fpl?locus=UTP6), [SNU13/YEL026W](http://www.yeastgenome.org/cgi-bin/locus.fpl?locus=SNU13), [HPA3/YEL066W](http://www.yeastgenome.org/cgi-bin/locus.fpl?locus=HPA3), [PMI40/YER003C](http://www.yeastgenome.org/cgi-bin/locus.fpl?locus=PMI40), [NUG1/YER006W](http://www.yeastgenome.org/cgi-bin/locus.fpl?locus=NUG1), [SAH1/YER043C](http://www.yeastgenome.org/cgi-bin/locus.fpl?locus=SAH1), [YER064C](http://www.yeastgenome.org/cgi-bin/locus.fpl?locus=YER064C), [RNR1/YER070W](http://www.yeastgenome.org/cgi-bin/locus.fpl?locus=RNR1), [UTP7/YER082C](http://www.yeastgenome.org/cgi-bin/locus.fpl?locus=UTP7), [DBP3/YGL078C](http://www.yeastgenome.org/cgi-bin/locus.fpl?locus=DBP3), [PRP43/YGL120C](http://www.yeastgenome.org/cgi-bin/locus.fpl?locus=PRP43), [ADE5,7/YGL234W](http://www.yeastgenome.org/cgi-bin/locus.fpl?locus=ADE5), [HXK2/YGL253W](http://www.yeastgenome.org/cgi-bin/locus.fpl?locus=HXK2), [PDC6/YGR087C](http://www.yeastgenome.org/cgi-bin/locus.fpl?locus=PDC6), [NOP7/YGR103W](http://www.yeastgenome.org/cgi-bin/locus.fpl?locus=NOP7), [ENP2/YGR145W](http://www.yeastgenome.org/cgi-bin/locus.fpl?locus=ENP2), [CYS4/YGR155W](http://www.yeastgenome.org/cgi-bin/locus.fpl?locus=CYS4), [CHO2/YGR157W](http://www.yeastgenome.org/cgi-bin/locus.fpl?locus=CHO2), [NSR1/YGR159C](http://www.yeastgenome.org/cgi-bin/locus.fpl?locus=NSR1), [CRH1/YGR189C](http://www.yeastgenome.org/cgi-bin/locus.fpl?locus=CRH1), [GND2/YGR256W](http://www.yeastgenome.org/cgi-bin/locus.fpl?locus=GND2), [PXR1/YGR280C](http://www.yeastgenome.org/cgi-bin/locus.fpl?locus=PXR1), [DUR3/YHL016C](http://www.yeastgenome.org/cgi-bin/locus.fpl?locus=DUR3), [GAR1/YHR089C](http://www.yeastgenome.org/cgi-bin/locus.fpl?locus=GAR1), [GND1/YHR183W](http://www.yeastgenome.org/cgi-bin/locus.fpl?locus=GND1), [RIX1/YHR197W](http://www.yeastgenome.org/cgi-bin/locus.fpl?locus=RIX1), [IMD2/YHR216W](http://www.yeastgenome.org/cgi-bin/locus.fpl?locus=IMD2), [DAL2/YIR029W](http://www.yeastgenome.org/cgi-bin/locus.fpl?locus=DAL2), [RPC17/YJL011C](http://www.yeastgenome.org/cgi-bin/locus.fpl?locus=RPC17), [MTR4/YJL050W](http://www.yeastgenome.org/cgi-bin/locus.fpl?locus=MTR4), [UTP18/YJL069C](http://www.yeastgenome.org/cgi-bin/locus.fpl?locus=UTP18), [SCP160/YJL080C](http://www.yeastgenome.org/cgi-bin/locus.fpl?locus=SCP160), [URA2/YJL130C](http://www.yeastgenome.org/cgi-bin/locus.fpl?locus=URA2), [CPS1/YJL172W](http://www.yeastgenome.org/cgi-bin/locus.fpl?locus=CPS1), [MPP10/YJR002W](http://www.yeastgenome.org/cgi-bin/locus.fpl?locus=MPP10), [RPA12/YJR063W](http://www.yeastgenome.org/cgi-bin/locus.fpl?locus=RPA12), [URA8/YJR103W](http://www.yeastgenome.org/cgi-bin/locus.fpl?locus=URA8), [BAT2/YJR148W](http://www.yeastgenome.org/cgi-bin/locus.fpl?locus=BAT2), [MAK11/YKL021C](http://www.yeastgenome.org/cgi-bin/locus.fpl?locus=MAK11), [EBP2/YKL172W](http://www.yeastgenome.org/cgi-bin/locus.fpl?locus=EBP2), [GAP1/YKR039W](http://www.yeastgenome.org/cgi-bin/locus.fpl?locus=GAP1), [RPF2/YKR081C](http://www.yeastgenome.org/cgi-bin/locus.fpl?locus=RPF2), [SPT8/YLR055C](http://www.yeastgenome.org/cgi-bin/locus.fpl?locus=SPT8), [FRS1/YLR060W](http://www.yeastgenome.org/cgi-bin/locus.fpl?locus=FRS1), [MDN1/YLR106C](http://www.yeastgenome.org/cgi-bin/locus.fpl?locus=MDN1), [PDC5/YLR134W](http://www.yeastgenome.org/cgi-bin/locus.fpl?locus=PDC5), [PUT1/YLR142W](http://www.yeastgenome.org/cgi-bin/locus.fpl?locus=PUT1), [STM1/YLR150W](http://www.yeastgenome.org/cgi-bin/locus.fpl?locus=STM1), [CBF5/YLR175W](http://www.yeastgenome.org/cgi-bin/locus.fpl?locus=CBF5), [PWP1/YLR196W](http://www.yeastgenome.org/cgi-bin/locus.fpl?locus=PWP1), [NOP56/YLR197W](http://www.yeastgenome.org/cgi-bin/locus.fpl?locus=NOP56), [UTP13/YLR222C](http://www.yeastgenome.org/cgi-bin/locus.fpl?locus=UTP13), [DBP9/YLR276C](http://www.yeastgenome.org/cgi-bin/locus.fpl?locus=DBP9), [GSP1/YLR293C](http://www.yeastgenome.org/cgi-bin/locus.fpl?locus=GSP1), [ADE13/YLR359W](http://www.yeastgenome.org/cgi-bin/locus.fpl?locus=ADE13), [DUS3/YLR401C](http://www.yeastgenome.org/cgi-bin/locus.fpl?locus=DUS3), [IMD3/YLR432W](http://www.yeastgenome.org/cgi-bin/locus.fpl?locus=IMD3), [FPR4/YLR449W](http://www.yeastgenome.org/cgi-bin/locus.fpl?locus=FPR4), [LEU3/YLR451W](http://www.yeastgenome.org/cgi-bin/locus.fpl?locus=LEU3), [TSA1/YML028W](http://www.yeastgenome.org/cgi-bin/locus.fpl?locus=TSA1), [ERB1/YMR049C](http://www.yeastgenome.org/cgi-bin/locus.fpl?locus=ERB1), [PDS5/YMR076C](http://www.yeastgenome.org/cgi-bin/locus.fpl?locus=PDS5), [UTP15/YMR093W](http://www.yeastgenome.org/cgi-bin/locus.fpl?locus=UTP15), [ADE17/YMR120C](http://www.yeastgenome.org/cgi-bin/locus.fpl?locus=ADE17), [ECM16/YMR128W](http://www.yeastgenome.org/cgi-bin/locus.fpl?locus=ECM16), [NDE1/YMR145C](http://www.yeastgenome.org/cgi-bin/locus.fpl?locus=NDE1), [GUA1/YMR217W](http://www.yeastgenome.org/cgi-bin/locus.fpl?locus=GUA1), [GAS1/YMR307W](http://www.yeastgenome.org/cgi-bin/locus.fpl?locus=GAS1), [NOP2/YNL061W](http://www.yeastgenome.org/cgi-bin/locus.fpl?locus=NOP2), [POL1/YNL102W](http://www.yeastgenome.org/cgi-bin/locus.fpl?locus=POL1), [DBP2/YNL112W](http://www.yeastgenome.org/cgi-bin/locus.fpl?locus=DBP2), [RPC19/YNL113W](http://www.yeastgenome.org/cgi-bin/locus.fpl?locus=RPC19), [AAH1/YNL141W](http://www.yeastgenome.org/cgi-bin/locus.fpl?locus=AAH1), [IPI3/YNL182C](http://www.yeastgenome.org/cgi-bin/locus.fpl?locus=IPI3), [PUS4/YNL292W](http://www.yeastgenome.org/cgi-bin/locus.fpl?locus=PUS4), [KRI1/YNL308C](http://www.yeastgenome.org/cgi-bin/locus.fpl?locus=KRI1), [TRM112/YNR046W](http://www.yeastgenome.org/cgi-bin/locus.fpl?locus=TRM112), [LYS9/YNR050C](http://www.yeastgenome.org/cgi-bin/locus.fpl?locus=LYS9), [TRM11/YOL124C](http://www.yeastgenome.org/cgi-bin/locus.fpl?locus=TRM11), [CDC33/YOL139C](http://www.yeastgenome.org/cgi-bin/locus.fpl?locus=CDC33), [RRP6/YOR001W](http://www.yeastgenome.org/cgi-bin/locus.fpl?locus=RRP6), [CDC21/YOR074C](http://www.yeastgenome.org/cgi-bin/locus.fpl?locus=CDC21), [RAS1/YOR101W](http://www.yeastgenome.org/cgi-bin/locus.fpl?locus=RAS1), [RPO31/YOR116C](http://www.yeastgenome.org/cgi-bin/locus.fpl?locus=RPO31), [ARP8/YOR141C](http://www.yeastgenome.org/cgi-bin/locus.fpl?locus=ARP8), [PNO1/YOR145C](http://www.yeastgenome.org/cgi-bin/locus.fpl?locus=PNO1), [GLN4/YOR168W](http://www.yeastgenome.org/cgi-bin/locus.fpl?locus=GLN4), [RRS1/YOR294W](http://www.yeastgenome.org/cgi-bin/locus.fpl?locus=RRS1), [NOP58/YOR310C](http://www.yeastgenome.org/cgi-bin/locus.fpl?locus=NOP58), [RPA43/YOR340C](http://www.yeastgenome.org/cgi-bin/locus.fpl?locus=RPA43), [PUT4/YOR348C](http://www.yeastgenome.org/cgi-bin/locus.fpl?locus=PUT4), [GDH1/YOR375C](http://www.yeastgenome.org/cgi-bin/locus.fpl?locus=GDH1), [RRP12/YPL012W](http://www.yeastgenome.org/cgi-bin/locus.fpl?locus=RRP12), [NOP4/YPL043W](http://www.yeastgenome.org/cgi-bin/locus.fpl?locus=NOP4), [ELP3/YPL086C](http://www.yeastgenome.org/cgi-bin/locus.fpl?locus=ELP3), [NOG1/YPL093W](http://www.yeastgenome.org/cgi-bin/locus.fpl?locus=NOG1), [CAR1/YPL111W](http://www.yeastgenome.org/cgi-bin/locus.fpl?locus=CAR1), [NAN1/YPL126W](http://www.yeastgenome.org/cgi-bin/locus.fpl?locus=NAN1), [NOP53/YPL146C](http://www.yeastgenome.org/cgi-bin/locus.fpl?locus=NOP53), [TYW1/YPL207W](http://www.yeastgenome.org/cgi-bin/locus.fpl?locus=TYW1), [NIP7/YPL211W](http://www.yeastgenome.org/cgi-bin/locus.fpl?locus=NIP7), [BMS1/YPL217C](http://www.yeastgenome.org/cgi-bin/locus.fpl?locus=BMS1), [RPA135/YPR010C](http://www.yeastgenome.org/cgi-bin/locus.fpl?locus=RPA135), [CSR2/YPR030W](http://www.yeastgenome.org/cgi-bin/locus.fpl?locus=CSR2), [GLN1/YPR035W](http://www.yeastgenome.org/cgi-bin/locus.fpl?locus=GLN1), [TKL1/YPR074C](http://www.yeastgenome.org/cgi-bin/locus.fpl?locus=TKL1), [RPC82/YPR190C](http://www.yeastgenome.org/cgi-bin/locus.fpl?locus=RPC82) |
| [*cellular component biogenesis*](http://www.yeastgenome.org/cgi-bin/GO/goTerm.pl?goid=44085) | 87 out of 292 genes, 29.8% | 898 out of 7167 genes, 12.5% | 6.43e-13 | [UTP20/YBL004W](http://www.yeastgenome.org/cgi-bin/locus.fpl?locus=UTP20), [MAK5/YBR142W](http://www.yeastgenome.org/cgi-bin/locus.fpl?locus=MAK5), [ENP1/YBR247C](http://www.yeastgenome.org/cgi-bin/locus.fpl?locus=ENP1), [KCC4/YCL024W](http://www.yeastgenome.org/cgi-bin/locus.fpl?locus=KCC4), [SPB1/YCL054W](http://www.yeastgenome.org/cgi-bin/locus.fpl?locus=SPB1), [TSR1/YDL060W](http://www.yeastgenome.org/cgi-bin/locus.fpl?locus=TSR1), [YDL063C](http://www.yeastgenome.org/cgi-bin/locus.fpl?locus=YDL063C), [NOP14/YDL148C](http://www.yeastgenome.org/cgi-bin/locus.fpl?locus=NOP14), [SAS10/YDL153C](http://www.yeastgenome.org/cgi-bin/locus.fpl?locus=SAS10), [NOP6/YDL213C](http://www.yeastgenome.org/cgi-bin/locus.fpl?locus=NOP6), [MAK21/YDR060W](http://www.yeastgenome.org/cgi-bin/locus.fpl?locus=MAK21), [RRP1/YDR087C](http://www.yeastgenome.org/cgi-bin/locus.fpl?locus=RRP1), [ARX1/YDR101C](http://www.yeastgenome.org/cgi-bin/locus.fpl?locus=ARX1), [SSF2/YDR312W](http://www.yeastgenome.org/cgi-bin/locus.fpl?locus=SSF2), [UTP4/YDR324C](http://www.yeastgenome.org/cgi-bin/locus.fpl?locus=UTP4), [UTP5/YDR398W](http://www.yeastgenome.org/cgi-bin/locus.fpl?locus=UTP5), [RRP17/YDR412W](http://www.yeastgenome.org/cgi-bin/locus.fpl?locus=RRP17), [UTP6/YDR449C](http://www.yeastgenome.org/cgi-bin/locus.fpl?locus=UTP6), [PUF6/YDR496C](http://www.yeastgenome.org/cgi-bin/locus.fpl?locus=PUF6), [SNU13/YEL026W](http://www.yeastgenome.org/cgi-bin/locus.fpl?locus=SNU13), [PMI40/YER003C](http://www.yeastgenome.org/cgi-bin/locus.fpl?locus=PMI40), [NUG1/YER006W](http://www.yeastgenome.org/cgi-bin/locus.fpl?locus=NUG1), [ARB1/YER036C](http://www.yeastgenome.org/cgi-bin/locus.fpl?locus=ARB1), [UTP7/YER082C](http://www.yeastgenome.org/cgi-bin/locus.fpl?locus=UTP7), [BUD27/YFL023W](http://www.yeastgenome.org/cgi-bin/locus.fpl?locus=BUD27), [DBP3/YGL078C](http://www.yeastgenome.org/cgi-bin/locus.fpl?locus=DBP3), [LSG1/YGL099W](http://www.yeastgenome.org/cgi-bin/locus.fpl?locus=LSG1), [PRP43/YGL120C](http://www.yeastgenome.org/cgi-bin/locus.fpl?locus=PRP43), [NOP7/YGR103W](http://www.yeastgenome.org/cgi-bin/locus.fpl?locus=NOP7), [ENP2/YGR145W](http://www.yeastgenome.org/cgi-bin/locus.fpl?locus=ENP2), [NSR1/YGR159C](http://www.yeastgenome.org/cgi-bin/locus.fpl?locus=NSR1), [TIF4631/YGR162W](http://www.yeastgenome.org/cgi-bin/locus.fpl?locus=TIF4631), [SDA1/YGR245C](http://www.yeastgenome.org/cgi-bin/locus.fpl?locus=SDA1), [CIC1/YHR052W](http://www.yeastgenome.org/cgi-bin/locus.fpl?locus=CIC1), [GAR1/YHR089C](http://www.yeastgenome.org/cgi-bin/locus.fpl?locus=GAR1), [NMD3/YHR170W](http://www.yeastgenome.org/cgi-bin/locus.fpl?locus=NMD3), [RIX1/YHR197W](http://www.yeastgenome.org/cgi-bin/locus.fpl?locus=RIX1), [SEC24/YIL109C](http://www.yeastgenome.org/cgi-bin/locus.fpl?locus=SEC24), [SQT1/YIR012W](http://www.yeastgenome.org/cgi-bin/locus.fpl?locus=SQT1), [RPC17/YJL011C](http://www.yeastgenome.org/cgi-bin/locus.fpl?locus=RPC17), [MTR4/YJL050W](http://www.yeastgenome.org/cgi-bin/locus.fpl?locus=MTR4), [UTP18/YJL069C](http://www.yeastgenome.org/cgi-bin/locus.fpl?locus=UTP18), [ALB1/YJL122W](http://www.yeastgenome.org/cgi-bin/locus.fpl?locus=ALB1), [MPP10/YJR002W](http://www.yeastgenome.org/cgi-bin/locus.fpl?locus=MPP10), [MAK11/YKL021C](http://www.yeastgenome.org/cgi-bin/locus.fpl?locus=MAK11), [LTV1/YKL143W](http://www.yeastgenome.org/cgi-bin/locus.fpl?locus=LTV1), [EBP2/YKL172W](http://www.yeastgenome.org/cgi-bin/locus.fpl?locus=EBP2), [RPF2/YKR081C](http://www.yeastgenome.org/cgi-bin/locus.fpl?locus=RPF2), [RIX7/YLL034C](http://www.yeastgenome.org/cgi-bin/locus.fpl?locus=RIX7), [RLP24/YLR009W](http://www.yeastgenome.org/cgi-bin/locus.fpl?locus=RLP24), [MDN1/YLR106C](http://www.yeastgenome.org/cgi-bin/locus.fpl?locus=MDN1), [CBF5/YLR175W](http://www.yeastgenome.org/cgi-bin/locus.fpl?locus=CBF5), [PWP1/YLR196W](http://www.yeastgenome.org/cgi-bin/locus.fpl?locus=PWP1), [NOP56/YLR197W](http://www.yeastgenome.org/cgi-bin/locus.fpl?locus=NOP56), [UTP13/YLR222C](http://www.yeastgenome.org/cgi-bin/locus.fpl?locus=UTP13), [DBP9/YLR276C](http://www.yeastgenome.org/cgi-bin/locus.fpl?locus=DBP9), [GSP1/YLR293C](http://www.yeastgenome.org/cgi-bin/locus.fpl?locus=GSP1), [SGD1/YLR336C](http://www.yeastgenome.org/cgi-bin/locus.fpl?locus=SGD1), [FPR4/YLR449W](http://www.yeastgenome.org/cgi-bin/locus.fpl?locus=FPR4), [ERB1/YMR049C](http://www.yeastgenome.org/cgi-bin/locus.fpl?locus=ERB1), [UTP15/YMR093W](http://www.yeastgenome.org/cgi-bin/locus.fpl?locus=UTP15), [ECM16/YMR128W](http://www.yeastgenome.org/cgi-bin/locus.fpl?locus=ECM16), [RRB1/YMR131C](http://www.yeastgenome.org/cgi-bin/locus.fpl?locus=RRB1), [PEP5/YMR231W](http://www.yeastgenome.org/cgi-bin/locus.fpl?locus=PEP5), [DFG5/YMR238W](http://www.yeastgenome.org/cgi-bin/locus.fpl?locus=DFG5), [NOP2/YNL061W](http://www.yeastgenome.org/cgi-bin/locus.fpl?locus=NOP2), [NOP15/YNL110C](http://www.yeastgenome.org/cgi-bin/locus.fpl?locus=NOP15), [DBP2/YNL112W](http://www.yeastgenome.org/cgi-bin/locus.fpl?locus=DBP2), [KRE33/YNL132W](http://www.yeastgenome.org/cgi-bin/locus.fpl?locus=KRE33), [IPI3/YNL182C](http://www.yeastgenome.org/cgi-bin/locus.fpl?locus=IPI3), [KRI1/YNL308C](http://www.yeastgenome.org/cgi-bin/locus.fpl?locus=KRI1), [NOG2/YNR053C](http://www.yeastgenome.org/cgi-bin/locus.fpl?locus=NOG2), [BRX1/YOL077C](http://www.yeastgenome.org/cgi-bin/locus.fpl?locus=BRX1), [RRP6/YOR001W](http://www.yeastgenome.org/cgi-bin/locus.fpl?locus=RRP6), [PNO1/YOR145C](http://www.yeastgenome.org/cgi-bin/locus.fpl?locus=PNO1), [NOC2/YOR206W](http://www.yeastgenome.org/cgi-bin/locus.fpl?locus=NOC2), [YTM1/YOR272W](http://www.yeastgenome.org/cgi-bin/locus.fpl?locus=YTM1), [RRS1/YOR294W](http://www.yeastgenome.org/cgi-bin/locus.fpl?locus=RRS1), [NOP58/YOR310C](http://www.yeastgenome.org/cgi-bin/locus.fpl?locus=NOP58), [RRP12/YPL012W](http://www.yeastgenome.org/cgi-bin/locus.fpl?locus=RRP12), [NOP4/YPL043W](http://www.yeastgenome.org/cgi-bin/locus.fpl?locus=NOP4), [NOG1/YPL093W](http://www.yeastgenome.org/cgi-bin/locus.fpl?locus=NOG1), [NAN1/YPL126W](http://www.yeastgenome.org/cgi-bin/locus.fpl?locus=NAN1), [NOP53/YPL146C](http://www.yeastgenome.org/cgi-bin/locus.fpl?locus=NOP53), [NIP7/YPL211W](http://www.yeastgenome.org/cgi-bin/locus.fpl?locus=NIP7), [BMS1/YPL217C](http://www.yeastgenome.org/cgi-bin/locus.fpl?locus=BMS1), [NEW1/YPL226W](http://www.yeastgenome.org/cgi-bin/locus.fpl?locus=NEW1) |
| [*cellular nitrogen compound metabolic process*](http://www.yeastgenome.org/cgi-bin/GO/goTerm.pl?goid=34641) | 136 out of 292 genes, 46.6% | 1850 out of 7167 genes, 25.8% | 3.01e-12 | [GDH3/YAL062W](http://www.yeastgenome.org/cgi-bin/locus.fpl?locus=GDH3), [ADE1/YAR015W](http://www.yeastgenome.org/cgi-bin/locus.fpl?locus=ADE1), [UTP20/YBL004W](http://www.yeastgenome.org/cgi-bin/locus.fpl?locus=UTP20), [NCL1/YBL024W](http://www.yeastgenome.org/cgi-bin/locus.fpl?locus=NCL1), [URA7/YBL039C](http://www.yeastgenome.org/cgi-bin/locus.fpl?locus=URA7), [TOD6/YBL054W](http://www.yeastgenome.org/cgi-bin/locus.fpl?locus=TOD6), [HMT1/YBR034C](http://www.yeastgenome.org/cgi-bin/locus.fpl?locus=HMT1), [POL30/YBR088C](http://www.yeastgenome.org/cgi-bin/locus.fpl?locus=POL30), [TKL2/YBR117C](http://www.yeastgenome.org/cgi-bin/locus.fpl?locus=TKL2), [MAK5/YBR142W](http://www.yeastgenome.org/cgi-bin/locus.fpl?locus=MAK5), [DUR1,2/YBR208C](http://www.yeastgenome.org/cgi-bin/locus.fpl?locus=DUR1), [ENP1/YBR247C](http://www.yeastgenome.org/cgi-bin/locus.fpl?locus=ENP1), [SHM1/YBR263W](http://www.yeastgenome.org/cgi-bin/locus.fpl?locus=SHM1), [SPB1/YCL054W](http://www.yeastgenome.org/cgi-bin/locus.fpl?locus=SPB1), [MATALPHA1/YCR040W](http://www.yeastgenome.org/cgi-bin/locus.fpl?locus=MATALPHA1), [BUD31/YCR063W](http://www.yeastgenome.org/cgi-bin/locus.fpl?locus=BUD31), [MCD1/YDL003W](http://www.yeastgenome.org/cgi-bin/locus.fpl?locus=MCD1), [TSR1/YDL060W](http://www.yeastgenome.org/cgi-bin/locus.fpl?locus=TSR1), [NOP14/YDL148C](http://www.yeastgenome.org/cgi-bin/locus.fpl?locus=NOP14), [SAS10/YDL153C](http://www.yeastgenome.org/cgi-bin/locus.fpl?locus=SAS10), [TRM8/YDL201W](http://www.yeastgenome.org/cgi-bin/locus.fpl?locus=TRM8), [RRP1/YDR087C](http://www.yeastgenome.org/cgi-bin/locus.fpl?locus=RRP1), [SUP35/YDR172W](http://www.yeastgenome.org/cgi-bin/locus.fpl?locus=SUP35), [LYS4/YDR234W](http://www.yeastgenome.org/cgi-bin/locus.fpl?locus=LYS4), [ASP1/YDR321W](http://www.yeastgenome.org/cgi-bin/locus.fpl?locus=ASP1), [UTP4/YDR324C](http://www.yeastgenome.org/cgi-bin/locus.fpl?locus=UTP4), [NCB2/YDR397C](http://www.yeastgenome.org/cgi-bin/locus.fpl?locus=NCB2), [UTP5/YDR398W](http://www.yeastgenome.org/cgi-bin/locus.fpl?locus=UTP5), [HPT1/YDR399W](http://www.yeastgenome.org/cgi-bin/locus.fpl?locus=HPT1), [RRP17/YDR412W](http://www.yeastgenome.org/cgi-bin/locus.fpl?locus=RRP17), [DOT1/YDR440W](http://www.yeastgenome.org/cgi-bin/locus.fpl?locus=DOT1), [UTP6/YDR449C](http://www.yeastgenome.org/cgi-bin/locus.fpl?locus=UTP6), [SNU13/YEL026W](http://www.yeastgenome.org/cgi-bin/locus.fpl?locus=SNU13), [HPA3/YEL066W](http://www.yeastgenome.org/cgi-bin/locus.fpl?locus=HPA3), [PMI40/YER003C](http://www.yeastgenome.org/cgi-bin/locus.fpl?locus=PMI40), [NUG1/YER006W](http://www.yeastgenome.org/cgi-bin/locus.fpl?locus=NUG1), [SAH1/YER043C](http://www.yeastgenome.org/cgi-bin/locus.fpl?locus=SAH1), [YER064C](http://www.yeastgenome.org/cgi-bin/locus.fpl?locus=YER064C), [RNR1/YER070W](http://www.yeastgenome.org/cgi-bin/locus.fpl?locus=RNR1), [UTP7/YER082C](http://www.yeastgenome.org/cgi-bin/locus.fpl?locus=UTP7), [DBP3/YGL078C](http://www.yeastgenome.org/cgi-bin/locus.fpl?locus=DBP3), [PRP43/YGL120C](http://www.yeastgenome.org/cgi-bin/locus.fpl?locus=PRP43), [ADE5,7/YGL234W](http://www.yeastgenome.org/cgi-bin/locus.fpl?locus=ADE5), [HXK2/YGL253W](http://www.yeastgenome.org/cgi-bin/locus.fpl?locus=HXK2), [PDC6/YGR087C](http://www.yeastgenome.org/cgi-bin/locus.fpl?locus=PDC6), [NOP7/YGR103W](http://www.yeastgenome.org/cgi-bin/locus.fpl?locus=NOP7), [ENP2/YGR145W](http://www.yeastgenome.org/cgi-bin/locus.fpl?locus=ENP2), [CYS4/YGR155W](http://www.yeastgenome.org/cgi-bin/locus.fpl?locus=CYS4), [CHO2/YGR157W](http://www.yeastgenome.org/cgi-bin/locus.fpl?locus=CHO2), [NSR1/YGR159C](http://www.yeastgenome.org/cgi-bin/locus.fpl?locus=NSR1), [GND2/YGR256W](http://www.yeastgenome.org/cgi-bin/locus.fpl?locus=GND2), [PXR1/YGR280C](http://www.yeastgenome.org/cgi-bin/locus.fpl?locus=PXR1), [DUR3/YHL016C](http://www.yeastgenome.org/cgi-bin/locus.fpl?locus=DUR3), [GAR1/YHR089C](http://www.yeastgenome.org/cgi-bin/locus.fpl?locus=GAR1), [GND1/YHR183W](http://www.yeastgenome.org/cgi-bin/locus.fpl?locus=GND1), [RIX1/YHR197W](http://www.yeastgenome.org/cgi-bin/locus.fpl?locus=RIX1), [IMD2/YHR216W](http://www.yeastgenome.org/cgi-bin/locus.fpl?locus=IMD2), [DAL2/YIR029W](http://www.yeastgenome.org/cgi-bin/locus.fpl?locus=DAL2), [RPC17/YJL011C](http://www.yeastgenome.org/cgi-bin/locus.fpl?locus=RPC17), [MTR4/YJL050W](http://www.yeastgenome.org/cgi-bin/locus.fpl?locus=MTR4), [UTP18/YJL069C](http://www.yeastgenome.org/cgi-bin/locus.fpl?locus=UTP18), [SCP160/YJL080C](http://www.yeastgenome.org/cgi-bin/locus.fpl?locus=SCP160), [URA2/YJL130C](http://www.yeastgenome.org/cgi-bin/locus.fpl?locus=URA2), [MPP10/YJR002W](http://www.yeastgenome.org/cgi-bin/locus.fpl?locus=MPP10), [RPA12/YJR063W](http://www.yeastgenome.org/cgi-bin/locus.fpl?locus=RPA12), [URA8/YJR103W](http://www.yeastgenome.org/cgi-bin/locus.fpl?locus=URA8), [BAT2/YJR148W](http://www.yeastgenome.org/cgi-bin/locus.fpl?locus=BAT2), [MAK11/YKL021C](http://www.yeastgenome.org/cgi-bin/locus.fpl?locus=MAK11), [EBP2/YKL172W](http://www.yeastgenome.org/cgi-bin/locus.fpl?locus=EBP2), [GAP1/YKR039W](http://www.yeastgenome.org/cgi-bin/locus.fpl?locus=GAP1), [RPF2/YKR081C](http://www.yeastgenome.org/cgi-bin/locus.fpl?locus=RPF2), [SPT8/YLR055C](http://www.yeastgenome.org/cgi-bin/locus.fpl?locus=SPT8), [FRS1/YLR060W](http://www.yeastgenome.org/cgi-bin/locus.fpl?locus=FRS1), [MDN1/YLR106C](http://www.yeastgenome.org/cgi-bin/locus.fpl?locus=MDN1), [PDC5/YLR134W](http://www.yeastgenome.org/cgi-bin/locus.fpl?locus=PDC5), [PUT1/YLR142W](http://www.yeastgenome.org/cgi-bin/locus.fpl?locus=PUT1), [STM1/YLR150W](http://www.yeastgenome.org/cgi-bin/locus.fpl?locus=STM1), [CBF5/YLR175W](http://www.yeastgenome.org/cgi-bin/locus.fpl?locus=CBF5), [PWP1/YLR196W](http://www.yeastgenome.org/cgi-bin/locus.fpl?locus=PWP1), [NOP56/YLR197W](http://www.yeastgenome.org/cgi-bin/locus.fpl?locus=NOP56), [UTP13/YLR222C](http://www.yeastgenome.org/cgi-bin/locus.fpl?locus=UTP13), [DBP9/YLR276C](http://www.yeastgenome.org/cgi-bin/locus.fpl?locus=DBP9), [GSP1/YLR293C](http://www.yeastgenome.org/cgi-bin/locus.fpl?locus=GSP1), [ADE13/YLR359W](http://www.yeastgenome.org/cgi-bin/locus.fpl?locus=ADE13), [DUS3/YLR401C](http://www.yeastgenome.org/cgi-bin/locus.fpl?locus=DUS3), [IMD3/YLR432W](http://www.yeastgenome.org/cgi-bin/locus.fpl?locus=IMD3), [FPR4/YLR449W](http://www.yeastgenome.org/cgi-bin/locus.fpl?locus=FPR4), [LEU3/YLR451W](http://www.yeastgenome.org/cgi-bin/locus.fpl?locus=LEU3), [TSA1/YML028W](http://www.yeastgenome.org/cgi-bin/locus.fpl?locus=TSA1), [ERB1/YMR049C](http://www.yeastgenome.org/cgi-bin/locus.fpl?locus=ERB1), [PDS5/YMR076C](http://www.yeastgenome.org/cgi-bin/locus.fpl?locus=PDS5), [UTP15/YMR093W](http://www.yeastgenome.org/cgi-bin/locus.fpl?locus=UTP15), [ADE17/YMR120C](http://www.yeastgenome.org/cgi-bin/locus.fpl?locus=ADE17), [ECM16/YMR128W](http://www.yeastgenome.org/cgi-bin/locus.fpl?locus=ECM16), [NDE1/YMR145C](http://www.yeastgenome.org/cgi-bin/locus.fpl?locus=NDE1), [GUA1/YMR217W](http://www.yeastgenome.org/cgi-bin/locus.fpl?locus=GUA1), [GAS1/YMR307W](http://www.yeastgenome.org/cgi-bin/locus.fpl?locus=GAS1), [NOP2/YNL061W](http://www.yeastgenome.org/cgi-bin/locus.fpl?locus=NOP2), [POL1/YNL102W](http://www.yeastgenome.org/cgi-bin/locus.fpl?locus=POL1), [DBP2/YNL112W](http://www.yeastgenome.org/cgi-bin/locus.fpl?locus=DBP2), [RPC19/YNL113W](http://www.yeastgenome.org/cgi-bin/locus.fpl?locus=RPC19), [AAH1/YNL141W](http://www.yeastgenome.org/cgi-bin/locus.fpl?locus=AAH1), [IPI3/YNL182C](http://www.yeastgenome.org/cgi-bin/locus.fpl?locus=IPI3), [PUS4/YNL292W](http://www.yeastgenome.org/cgi-bin/locus.fpl?locus=PUS4), [KRI1/YNL308C](http://www.yeastgenome.org/cgi-bin/locus.fpl?locus=KRI1), [TRM112/YNR046W](http://www.yeastgenome.org/cgi-bin/locus.fpl?locus=TRM112), [LYS9/YNR050C](http://www.yeastgenome.org/cgi-bin/locus.fpl?locus=LYS9), [TRM11/YOL124C](http://www.yeastgenome.org/cgi-bin/locus.fpl?locus=TRM11), [CDC33/YOL139C](http://www.yeastgenome.org/cgi-bin/locus.fpl?locus=CDC33), [RRP6/YOR001W](http://www.yeastgenome.org/cgi-bin/locus.fpl?locus=RRP6), [CDC21/YOR074C](http://www.yeastgenome.org/cgi-bin/locus.fpl?locus=CDC21), [RAS1/YOR101W](http://www.yeastgenome.org/cgi-bin/locus.fpl?locus=RAS1), [RPO31/YOR116C](http://www.yeastgenome.org/cgi-bin/locus.fpl?locus=RPO31), [ARP8/YOR141C](http://www.yeastgenome.org/cgi-bin/locus.fpl?locus=ARP8), [PNO1/YOR145C](http://www.yeastgenome.org/cgi-bin/locus.fpl?locus=PNO1), [GLN4/YOR168W](http://www.yeastgenome.org/cgi-bin/locus.fpl?locus=GLN4), [RRS1/YOR294W](http://www.yeastgenome.org/cgi-bin/locus.fpl?locus=RRS1), [NOP58/YOR310C](http://www.yeastgenome.org/cgi-bin/locus.fpl?locus=NOP58), [RPA43/YOR340C](http://www.yeastgenome.org/cgi-bin/locus.fpl?locus=RPA43), [PUT4/YOR348C](http://www.yeastgenome.org/cgi-bin/locus.fpl?locus=PUT4), [GDH1/YOR375C](http://www.yeastgenome.org/cgi-bin/locus.fpl?locus=GDH1), [RRP12/YPL012W](http://www.yeastgenome.org/cgi-bin/locus.fpl?locus=RRP12), [NOP4/YPL043W](http://www.yeastgenome.org/cgi-bin/locus.fpl?locus=NOP4), [ELP3/YPL086C](http://www.yeastgenome.org/cgi-bin/locus.fpl?locus=ELP3), [NOG1/YPL093W](http://www.yeastgenome.org/cgi-bin/locus.fpl?locus=NOG1), [CAR1/YPL111W](http://www.yeastgenome.org/cgi-bin/locus.fpl?locus=CAR1), [NAN1/YPL126W](http://www.yeastgenome.org/cgi-bin/locus.fpl?locus=NAN1), [NOP53/YPL146C](http://www.yeastgenome.org/cgi-bin/locus.fpl?locus=NOP53), [TYW1/YPL207W](http://www.yeastgenome.org/cgi-bin/locus.fpl?locus=TYW1), [NIP7/YPL211W](http://www.yeastgenome.org/cgi-bin/locus.fpl?locus=NIP7), [BMS1/YPL217C](http://www.yeastgenome.org/cgi-bin/locus.fpl?locus=BMS1), [RPA135/YPR010C](http://www.yeastgenome.org/cgi-bin/locus.fpl?locus=RPA135), [CSR2/YPR030W](http://www.yeastgenome.org/cgi-bin/locus.fpl?locus=CSR2), [GLN1/YPR035W](http://www.yeastgenome.org/cgi-bin/locus.fpl?locus=GLN1), [TKL1/YPR074C](http://www.yeastgenome.org/cgi-bin/locus.fpl?locus=TKL1), [RPC82/YPR190C](http://www.yeastgenome.org/cgi-bin/locus.fpl?locus=RPC82) |
| [*RNA processing*](http://www.yeastgenome.org/cgi-bin/GO/goTerm.pl?goid=6396) | 63 out of 292 genes, 21.6% | 553 out of 7167 genes, 7.7% | 1.10e-11 | [UTP20/YBL004W](http://www.yeastgenome.org/cgi-bin/locus.fpl?locus=UTP20), [NCL1/YBL024W](http://www.yeastgenome.org/cgi-bin/locus.fpl?locus=NCL1), [MAK5/YBR142W](http://www.yeastgenome.org/cgi-bin/locus.fpl?locus=MAK5), [ENP1/YBR247C](http://www.yeastgenome.org/cgi-bin/locus.fpl?locus=ENP1), [SPB1/YCL054W](http://www.yeastgenome.org/cgi-bin/locus.fpl?locus=SPB1), [BUD31/YCR063W](http://www.yeastgenome.org/cgi-bin/locus.fpl?locus=BUD31), [TSR1/YDL060W](http://www.yeastgenome.org/cgi-bin/locus.fpl?locus=TSR1), [NOP14/YDL148C](http://www.yeastgenome.org/cgi-bin/locus.fpl?locus=NOP14), [SAS10/YDL153C](http://www.yeastgenome.org/cgi-bin/locus.fpl?locus=SAS10), [TRM8/YDL201W](http://www.yeastgenome.org/cgi-bin/locus.fpl?locus=TRM8), [RRP1/YDR087C](http://www.yeastgenome.org/cgi-bin/locus.fpl?locus=RRP1), [UTP4/YDR324C](http://www.yeastgenome.org/cgi-bin/locus.fpl?locus=UTP4), [UTP5/YDR398W](http://www.yeastgenome.org/cgi-bin/locus.fpl?locus=UTP5), [RRP17/YDR412W](http://www.yeastgenome.org/cgi-bin/locus.fpl?locus=RRP17), [UTP6/YDR449C](http://www.yeastgenome.org/cgi-bin/locus.fpl?locus=UTP6), [SNU13/YEL026W](http://www.yeastgenome.org/cgi-bin/locus.fpl?locus=SNU13), [NUG1/YER006W](http://www.yeastgenome.org/cgi-bin/locus.fpl?locus=NUG1), [UTP7/YER082C](http://www.yeastgenome.org/cgi-bin/locus.fpl?locus=UTP7), [DBP3/YGL078C](http://www.yeastgenome.org/cgi-bin/locus.fpl?locus=DBP3), [PRP43/YGL120C](http://www.yeastgenome.org/cgi-bin/locus.fpl?locus=PRP43), [NOP7/YGR103W](http://www.yeastgenome.org/cgi-bin/locus.fpl?locus=NOP7), [ENP2/YGR145W](http://www.yeastgenome.org/cgi-bin/locus.fpl?locus=ENP2), [NSR1/YGR159C](http://www.yeastgenome.org/cgi-bin/locus.fpl?locus=NSR1), [PXR1/YGR280C](http://www.yeastgenome.org/cgi-bin/locus.fpl?locus=PXR1), [GAR1/YHR089C](http://www.yeastgenome.org/cgi-bin/locus.fpl?locus=GAR1), [RIX1/YHR197W](http://www.yeastgenome.org/cgi-bin/locus.fpl?locus=RIX1), [MTR4/YJL050W](http://www.yeastgenome.org/cgi-bin/locus.fpl?locus=MTR4), [UTP18/YJL069C](http://www.yeastgenome.org/cgi-bin/locus.fpl?locus=UTP18), [MPP10/YJR002W](http://www.yeastgenome.org/cgi-bin/locus.fpl?locus=MPP10), [MAK11/YKL021C](http://www.yeastgenome.org/cgi-bin/locus.fpl?locus=MAK11), [EBP2/YKL172W](http://www.yeastgenome.org/cgi-bin/locus.fpl?locus=EBP2), [RPF2/YKR081C](http://www.yeastgenome.org/cgi-bin/locus.fpl?locus=RPF2), [MDN1/YLR106C](http://www.yeastgenome.org/cgi-bin/locus.fpl?locus=MDN1), [CBF5/YLR175W](http://www.yeastgenome.org/cgi-bin/locus.fpl?locus=CBF5), [PWP1/YLR196W](http://www.yeastgenome.org/cgi-bin/locus.fpl?locus=PWP1), [NOP56/YLR197W](http://www.yeastgenome.org/cgi-bin/locus.fpl?locus=NOP56), [UTP13/YLR222C](http://www.yeastgenome.org/cgi-bin/locus.fpl?locus=UTP13), [DBP9/YLR276C](http://www.yeastgenome.org/cgi-bin/locus.fpl?locus=DBP9), [GSP1/YLR293C](http://www.yeastgenome.org/cgi-bin/locus.fpl?locus=GSP1), [DUS3/YLR401C](http://www.yeastgenome.org/cgi-bin/locus.fpl?locus=DUS3), [ERB1/YMR049C](http://www.yeastgenome.org/cgi-bin/locus.fpl?locus=ERB1), [UTP15/YMR093W](http://www.yeastgenome.org/cgi-bin/locus.fpl?locus=UTP15), [ECM16/YMR128W](http://www.yeastgenome.org/cgi-bin/locus.fpl?locus=ECM16), [NOP2/YNL061W](http://www.yeastgenome.org/cgi-bin/locus.fpl?locus=NOP2), [DBP2/YNL112W](http://www.yeastgenome.org/cgi-bin/locus.fpl?locus=DBP2), [IPI3/YNL182C](http://www.yeastgenome.org/cgi-bin/locus.fpl?locus=IPI3), [PUS4/YNL292W](http://www.yeastgenome.org/cgi-bin/locus.fpl?locus=PUS4), [KRI1/YNL308C](http://www.yeastgenome.org/cgi-bin/locus.fpl?locus=KRI1), [TRM112/YNR046W](http://www.yeastgenome.org/cgi-bin/locus.fpl?locus=TRM112), [TRM11/YOL124C](http://www.yeastgenome.org/cgi-bin/locus.fpl?locus=TRM11), [RRP6/YOR001W](http://www.yeastgenome.org/cgi-bin/locus.fpl?locus=RRP6), [PNO1/YOR145C](http://www.yeastgenome.org/cgi-bin/locus.fpl?locus=PNO1), [RRS1/YOR294W](http://www.yeastgenome.org/cgi-bin/locus.fpl?locus=RRS1), [NOP58/YOR310C](http://www.yeastgenome.org/cgi-bin/locus.fpl?locus=NOP58), [RRP12/YPL012W](http://www.yeastgenome.org/cgi-bin/locus.fpl?locus=RRP12), [NOP4/YPL043W](http://www.yeastgenome.org/cgi-bin/locus.fpl?locus=NOP4), [ELP3/YPL086C](http://www.yeastgenome.org/cgi-bin/locus.fpl?locus=ELP3), [NOG1/YPL093W](http://www.yeastgenome.org/cgi-bin/locus.fpl?locus=NOG1), [NAN1/YPL126W](http://www.yeastgenome.org/cgi-bin/locus.fpl?locus=NAN1), [NOP53/YPL146C](http://www.yeastgenome.org/cgi-bin/locus.fpl?locus=NOP53), [TYW1/YPL207W](http://www.yeastgenome.org/cgi-bin/locus.fpl?locus=TYW1), [NIP7/YPL211W](http://www.yeastgenome.org/cgi-bin/locus.fpl?locus=NIP7), [BMS1/YPL217C](http://www.yeastgenome.org/cgi-bin/locus.fpl?locus=BMS1) |
| [*maturation of 5.8S rRNA*](http://www.yeastgenome.org/cgi-bin/GO/goTerm.pl?goid=460) | 21 out of 292 genes, 7.2% | 76 out of 7167 genes, 1.1% | 8.01e-10 | [UTP20/YBL004W](http://www.yeastgenome.org/cgi-bin/locus.fpl?locus=UTP20), [MAK5/YBR142W](http://www.yeastgenome.org/cgi-bin/locus.fpl?locus=MAK5), [ENP1/YBR247C](http://www.yeastgenome.org/cgi-bin/locus.fpl?locus=ENP1), [SPB1/YCL054W](http://www.yeastgenome.org/cgi-bin/locus.fpl?locus=SPB1), [NOP14/YDL148C](http://www.yeastgenome.org/cgi-bin/locus.fpl?locus=NOP14), [SAS10/YDL153C](http://www.yeastgenome.org/cgi-bin/locus.fpl?locus=SAS10), [UTP6/YDR449C](http://www.yeastgenome.org/cgi-bin/locus.fpl?locus=UTP6), [UTP7/YER082C](http://www.yeastgenome.org/cgi-bin/locus.fpl?locus=UTP7), [PRP43/YGL120C](http://www.yeastgenome.org/cgi-bin/locus.fpl?locus=PRP43), [MTR4/YJL050W](http://www.yeastgenome.org/cgi-bin/locus.fpl?locus=MTR4), [UTP18/YJL069C](http://www.yeastgenome.org/cgi-bin/locus.fpl?locus=UTP18), [MPP10/YJR002W](http://www.yeastgenome.org/cgi-bin/locus.fpl?locus=MPP10), [MAK11/YKL021C](http://www.yeastgenome.org/cgi-bin/locus.fpl?locus=MAK11), [RPF2/YKR081C](http://www.yeastgenome.org/cgi-bin/locus.fpl?locus=RPF2), [UTP13/YLR222C](http://www.yeastgenome.org/cgi-bin/locus.fpl?locus=UTP13), [GSP1/YLR293C](http://www.yeastgenome.org/cgi-bin/locus.fpl?locus=GSP1), [KRI1/YNL308C](http://www.yeastgenome.org/cgi-bin/locus.fpl?locus=KRI1), [RRP6/YOR001W](http://www.yeastgenome.org/cgi-bin/locus.fpl?locus=RRP6), [PNO1/YOR145C](http://www.yeastgenome.org/cgi-bin/locus.fpl?locus=PNO1), [RRS1/YOR294W](http://www.yeastgenome.org/cgi-bin/locus.fpl?locus=RRS1), [NOP58/YOR310C](http://www.yeastgenome.org/cgi-bin/locus.fpl?locus=NOP58) |
| [*maturation of 5.8S rRNA from tricistronic rRNA transcript (SSU-rRNA, 5.8S rRNA, LSU-rRNA)*](http://www.yeastgenome.org/cgi-bin/GO/goTerm.pl?goid=466) | 21 out of 292 genes, 7.2% | 76 out of 7167 genes, 1.1% | 8.01e-10 | [UTP20/YBL004W](http://www.yeastgenome.org/cgi-bin/locus.fpl?locus=UTP20), [MAK5/YBR142W](http://www.yeastgenome.org/cgi-bin/locus.fpl?locus=MAK5), [ENP1/YBR247C](http://www.yeastgenome.org/cgi-bin/locus.fpl?locus=ENP1), [SPB1/YCL054W](http://www.yeastgenome.org/cgi-bin/locus.fpl?locus=SPB1), [NOP14/YDL148C](http://www.yeastgenome.org/cgi-bin/locus.fpl?locus=NOP14), [SAS10/YDL153C](http://www.yeastgenome.org/cgi-bin/locus.fpl?locus=SAS10), [UTP6/YDR449C](http://www.yeastgenome.org/cgi-bin/locus.fpl?locus=UTP6), [UTP7/YER082C](http://www.yeastgenome.org/cgi-bin/locus.fpl?locus=UTP7), [PRP43/YGL120C](http://www.yeastgenome.org/cgi-bin/locus.fpl?locus=PRP43), [MTR4/YJL050W](http://www.yeastgenome.org/cgi-bin/locus.fpl?locus=MTR4), [UTP18/YJL069C](http://www.yeastgenome.org/cgi-bin/locus.fpl?locus=UTP18), [MPP10/YJR002W](http://www.yeastgenome.org/cgi-bin/locus.fpl?locus=MPP10), [MAK11/YKL021C](http://www.yeastgenome.org/cgi-bin/locus.fpl?locus=MAK11), [RPF2/YKR081C](http://www.yeastgenome.org/cgi-bin/locus.fpl?locus=RPF2), [UTP13/YLR222C](http://www.yeastgenome.org/cgi-bin/locus.fpl?locus=UTP13), [GSP1/YLR293C](http://www.yeastgenome.org/cgi-bin/locus.fpl?locus=GSP1), [KRI1/YNL308C](http://www.yeastgenome.org/cgi-bin/locus.fpl?locus=KRI1), [RRP6/YOR001W](http://www.yeastgenome.org/cgi-bin/locus.fpl?locus=RRP6), [PNO1/YOR145C](http://www.yeastgenome.org/cgi-bin/locus.fpl?locus=PNO1), [RRS1/YOR294W](http://www.yeastgenome.org/cgi-bin/locus.fpl?locus=RRS1), [NOP58/YOR310C](http://www.yeastgenome.org/cgi-bin/locus.fpl?locus=NOP58) |
| [*maturation of SSU-rRNA from tricistronic rRNA transcript (SSU-rRNA, 5.8S rRNA, LSU-rRNA)*](http://www.yeastgenome.org/cgi-bin/GO/goTerm.pl?goid=462) | 22 out of 292 genes, 7.5% | 96 out of 7167 genes, 1.3% | 1.38e-08 | [UTP20/YBL004W](http://www.yeastgenome.org/cgi-bin/locus.fpl?locus=UTP20), [ENP1/YBR247C](http://www.yeastgenome.org/cgi-bin/locus.fpl?locus=ENP1), [NOP14/YDL148C](http://www.yeastgenome.org/cgi-bin/locus.fpl?locus=NOP14), [SAS10/YDL153C](http://www.yeastgenome.org/cgi-bin/locus.fpl?locus=SAS10), [UTP4/YDR324C](http://www.yeastgenome.org/cgi-bin/locus.fpl?locus=UTP4), [UTP5/YDR398W](http://www.yeastgenome.org/cgi-bin/locus.fpl?locus=UTP5), [UTP6/YDR449C](http://www.yeastgenome.org/cgi-bin/locus.fpl?locus=UTP6), [SNU13/YEL026W](http://www.yeastgenome.org/cgi-bin/locus.fpl?locus=SNU13), [UTP7/YER082C](http://www.yeastgenome.org/cgi-bin/locus.fpl?locus=UTP7), [PRP43/YGL120C](http://www.yeastgenome.org/cgi-bin/locus.fpl?locus=PRP43), [NOP7/YGR103W](http://www.yeastgenome.org/cgi-bin/locus.fpl?locus=NOP7), [UTP18/YJL069C](http://www.yeastgenome.org/cgi-bin/locus.fpl?locus=UTP18), [MPP10/YJR002W](http://www.yeastgenome.org/cgi-bin/locus.fpl?locus=MPP10), [UTP13/YLR222C](http://www.yeastgenome.org/cgi-bin/locus.fpl?locus=UTP13), [UTP15/YMR093W](http://www.yeastgenome.org/cgi-bin/locus.fpl?locus=UTP15), [ECM16/YMR128W](http://www.yeastgenome.org/cgi-bin/locus.fpl?locus=ECM16), [KRI1/YNL308C](http://www.yeastgenome.org/cgi-bin/locus.fpl?locus=KRI1), [PNO1/YOR145C](http://www.yeastgenome.org/cgi-bin/locus.fpl?locus=PNO1), [RRS1/YOR294W](http://www.yeastgenome.org/cgi-bin/locus.fpl?locus=RRS1), [NOP58/YOR310C](http://www.yeastgenome.org/cgi-bin/locus.fpl?locus=NOP58), [RRP12/YPL012W](http://www.yeastgenome.org/cgi-bin/locus.fpl?locus=RRP12), [NAN1/YPL126W](http://www.yeastgenome.org/cgi-bin/locus.fpl?locus=NAN1) |
| [*nucleobase, nucleoside, nucleotide and nucleic acid metabolic process*](http://www.yeastgenome.org/cgi-bin/GO/goTerm.pl?goid=6139) | 115 out of 292 genes, 39.4% | 1631 out of 7167 genes, 22.8% | 3.68e-08 | [ADE1/YAR015W](http://www.yeastgenome.org/cgi-bin/locus.fpl?locus=ADE1), [UTP20/YBL004W](http://www.yeastgenome.org/cgi-bin/locus.fpl?locus=UTP20), [NCL1/YBL024W](http://www.yeastgenome.org/cgi-bin/locus.fpl?locus=NCL1), [URA7/YBL039C](http://www.yeastgenome.org/cgi-bin/locus.fpl?locus=URA7), [TOD6/YBL054W](http://www.yeastgenome.org/cgi-bin/locus.fpl?locus=TOD6), [HMT1/YBR034C](http://www.yeastgenome.org/cgi-bin/locus.fpl?locus=HMT1), [POL30/YBR088C](http://www.yeastgenome.org/cgi-bin/locus.fpl?locus=POL30), [TKL2/YBR117C](http://www.yeastgenome.org/cgi-bin/locus.fpl?locus=TKL2), [MAK5/YBR142W](http://www.yeastgenome.org/cgi-bin/locus.fpl?locus=MAK5), [ENP1/YBR247C](http://www.yeastgenome.org/cgi-bin/locus.fpl?locus=ENP1), [SPB1/YCL054W](http://www.yeastgenome.org/cgi-bin/locus.fpl?locus=SPB1), [MATALPHA1/YCR040W](http://www.yeastgenome.org/cgi-bin/locus.fpl?locus=MATALPHA1), [BUD31/YCR063W](http://www.yeastgenome.org/cgi-bin/locus.fpl?locus=BUD31), [MCD1/YDL003W](http://www.yeastgenome.org/cgi-bin/locus.fpl?locus=MCD1), [TSR1/YDL060W](http://www.yeastgenome.org/cgi-bin/locus.fpl?locus=TSR1), [NOP14/YDL148C](http://www.yeastgenome.org/cgi-bin/locus.fpl?locus=NOP14), [SAS10/YDL153C](http://www.yeastgenome.org/cgi-bin/locus.fpl?locus=SAS10), [TRM8/YDL201W](http://www.yeastgenome.org/cgi-bin/locus.fpl?locus=TRM8), [RRP1/YDR087C](http://www.yeastgenome.org/cgi-bin/locus.fpl?locus=RRP1), [SUP35/YDR172W](http://www.yeastgenome.org/cgi-bin/locus.fpl?locus=SUP35), [UTP4/YDR324C](http://www.yeastgenome.org/cgi-bin/locus.fpl?locus=UTP4), [NCB2/YDR397C](http://www.yeastgenome.org/cgi-bin/locus.fpl?locus=NCB2), [UTP5/YDR398W](http://www.yeastgenome.org/cgi-bin/locus.fpl?locus=UTP5), [HPT1/YDR399W](http://www.yeastgenome.org/cgi-bin/locus.fpl?locus=HPT1), [RRP17/YDR412W](http://www.yeastgenome.org/cgi-bin/locus.fpl?locus=RRP17), [DOT1/YDR440W](http://www.yeastgenome.org/cgi-bin/locus.fpl?locus=DOT1), [UTP6/YDR449C](http://www.yeastgenome.org/cgi-bin/locus.fpl?locus=UTP6), [SNU13/YEL026W](http://www.yeastgenome.org/cgi-bin/locus.fpl?locus=SNU13), [PMI40/YER003C](http://www.yeastgenome.org/cgi-bin/locus.fpl?locus=PMI40), [NUG1/YER006W](http://www.yeastgenome.org/cgi-bin/locus.fpl?locus=NUG1), [YER064C](http://www.yeastgenome.org/cgi-bin/locus.fpl?locus=YER064C), [RNR1/YER070W](http://www.yeastgenome.org/cgi-bin/locus.fpl?locus=RNR1), [UTP7/YER082C](http://www.yeastgenome.org/cgi-bin/locus.fpl?locus=UTP7), [DBP3/YGL078C](http://www.yeastgenome.org/cgi-bin/locus.fpl?locus=DBP3), [PRP43/YGL120C](http://www.yeastgenome.org/cgi-bin/locus.fpl?locus=PRP43), [ADE5,7/YGL234W](http://www.yeastgenome.org/cgi-bin/locus.fpl?locus=ADE5), [HXK2/YGL253W](http://www.yeastgenome.org/cgi-bin/locus.fpl?locus=HXK2), [NOP7/YGR103W](http://www.yeastgenome.org/cgi-bin/locus.fpl?locus=NOP7), [ENP2/YGR145W](http://www.yeastgenome.org/cgi-bin/locus.fpl?locus=ENP2), [NSR1/YGR159C](http://www.yeastgenome.org/cgi-bin/locus.fpl?locus=NSR1), [GND2/YGR256W](http://www.yeastgenome.org/cgi-bin/locus.fpl?locus=GND2), [PXR1/YGR280C](http://www.yeastgenome.org/cgi-bin/locus.fpl?locus=PXR1), [GAR1/YHR089C](http://www.yeastgenome.org/cgi-bin/locus.fpl?locus=GAR1), [GND1/YHR183W](http://www.yeastgenome.org/cgi-bin/locus.fpl?locus=GND1), [RIX1/YHR197W](http://www.yeastgenome.org/cgi-bin/locus.fpl?locus=RIX1), [IMD2/YHR216W](http://www.yeastgenome.org/cgi-bin/locus.fpl?locus=IMD2), [RPC17/YJL011C](http://www.yeastgenome.org/cgi-bin/locus.fpl?locus=RPC17), [MTR4/YJL050W](http://www.yeastgenome.org/cgi-bin/locus.fpl?locus=MTR4), [UTP18/YJL069C](http://www.yeastgenome.org/cgi-bin/locus.fpl?locus=UTP18), [SCP160/YJL080C](http://www.yeastgenome.org/cgi-bin/locus.fpl?locus=SCP160), [URA2/YJL130C](http://www.yeastgenome.org/cgi-bin/locus.fpl?locus=URA2), [MPP10/YJR002W](http://www.yeastgenome.org/cgi-bin/locus.fpl?locus=MPP10), [RPA12/YJR063W](http://www.yeastgenome.org/cgi-bin/locus.fpl?locus=RPA12), [URA8/YJR103W](http://www.yeastgenome.org/cgi-bin/locus.fpl?locus=URA8), [MAK11/YKL021C](http://www.yeastgenome.org/cgi-bin/locus.fpl?locus=MAK11), [EBP2/YKL172W](http://www.yeastgenome.org/cgi-bin/locus.fpl?locus=EBP2), [RPF2/YKR081C](http://www.yeastgenome.org/cgi-bin/locus.fpl?locus=RPF2), [SPT8/YLR055C](http://www.yeastgenome.org/cgi-bin/locus.fpl?locus=SPT8), [FRS1/YLR060W](http://www.yeastgenome.org/cgi-bin/locus.fpl?locus=FRS1), [MDN1/YLR106C](http://www.yeastgenome.org/cgi-bin/locus.fpl?locus=MDN1), [STM1/YLR150W](http://www.yeastgenome.org/cgi-bin/locus.fpl?locus=STM1), [CBF5/YLR175W](http://www.yeastgenome.org/cgi-bin/locus.fpl?locus=CBF5), [PWP1/YLR196W](http://www.yeastgenome.org/cgi-bin/locus.fpl?locus=PWP1), [NOP56/YLR197W](http://www.yeastgenome.org/cgi-bin/locus.fpl?locus=NOP56), [UTP13/YLR222C](http://www.yeastgenome.org/cgi-bin/locus.fpl?locus=UTP13), [DBP9/YLR276C](http://www.yeastgenome.org/cgi-bin/locus.fpl?locus=DBP9), [GSP1/YLR293C](http://www.yeastgenome.org/cgi-bin/locus.fpl?locus=GSP1), [ADE13/YLR359W](http://www.yeastgenome.org/cgi-bin/locus.fpl?locus=ADE13), [DUS3/YLR401C](http://www.yeastgenome.org/cgi-bin/locus.fpl?locus=DUS3), [IMD3/YLR432W](http://www.yeastgenome.org/cgi-bin/locus.fpl?locus=IMD3), [FPR4/YLR449W](http://www.yeastgenome.org/cgi-bin/locus.fpl?locus=FPR4), [LEU3/YLR451W](http://www.yeastgenome.org/cgi-bin/locus.fpl?locus=LEU3), [TSA1/YML028W](http://www.yeastgenome.org/cgi-bin/locus.fpl?locus=TSA1), [ERB1/YMR049C](http://www.yeastgenome.org/cgi-bin/locus.fpl?locus=ERB1), [PDS5/YMR076C](http://www.yeastgenome.org/cgi-bin/locus.fpl?locus=PDS5), [UTP15/YMR093W](http://www.yeastgenome.org/cgi-bin/locus.fpl?locus=UTP15), [ADE17/YMR120C](http://www.yeastgenome.org/cgi-bin/locus.fpl?locus=ADE17), [ECM16/YMR128W](http://www.yeastgenome.org/cgi-bin/locus.fpl?locus=ECM16), [NDE1/YMR145C](http://www.yeastgenome.org/cgi-bin/locus.fpl?locus=NDE1), [GUA1/YMR217W](http://www.yeastgenome.org/cgi-bin/locus.fpl?locus=GUA1), [GAS1/YMR307W](http://www.yeastgenome.org/cgi-bin/locus.fpl?locus=GAS1), [NOP2/YNL061W](http://www.yeastgenome.org/cgi-bin/locus.fpl?locus=NOP2), [POL1/YNL102W](http://www.yeastgenome.org/cgi-bin/locus.fpl?locus=POL1), [DBP2/YNL112W](http://www.yeastgenome.org/cgi-bin/locus.fpl?locus=DBP2), [RPC19/YNL113W](http://www.yeastgenome.org/cgi-bin/locus.fpl?locus=RPC19), [AAH1/YNL141W](http://www.yeastgenome.org/cgi-bin/locus.fpl?locus=AAH1), [IPI3/YNL182C](http://www.yeastgenome.org/cgi-bin/locus.fpl?locus=IPI3), [PUS4/YNL292W](http://www.yeastgenome.org/cgi-bin/locus.fpl?locus=PUS4), [KRI1/YNL308C](http://www.yeastgenome.org/cgi-bin/locus.fpl?locus=KRI1), [TRM112/YNR046W](http://www.yeastgenome.org/cgi-bin/locus.fpl?locus=TRM112), [TRM11/YOL124C](http://www.yeastgenome.org/cgi-bin/locus.fpl?locus=TRM11), [CDC33/YOL139C](http://www.yeastgenome.org/cgi-bin/locus.fpl?locus=CDC33), [RRP6/YOR001W](http://www.yeastgenome.org/cgi-bin/locus.fpl?locus=RRP6), [CDC21/YOR074C](http://www.yeastgenome.org/cgi-bin/locus.fpl?locus=CDC21), [RAS1/YOR101W](http://www.yeastgenome.org/cgi-bin/locus.fpl?locus=RAS1), [RPO31/YOR116C](http://www.yeastgenome.org/cgi-bin/locus.fpl?locus=RPO31), [ARP8/YOR141C](http://www.yeastgenome.org/cgi-bin/locus.fpl?locus=ARP8), [PNO1/YOR145C](http://www.yeastgenome.org/cgi-bin/locus.fpl?locus=PNO1), [GLN4/YOR168W](http://www.yeastgenome.org/cgi-bin/locus.fpl?locus=GLN4), [RRS1/YOR294W](http://www.yeastgenome.org/cgi-bin/locus.fpl?locus=RRS1), [NOP58/YOR310C](http://www.yeastgenome.org/cgi-bin/locus.fpl?locus=NOP58), [RPA43/YOR340C](http://www.yeastgenome.org/cgi-bin/locus.fpl?locus=RPA43), [RRP12/YPL012W](http://www.yeastgenome.org/cgi-bin/locus.fpl?locus=RRP12), [NOP4/YPL043W](http://www.yeastgenome.org/cgi-bin/locus.fpl?locus=NOP4), [ELP3/YPL086C](http://www.yeastgenome.org/cgi-bin/locus.fpl?locus=ELP3), [NOG1/YPL093W](http://www.yeastgenome.org/cgi-bin/locus.fpl?locus=NOG1), [NAN1/YPL126W](http://www.yeastgenome.org/cgi-bin/locus.fpl?locus=NAN1), [NOP53/YPL146C](http://www.yeastgenome.org/cgi-bin/locus.fpl?locus=NOP53), [TYW1/YPL207W](http://www.yeastgenome.org/cgi-bin/locus.fpl?locus=TYW1), [NIP7/YPL211W](http://www.yeastgenome.org/cgi-bin/locus.fpl?locus=NIP7), [BMS1/YPL217C](http://www.yeastgenome.org/cgi-bin/locus.fpl?locus=BMS1), [RPA135/YPR010C](http://www.yeastgenome.org/cgi-bin/locus.fpl?locus=RPA135), [CSR2/YPR030W](http://www.yeastgenome.org/cgi-bin/locus.fpl?locus=CSR2), [TKL1/YPR074C](http://www.yeastgenome.org/cgi-bin/locus.fpl?locus=TKL1), [RPC82/YPR190C](http://www.yeastgenome.org/cgi-bin/locus.fpl?locus=RPC82) |
| [*primary metabolic process*](http://www.yeastgenome.org/cgi-bin/GO/goTerm.pl?goid=44238) | 190 out of 292 genes, 65.1% | 3336 out of 7167 genes, 46.5% | 4.03e-08 | [EFB1/YAL003W](http://www.yeastgenome.org/cgi-bin/locus.fpl?locus=EFB1), [PMT2/YAL023C](http://www.yeastgenome.org/cgi-bin/locus.fpl?locus=PMT2), [FUN12/YAL035W](http://www.yeastgenome.org/cgi-bin/locus.fpl?locus=FUN12), [GDH3/YAL062W](http://www.yeastgenome.org/cgi-bin/locus.fpl?locus=GDH3), [ADE1/YAR015W](http://www.yeastgenome.org/cgi-bin/locus.fpl?locus=ADE1), [UTP20/YBL004W](http://www.yeastgenome.org/cgi-bin/locus.fpl?locus=UTP20), [NCL1/YBL024W](http://www.yeastgenome.org/cgi-bin/locus.fpl?locus=NCL1), [URA7/YBL039C](http://www.yeastgenome.org/cgi-bin/locus.fpl?locus=URA7), [TOD6/YBL054W](http://www.yeastgenome.org/cgi-bin/locus.fpl?locus=TOD6), [CDS1/YBR029C](http://www.yeastgenome.org/cgi-bin/locus.fpl?locus=CDS1), [HMT1/YBR034C](http://www.yeastgenome.org/cgi-bin/locus.fpl?locus=HMT1), [RPG1/YBR079C](http://www.yeastgenome.org/cgi-bin/locus.fpl?locus=RPG1), [POL30/YBR088C](http://www.yeastgenome.org/cgi-bin/locus.fpl?locus=POL30), [TKL2/YBR117C](http://www.yeastgenome.org/cgi-bin/locus.fpl?locus=TKL2), [MAK5/YBR142W](http://www.yeastgenome.org/cgi-bin/locus.fpl?locus=MAK5), [CNS1/YBR155W](http://www.yeastgenome.org/cgi-bin/locus.fpl?locus=CNS1), [ENP1/YBR247C](http://www.yeastgenome.org/cgi-bin/locus.fpl?locus=ENP1), [SHM1/YBR263W](http://www.yeastgenome.org/cgi-bin/locus.fpl?locus=SHM1), [YCL003W](http://www.yeastgenome.org/cgi-bin/locus.fpl?locus=YCL003W), [KCC4/YCL024W](http://www.yeastgenome.org/cgi-bin/locus.fpl?locus=KCC4), [SRO9/YCL037C](http://www.yeastgenome.org/cgi-bin/locus.fpl?locus=SRO9), [SPB1/YCL054W](http://www.yeastgenome.org/cgi-bin/locus.fpl?locus=SPB1), [CWH43/YCR017C](http://www.yeastgenome.org/cgi-bin/locus.fpl?locus=CWH43), [FEN1/YCR034W](http://www.yeastgenome.org/cgi-bin/locus.fpl?locus=FEN1), [MATALPHA1/YCR040W](http://www.yeastgenome.org/cgi-bin/locus.fpl?locus=MATALPHA1), [IMG1/YCR046C](http://www.yeastgenome.org/cgi-bin/locus.fpl?locus=IMG1), [BUD31/YCR063W](http://www.yeastgenome.org/cgi-bin/locus.fpl?locus=BUD31), [MCD1/YDL003W](http://www.yeastgenome.org/cgi-bin/locus.fpl?locus=MCD1), [TSR1/YDL060W](http://www.yeastgenome.org/cgi-bin/locus.fpl?locus=TSR1), [NOP14/YDL148C](http://www.yeastgenome.org/cgi-bin/locus.fpl?locus=NOP14), [SAS10/YDL153C](http://www.yeastgenome.org/cgi-bin/locus.fpl?locus=SAS10), [TRM8/YDL201W](http://www.yeastgenome.org/cgi-bin/locus.fpl?locus=TRM8), [RRP1/YDR087C](http://www.yeastgenome.org/cgi-bin/locus.fpl?locus=RRP1), [MKC7/YDR144C](http://www.yeastgenome.org/cgi-bin/locus.fpl?locus=MKC7), [SUP35/YDR172W](http://www.yeastgenome.org/cgi-bin/locus.fpl?locus=SUP35), [LYS4/YDR234W](http://www.yeastgenome.org/cgi-bin/locus.fpl?locus=LYS4), [ASP1/YDR321W](http://www.yeastgenome.org/cgi-bin/locus.fpl?locus=ASP1), [UTP4/YDR324C](http://www.yeastgenome.org/cgi-bin/locus.fpl?locus=UTP4), [NCB2/YDR397C](http://www.yeastgenome.org/cgi-bin/locus.fpl?locus=NCB2), [UTP5/YDR398W](http://www.yeastgenome.org/cgi-bin/locus.fpl?locus=UTP5), [HPT1/YDR399W](http://www.yeastgenome.org/cgi-bin/locus.fpl?locus=HPT1), [RRP17/YDR412W](http://www.yeastgenome.org/cgi-bin/locus.fpl?locus=RRP17), [TIF35/YDR429C](http://www.yeastgenome.org/cgi-bin/locus.fpl?locus=TIF35), [DOT1/YDR440W](http://www.yeastgenome.org/cgi-bin/locus.fpl?locus=DOT1), [UTP6/YDR449C](http://www.yeastgenome.org/cgi-bin/locus.fpl?locus=UTP6), [RMT2/YDR465C](http://www.yeastgenome.org/cgi-bin/locus.fpl?locus=RMT2), [PUF6/YDR496C](http://www.yeastgenome.org/cgi-bin/locus.fpl?locus=PUF6), [SNU13/YEL026W](http://www.yeastgenome.org/cgi-bin/locus.fpl?locus=SNU13), [ECM10/YEL030W](http://www.yeastgenome.org/cgi-bin/locus.fpl?locus=ECM10), [HYP2/YEL034W](http://www.yeastgenome.org/cgi-bin/locus.fpl?locus=HYP2), [GDA1/YEL042W](http://www.yeastgenome.org/cgi-bin/locus.fpl?locus=GDA1), [HPA3/YEL066W](http://www.yeastgenome.org/cgi-bin/locus.fpl?locus=HPA3), [PMI40/YER003C](http://www.yeastgenome.org/cgi-bin/locus.fpl?locus=PMI40), [NUG1/YER006W](http://www.yeastgenome.org/cgi-bin/locus.fpl?locus=NUG1), [SAH1/YER043C](http://www.yeastgenome.org/cgi-bin/locus.fpl?locus=SAH1), [YER064C](http://www.yeastgenome.org/cgi-bin/locus.fpl?locus=YER064C), [RNR1/YER070W](http://www.yeastgenome.org/cgi-bin/locus.fpl?locus=RNR1), [UTP7/YER082C](http://www.yeastgenome.org/cgi-bin/locus.fpl?locus=UTP7), [BUD27/YFL023W](http://www.yeastgenome.org/cgi-bin/locus.fpl?locus=BUD27), [OLE1/YGL055W](http://www.yeastgenome.org/cgi-bin/locus.fpl?locus=OLE1), [DBP3/YGL078C](http://www.yeastgenome.org/cgi-bin/locus.fpl?locus=DBP3), [PRP43/YGL120C](http://www.yeastgenome.org/cgi-bin/locus.fpl?locus=PRP43), [ADE5,7/YGL234W](http://www.yeastgenome.org/cgi-bin/locus.fpl?locus=ADE5), [HXK2/YGL253W](http://www.yeastgenome.org/cgi-bin/locus.fpl?locus=HXK2), [YGR054W](http://www.yeastgenome.org/cgi-bin/locus.fpl?locus=YGR054W), [ERG25/YGR060W](http://www.yeastgenome.org/cgi-bin/locus.fpl?locus=ERG25), [GCD2/YGR083C](http://www.yeastgenome.org/cgi-bin/locus.fpl?locus=GCD2), [PDC6/YGR087C](http://www.yeastgenome.org/cgi-bin/locus.fpl?locus=PDC6), [NOP7/YGR103W](http://www.yeastgenome.org/cgi-bin/locus.fpl?locus=NOP7), [PPT1/YGR123C](http://www.yeastgenome.org/cgi-bin/locus.fpl?locus=PPT1), [ENP2/YGR145W](http://www.yeastgenome.org/cgi-bin/locus.fpl?locus=ENP2), [CYS4/YGR155W](http://www.yeastgenome.org/cgi-bin/locus.fpl?locus=CYS4), [CHO2/YGR157W](http://www.yeastgenome.org/cgi-bin/locus.fpl?locus=CHO2), [NSR1/YGR159C](http://www.yeastgenome.org/cgi-bin/locus.fpl?locus=NSR1), [TIF4631/YGR162W](http://www.yeastgenome.org/cgi-bin/locus.fpl?locus=TIF4631), [CRH1/YGR189C](http://www.yeastgenome.org/cgi-bin/locus.fpl?locus=CRH1), [PFK1/YGR240C](http://www.yeastgenome.org/cgi-bin/locus.fpl?locus=PFK1), [GND2/YGR256W](http://www.yeastgenome.org/cgi-bin/locus.fpl?locus=GND2), [PXR1/YGR280C](http://www.yeastgenome.org/cgi-bin/locus.fpl?locus=PXR1), [ZUO1/YGR285C](http://www.yeastgenome.org/cgi-bin/locus.fpl?locus=ZUO1), [PRS3/YHL011C](http://www.yeastgenome.org/cgi-bin/locus.fpl?locus=PRS3), [NCP1/YHR042W](http://www.yeastgenome.org/cgi-bin/locus.fpl?locus=NCP1), [CIC1/YHR052W](http://www.yeastgenome.org/cgi-bin/locus.fpl?locus=CIC1), [SSZ1/YHR064C](http://www.yeastgenome.org/cgi-bin/locus.fpl?locus=SSZ1), [DYS1/YHR068W](http://www.yeastgenome.org/cgi-bin/locus.fpl?locus=DYS1), [GAR1/YHR089C](http://www.yeastgenome.org/cgi-bin/locus.fpl?locus=GAR1), [GND1/YHR183W](http://www.yeastgenome.org/cgi-bin/locus.fpl?locus=GND1), [RIX1/YHR197W](http://www.yeastgenome.org/cgi-bin/locus.fpl?locus=RIX1), [IMD2/YHR216W](http://www.yeastgenome.org/cgi-bin/locus.fpl?locus=IMD2), [FAA3/YIL009W](http://www.yeastgenome.org/cgi-bin/locus.fpl?locus=FAA3), [CCT2/YIL142W](http://www.yeastgenome.org/cgi-bin/locus.fpl?locus=CCT2), [CCT8/YJL008C](http://www.yeastgenome.org/cgi-bin/locus.fpl?locus=CCT8), [RPC17/YJL011C](http://www.yeastgenome.org/cgi-bin/locus.fpl?locus=RPC17), [MTR4/YJL050W](http://www.yeastgenome.org/cgi-bin/locus.fpl?locus=MTR4), [UTP18/YJL069C](http://www.yeastgenome.org/cgi-bin/locus.fpl?locus=UTP18), [SCP160/YJL080C](http://www.yeastgenome.org/cgi-bin/locus.fpl?locus=SCP160), [URA2/YJL130C](http://www.yeastgenome.org/cgi-bin/locus.fpl?locus=URA2), [CPS1/YJL172W](http://www.yeastgenome.org/cgi-bin/locus.fpl?locus=CPS1), [MNN11/YJL183W](http://www.yeastgenome.org/cgi-bin/locus.fpl?locus=MNN11), [MPP10/YJR002W](http://www.yeastgenome.org/cgi-bin/locus.fpl?locus=MPP10), [ANB1/YJR047C](http://www.yeastgenome.org/cgi-bin/locus.fpl?locus=ANB1), [RPA12/YJR063W](http://www.yeastgenome.org/cgi-bin/locus.fpl?locus=RPA12), [LIA1/YJR070C](http://www.yeastgenome.org/cgi-bin/locus.fpl?locus=LIA1), [URA8/YJR103W](http://www.yeastgenome.org/cgi-bin/locus.fpl?locus=URA8), [BAT2/YJR148W](http://www.yeastgenome.org/cgi-bin/locus.fpl?locus=BAT2), [AUR1/YKL004W](http://www.yeastgenome.org/cgi-bin/locus.fpl?locus=AUR1), [MAK11/YKL021C](http://www.yeastgenome.org/cgi-bin/locus.fpl?locus=MAK11), [TEF4/YKL081W](http://www.yeastgenome.org/cgi-bin/locus.fpl?locus=TEF4), [EBP2/YKL172W](http://www.yeastgenome.org/cgi-bin/locus.fpl?locus=EBP2), [DPH2/YKL191W](http://www.yeastgenome.org/cgi-bin/locus.fpl?locus=DPH2), [FOX2/YKR009C](http://www.yeastgenome.org/cgi-bin/locus.fpl?locus=FOX2), [GAP1/YKR039W](http://www.yeastgenome.org/cgi-bin/locus.fpl?locus=GAP1), [RPF2/YKR081C](http://www.yeastgenome.org/cgi-bin/locus.fpl?locus=RPF2), [RPL8B/YLL045C](http://www.yeastgenome.org/cgi-bin/locus.fpl?locus=RPL8B), [SPT8/YLR055C](http://www.yeastgenome.org/cgi-bin/locus.fpl?locus=SPT8), [ERG3/YLR056W](http://www.yeastgenome.org/cgi-bin/locus.fpl?locus=ERG3), [FRS1/YLR060W](http://www.yeastgenome.org/cgi-bin/locus.fpl?locus=FRS1), [RPL22A/YLR061W](http://www.yeastgenome.org/cgi-bin/locus.fpl?locus=RPL22A), [MDN1/YLR106C](http://www.yeastgenome.org/cgi-bin/locus.fpl?locus=MDN1), [PDC5/YLR134W](http://www.yeastgenome.org/cgi-bin/locus.fpl?locus=PDC5), [PUT1/YLR142W](http://www.yeastgenome.org/cgi-bin/locus.fpl?locus=PUT1), [STM1/YLR150W](http://www.yeastgenome.org/cgi-bin/locus.fpl?locus=STM1), [DPH5/YLR172C](http://www.yeastgenome.org/cgi-bin/locus.fpl?locus=DPH5), [CBF5/YLR175W](http://www.yeastgenome.org/cgi-bin/locus.fpl?locus=CBF5), [PWP1/YLR196W](http://www.yeastgenome.org/cgi-bin/locus.fpl?locus=PWP1), [NOP56/YLR197W](http://www.yeastgenome.org/cgi-bin/locus.fpl?locus=NOP56), [UTP13/YLR222C](http://www.yeastgenome.org/cgi-bin/locus.fpl?locus=UTP13), [DBP9/YLR276C](http://www.yeastgenome.org/cgi-bin/locus.fpl?locus=DBP9), [GSP1/YLR293C](http://www.yeastgenome.org/cgi-bin/locus.fpl?locus=GSP1), [ADE13/YLR359W](http://www.yeastgenome.org/cgi-bin/locus.fpl?locus=ADE13), [CSR1/YLR380W](http://www.yeastgenome.org/cgi-bin/locus.fpl?locus=CSR1), [DUS3/YLR401C](http://www.yeastgenome.org/cgi-bin/locus.fpl?locus=DUS3), [IMD3/YLR432W](http://www.yeastgenome.org/cgi-bin/locus.fpl?locus=IMD3), [FPR4/YLR449W](http://www.yeastgenome.org/cgi-bin/locus.fpl?locus=FPR4), [LEU3/YLR451W](http://www.yeastgenome.org/cgi-bin/locus.fpl?locus=LEU3), [ERG6/YML008C](http://www.yeastgenome.org/cgi-bin/locus.fpl?locus=ERG6), [TSA1/YML028W](http://www.yeastgenome.org/cgi-bin/locus.fpl?locus=TSA1), [ERB1/YMR049C](http://www.yeastgenome.org/cgi-bin/locus.fpl?locus=ERB1), [PDS5/YMR076C](http://www.yeastgenome.org/cgi-bin/locus.fpl?locus=PDS5), [UTP15/YMR093W](http://www.yeastgenome.org/cgi-bin/locus.fpl?locus=UTP15), [ADE17/YMR120C](http://www.yeastgenome.org/cgi-bin/locus.fpl?locus=ADE17), [ECM16/YMR128W](http://www.yeastgenome.org/cgi-bin/locus.fpl?locus=ECM16), [NDE1/YMR145C](http://www.yeastgenome.org/cgi-bin/locus.fpl?locus=NDE1), [GUA1/YMR217W](http://www.yeastgenome.org/cgi-bin/locus.fpl?locus=GUA1), [GAS1/YMR307W](http://www.yeastgenome.org/cgi-bin/locus.fpl?locus=GAS1), [NIP1/YMR309C](http://www.yeastgenome.org/cgi-bin/locus.fpl?locus=NIP1), [HEF3/YNL014W](http://www.yeastgenome.org/cgi-bin/locus.fpl?locus=HEF3), [NOP2/YNL061W](http://www.yeastgenome.org/cgi-bin/locus.fpl?locus=NOP2), [POL1/YNL102W](http://www.yeastgenome.org/cgi-bin/locus.fpl?locus=POL1), [DBP2/YNL112W](http://www.yeastgenome.org/cgi-bin/locus.fpl?locus=DBP2), [RPC19/YNL113W](http://www.yeastgenome.org/cgi-bin/locus.fpl?locus=RPC19), [AAH1/YNL141W](http://www.yeastgenome.org/cgi-bin/locus.fpl?locus=AAH1), [IPI3/YNL182C](http://www.yeastgenome.org/cgi-bin/locus.fpl?locus=IPI3), [PUS4/YNL292W](http://www.yeastgenome.org/cgi-bin/locus.fpl?locus=PUS4), [KRI1/YNL308C](http://www.yeastgenome.org/cgi-bin/locus.fpl?locus=KRI1), [ACC1/YNR016C](http://www.yeastgenome.org/cgi-bin/locus.fpl?locus=ACC1), [MVD1/YNR043W](http://www.yeastgenome.org/cgi-bin/locus.fpl?locus=MVD1), [TRM112/YNR046W](http://www.yeastgenome.org/cgi-bin/locus.fpl?locus=TRM112), [LYS9/YNR050C](http://www.yeastgenome.org/cgi-bin/locus.fpl?locus=LYS9), [TRM11/YOL124C](http://www.yeastgenome.org/cgi-bin/locus.fpl?locus=TRM11), [CDC33/YOL139C](http://www.yeastgenome.org/cgi-bin/locus.fpl?locus=CDC33), [RRP6/YOR001W](http://www.yeastgenome.org/cgi-bin/locus.fpl?locus=RRP6), [CDC21/YOR074C](http://www.yeastgenome.org/cgi-bin/locus.fpl?locus=CDC21), [RAS1/YOR101W](http://www.yeastgenome.org/cgi-bin/locus.fpl?locus=RAS1), [RPO31/YOR116C](http://www.yeastgenome.org/cgi-bin/locus.fpl?locus=RPO31), [ARP8/YOR141C](http://www.yeastgenome.org/cgi-bin/locus.fpl?locus=ARP8), [PNO1/YOR145C](http://www.yeastgenome.org/cgi-bin/locus.fpl?locus=PNO1), [GLN4/YOR168W](http://www.yeastgenome.org/cgi-bin/locus.fpl?locus=GLN4), [RRS1/YOR294W](http://www.yeastgenome.org/cgi-bin/locus.fpl?locus=RRS1), [NOP58/YOR310C](http://www.yeastgenome.org/cgi-bin/locus.fpl?locus=NOP58), [RPA43/YOR340C](http://www.yeastgenome.org/cgi-bin/locus.fpl?locus=RPA43), [PUT4/YOR348C](http://www.yeastgenome.org/cgi-bin/locus.fpl?locus=PUT4), [GDH1/YOR375C](http://www.yeastgenome.org/cgi-bin/locus.fpl?locus=GDH1), [RRP12/YPL012W](http://www.yeastgenome.org/cgi-bin/locus.fpl?locus=RRP12), [NOP4/YPL043W](http://www.yeastgenome.org/cgi-bin/locus.fpl?locus=NOP4), [ELP3/YPL086C](http://www.yeastgenome.org/cgi-bin/locus.fpl?locus=ELP3), [NOG1/YPL093W](http://www.yeastgenome.org/cgi-bin/locus.fpl?locus=NOG1), [CAR1/YPL111W](http://www.yeastgenome.org/cgi-bin/locus.fpl?locus=CAR1), [NAN1/YPL126W](http://www.yeastgenome.org/cgi-bin/locus.fpl?locus=NAN1), [NOP53/YPL146C](http://www.yeastgenome.org/cgi-bin/locus.fpl?locus=NOP53), [RPL7B/YPL198W](http://www.yeastgenome.org/cgi-bin/locus.fpl?locus=RPL7B), [TYW1/YPL207W](http://www.yeastgenome.org/cgi-bin/locus.fpl?locus=TYW1), [NIP7/YPL211W](http://www.yeastgenome.org/cgi-bin/locus.fpl?locus=NIP7), [BMS1/YPL217C](http://www.yeastgenome.org/cgi-bin/locus.fpl?locus=BMS1), [RPA135/YPR010C](http://www.yeastgenome.org/cgi-bin/locus.fpl?locus=RPA135), [CSR2/YPR030W](http://www.yeastgenome.org/cgi-bin/locus.fpl?locus=CSR2), [GLN1/YPR035W](http://www.yeastgenome.org/cgi-bin/locus.fpl?locus=GLN1), [TKL1/YPR074C](http://www.yeastgenome.org/cgi-bin/locus.fpl?locus=TKL1), [TIF3/YPR163C](http://www.yeastgenome.org/cgi-bin/locus.fpl?locus=TIF3), [RPC82/YPR190C](http://www.yeastgenome.org/cgi-bin/locus.fpl?locus=RPC82) |
| [*maturation of SSU-rRNA*](http://www.yeastgenome.org/cgi-bin/GO/goTerm.pl?goid=30490) | 22 out of 292 genes, 7.5% | 102 out of 7167 genes, 1.4% | 4.97e-08 | [UTP20/YBL004W](http://www.yeastgenome.org/cgi-bin/locus.fpl?locus=UTP20), [ENP1/YBR247C](http://www.yeastgenome.org/cgi-bin/locus.fpl?locus=ENP1), [NOP14/YDL148C](http://www.yeastgenome.org/cgi-bin/locus.fpl?locus=NOP14), [SAS10/YDL153C](http://www.yeastgenome.org/cgi-bin/locus.fpl?locus=SAS10), [UTP4/YDR324C](http://www.yeastgenome.org/cgi-bin/locus.fpl?locus=UTP4), [UTP5/YDR398W](http://www.yeastgenome.org/cgi-bin/locus.fpl?locus=UTP5), [UTP6/YDR449C](http://www.yeastgenome.org/cgi-bin/locus.fpl?locus=UTP6), [SNU13/YEL026W](http://www.yeastgenome.org/cgi-bin/locus.fpl?locus=SNU13), [UTP7/YER082C](http://www.yeastgenome.org/cgi-bin/locus.fpl?locus=UTP7), [PRP43/YGL120C](http://www.yeastgenome.org/cgi-bin/locus.fpl?locus=PRP43), [NOP7/YGR103W](http://www.yeastgenome.org/cgi-bin/locus.fpl?locus=NOP7), [UTP18/YJL069C](http://www.yeastgenome.org/cgi-bin/locus.fpl?locus=UTP18), [MPP10/YJR002W](http://www.yeastgenome.org/cgi-bin/locus.fpl?locus=MPP10), [UTP13/YLR222C](http://www.yeastgenome.org/cgi-bin/locus.fpl?locus=UTP13), [UTP15/YMR093W](http://www.yeastgenome.org/cgi-bin/locus.fpl?locus=UTP15), [ECM16/YMR128W](http://www.yeastgenome.org/cgi-bin/locus.fpl?locus=ECM16), [KRI1/YNL308C](http://www.yeastgenome.org/cgi-bin/locus.fpl?locus=KRI1), [PNO1/YOR145C](http://www.yeastgenome.org/cgi-bin/locus.fpl?locus=PNO1), [RRS1/YOR294W](http://www.yeastgenome.org/cgi-bin/locus.fpl?locus=RRS1), [NOP58/YOR310C](http://www.yeastgenome.org/cgi-bin/locus.fpl?locus=NOP58), [RRP12/YPL012W](http://www.yeastgenome.org/cgi-bin/locus.fpl?locus=RRP12), [NAN1/YPL126W](http://www.yeastgenome.org/cgi-bin/locus.fpl?locus=NAN1) |
| [*ribosome localization*](http://www.yeastgenome.org/cgi-bin/GO/goTerm.pl?goid=33750) | 13 out of 292 genes, 4.5% | 32 out of 7167 genes, 0.4% | 7.46e-08 | [NUG1/YER006W](http://www.yeastgenome.org/cgi-bin/locus.fpl?locus=NUG1), [ARB1/YER036C](http://www.yeastgenome.org/cgi-bin/locus.fpl?locus=ARB1), [LSG1/YGL099W](http://www.yeastgenome.org/cgi-bin/locus.fpl?locus=LSG1), [SDA1/YGR245C](http://www.yeastgenome.org/cgi-bin/locus.fpl?locus=SDA1), [NMD3/YHR170W](http://www.yeastgenome.org/cgi-bin/locus.fpl?locus=NMD3), [RIX1/YHR197W](http://www.yeastgenome.org/cgi-bin/locus.fpl?locus=RIX1), [LTV1/YKL143W](http://www.yeastgenome.org/cgi-bin/locus.fpl?locus=LTV1), [RIX7/YLL034C](http://www.yeastgenome.org/cgi-bin/locus.fpl?locus=RIX7), [NOG2/YNR053C](http://www.yeastgenome.org/cgi-bin/locus.fpl?locus=NOG2), [NOC2/YOR206W](http://www.yeastgenome.org/cgi-bin/locus.fpl?locus=NOC2), [RRS1/YOR294W](http://www.yeastgenome.org/cgi-bin/locus.fpl?locus=RRS1), [NOG1/YPL093W](http://www.yeastgenome.org/cgi-bin/locus.fpl?locus=NOG1), [NOP53/YPL146C](http://www.yeastgenome.org/cgi-bin/locus.fpl?locus=NOP53) |
| [*establishment of ribosome localization*](http://www.yeastgenome.org/cgi-bin/GO/goTerm.pl?goid=33753) | 13 out of 292 genes, 4.5% | 32 out of 7167 genes, 0.4% | 7.46e-08 | [NUG1/YER006W](http://www.yeastgenome.org/cgi-bin/locus.fpl?locus=NUG1), [ARB1/YER036C](http://www.yeastgenome.org/cgi-bin/locus.fpl?locus=ARB1), [LSG1/YGL099W](http://www.yeastgenome.org/cgi-bin/locus.fpl?locus=LSG1), [SDA1/YGR245C](http://www.yeastgenome.org/cgi-bin/locus.fpl?locus=SDA1), [NMD3/YHR170W](http://www.yeastgenome.org/cgi-bin/locus.fpl?locus=NMD3), [RIX1/YHR197W](http://www.yeastgenome.org/cgi-bin/locus.fpl?locus=RIX1), [LTV1/YKL143W](http://www.yeastgenome.org/cgi-bin/locus.fpl?locus=LTV1), [RIX7/YLL034C](http://www.yeastgenome.org/cgi-bin/locus.fpl?locus=RIX7), [NOG2/YNR053C](http://www.yeastgenome.org/cgi-bin/locus.fpl?locus=NOG2), [NOC2/YOR206W](http://www.yeastgenome.org/cgi-bin/locus.fpl?locus=NOC2), [RRS1/YOR294W](http://www.yeastgenome.org/cgi-bin/locus.fpl?locus=RRS1), [NOG1/YPL093W](http://www.yeastgenome.org/cgi-bin/locus.fpl?locus=NOG1), [NOP53/YPL146C](http://www.yeastgenome.org/cgi-bin/locus.fpl?locus=NOP53) |
| [*ribosomal subunit export from nucleus*](http://www.yeastgenome.org/cgi-bin/GO/goTerm.pl?goid=54) | 13 out of 292 genes, 4.5% | 32 out of 7167 genes, 0.4% | 7.46e-08 | [NUG1/YER006W](http://www.yeastgenome.org/cgi-bin/locus.fpl?locus=NUG1), [ARB1/YER036C](http://www.yeastgenome.org/cgi-bin/locus.fpl?locus=ARB1), [LSG1/YGL099W](http://www.yeastgenome.org/cgi-bin/locus.fpl?locus=LSG1), [SDA1/YGR245C](http://www.yeastgenome.org/cgi-bin/locus.fpl?locus=SDA1), [NMD3/YHR170W](http://www.yeastgenome.org/cgi-bin/locus.fpl?locus=NMD3), [RIX1/YHR197W](http://www.yeastgenome.org/cgi-bin/locus.fpl?locus=RIX1), [LTV1/YKL143W](http://www.yeastgenome.org/cgi-bin/locus.fpl?locus=LTV1), [RIX7/YLL034C](http://www.yeastgenome.org/cgi-bin/locus.fpl?locus=RIX7), [NOG2/YNR053C](http://www.yeastgenome.org/cgi-bin/locus.fpl?locus=NOG2), [NOC2/YOR206W](http://www.yeastgenome.org/cgi-bin/locus.fpl?locus=NOC2), [RRS1/YOR294W](http://www.yeastgenome.org/cgi-bin/locus.fpl?locus=RRS1), [NOG1/YPL093W](http://www.yeastgenome.org/cgi-bin/locus.fpl?locus=NOG1), [NOP53/YPL146C](http://www.yeastgenome.org/cgi-bin/locus.fpl?locus=NOP53) |
| [*small molecule metabolic process*](http://www.yeastgenome.org/cgi-bin/GO/goTerm.pl?goid=44281) | 71 out of 292 genes, 24.3% | 839 out of 7167 genes, 11.7% | 4.08e-07 | [GDH3/YAL062W](http://www.yeastgenome.org/cgi-bin/locus.fpl?locus=GDH3), [ADE1/YAR015W](http://www.yeastgenome.org/cgi-bin/locus.fpl?locus=ADE1), [NCL1/YBL024W](http://www.yeastgenome.org/cgi-bin/locus.fpl?locus=NCL1), [URA7/YBL039C](http://www.yeastgenome.org/cgi-bin/locus.fpl?locus=URA7), [HMT1/YBR034C](http://www.yeastgenome.org/cgi-bin/locus.fpl?locus=HMT1), [PHO3/YBR092C](http://www.yeastgenome.org/cgi-bin/locus.fpl?locus=PHO3), [TKL2/YBR117C](http://www.yeastgenome.org/cgi-bin/locus.fpl?locus=TKL2), [DUR1,2/YBR208C](http://www.yeastgenome.org/cgi-bin/locus.fpl?locus=DUR1), [SHM1/YBR263W](http://www.yeastgenome.org/cgi-bin/locus.fpl?locus=SHM1), [SPB1/YCL054W](http://www.yeastgenome.org/cgi-bin/locus.fpl?locus=SPB1), [FEN1/YCR034W](http://www.yeastgenome.org/cgi-bin/locus.fpl?locus=FEN1), [TRM8/YDL201W](http://www.yeastgenome.org/cgi-bin/locus.fpl?locus=TRM8), [LYS4/YDR234W](http://www.yeastgenome.org/cgi-bin/locus.fpl?locus=LYS4), [ASP1/YDR321W](http://www.yeastgenome.org/cgi-bin/locus.fpl?locus=ASP1), [HPT1/YDR399W](http://www.yeastgenome.org/cgi-bin/locus.fpl?locus=HPT1), [DOT1/YDR440W](http://www.yeastgenome.org/cgi-bin/locus.fpl?locus=DOT1), [RMT2/YDR465C](http://www.yeastgenome.org/cgi-bin/locus.fpl?locus=RMT2), [HPA3/YEL066W](http://www.yeastgenome.org/cgi-bin/locus.fpl?locus=HPA3), [PMI40/YER003C](http://www.yeastgenome.org/cgi-bin/locus.fpl?locus=PMI40), [SAH1/YER043C](http://www.yeastgenome.org/cgi-bin/locus.fpl?locus=SAH1), [RNR1/YER070W](http://www.yeastgenome.org/cgi-bin/locus.fpl?locus=RNR1), [OLE1/YGL055W](http://www.yeastgenome.org/cgi-bin/locus.fpl?locus=OLE1), [ADE5,7/YGL234W](http://www.yeastgenome.org/cgi-bin/locus.fpl?locus=ADE5), [HXK2/YGL253W](http://www.yeastgenome.org/cgi-bin/locus.fpl?locus=HXK2), [ERG25/YGR060W](http://www.yeastgenome.org/cgi-bin/locus.fpl?locus=ERG25), [PDC6/YGR087C](http://www.yeastgenome.org/cgi-bin/locus.fpl?locus=PDC6), [CYS4/YGR155W](http://www.yeastgenome.org/cgi-bin/locus.fpl?locus=CYS4), [CHO2/YGR157W](http://www.yeastgenome.org/cgi-bin/locus.fpl?locus=CHO2), [PFK1/YGR240C](http://www.yeastgenome.org/cgi-bin/locus.fpl?locus=PFK1), [GND2/YGR256W](http://www.yeastgenome.org/cgi-bin/locus.fpl?locus=GND2), [PRS3/YHL011C](http://www.yeastgenome.org/cgi-bin/locus.fpl?locus=PRS3), [DUR3/YHL016C](http://www.yeastgenome.org/cgi-bin/locus.fpl?locus=DUR3), [NCP1/YHR042W](http://www.yeastgenome.org/cgi-bin/locus.fpl?locus=NCP1), [DYS1/YHR068W](http://www.yeastgenome.org/cgi-bin/locus.fpl?locus=DYS1), [GND1/YHR183W](http://www.yeastgenome.org/cgi-bin/locus.fpl?locus=GND1), [IMD2/YHR216W](http://www.yeastgenome.org/cgi-bin/locus.fpl?locus=IMD2), [FAA3/YIL009W](http://www.yeastgenome.org/cgi-bin/locus.fpl?locus=FAA3), [URA2/YJL130C](http://www.yeastgenome.org/cgi-bin/locus.fpl?locus=URA2), [LIA1/YJR070C](http://www.yeastgenome.org/cgi-bin/locus.fpl?locus=LIA1), [URA8/YJR103W](http://www.yeastgenome.org/cgi-bin/locus.fpl?locus=URA8), [BAT2/YJR148W](http://www.yeastgenome.org/cgi-bin/locus.fpl?locus=BAT2), [FOX2/YKR009C](http://www.yeastgenome.org/cgi-bin/locus.fpl?locus=FOX2), [GAP1/YKR039W](http://www.yeastgenome.org/cgi-bin/locus.fpl?locus=GAP1), [ERG3/YLR056W](http://www.yeastgenome.org/cgi-bin/locus.fpl?locus=ERG3), [FRS1/YLR060W](http://www.yeastgenome.org/cgi-bin/locus.fpl?locus=FRS1), [PDC5/YLR134W](http://www.yeastgenome.org/cgi-bin/locus.fpl?locus=PDC5), [PUT1/YLR142W](http://www.yeastgenome.org/cgi-bin/locus.fpl?locus=PUT1), [ADE13/YLR359W](http://www.yeastgenome.org/cgi-bin/locus.fpl?locus=ADE13), [CSR1/YLR380W](http://www.yeastgenome.org/cgi-bin/locus.fpl?locus=CSR1), [IMD3/YLR432W](http://www.yeastgenome.org/cgi-bin/locus.fpl?locus=IMD3), [FPR4/YLR449W](http://www.yeastgenome.org/cgi-bin/locus.fpl?locus=FPR4), [LEU3/YLR451W](http://www.yeastgenome.org/cgi-bin/locus.fpl?locus=LEU3), [ERG6/YML008C](http://www.yeastgenome.org/cgi-bin/locus.fpl?locus=ERG6), [ADE17/YMR120C](http://www.yeastgenome.org/cgi-bin/locus.fpl?locus=ADE17), [NDE1/YMR145C](http://www.yeastgenome.org/cgi-bin/locus.fpl?locus=NDE1), [GUA1/YMR217W](http://www.yeastgenome.org/cgi-bin/locus.fpl?locus=GUA1), [AAH1/YNL141W](http://www.yeastgenome.org/cgi-bin/locus.fpl?locus=AAH1), [ACC1/YNR016C](http://www.yeastgenome.org/cgi-bin/locus.fpl?locus=ACC1), [MVD1/YNR043W](http://www.yeastgenome.org/cgi-bin/locus.fpl?locus=MVD1), [TRM112/YNR046W](http://www.yeastgenome.org/cgi-bin/locus.fpl?locus=TRM112), [LYS9/YNR050C](http://www.yeastgenome.org/cgi-bin/locus.fpl?locus=LYS9), [TRM11/YOL124C](http://www.yeastgenome.org/cgi-bin/locus.fpl?locus=TRM11), [CDC21/YOR074C](http://www.yeastgenome.org/cgi-bin/locus.fpl?locus=CDC21), [RAS1/YOR101W](http://www.yeastgenome.org/cgi-bin/locus.fpl?locus=RAS1), [GLN4/YOR168W](http://www.yeastgenome.org/cgi-bin/locus.fpl?locus=GLN4), [PUT4/YOR348C](http://www.yeastgenome.org/cgi-bin/locus.fpl?locus=PUT4), [GDH1/YOR375C](http://www.yeastgenome.org/cgi-bin/locus.fpl?locus=GDH1), [CAR1/YPL111W](http://www.yeastgenome.org/cgi-bin/locus.fpl?locus=CAR1), [TYW1/YPL207W](http://www.yeastgenome.org/cgi-bin/locus.fpl?locus=TYW1), [GLN1/YPR035W](http://www.yeastgenome.org/cgi-bin/locus.fpl?locus=GLN1), [TKL1/YPR074C](http://www.yeastgenome.org/cgi-bin/locus.fpl?locus=TKL1) |
| [*ribosome assembly*](http://www.yeastgenome.org/cgi-bin/GO/goTerm.pl?goid=42255) | 16 out of 292 genes, 5.5% | 59 out of 7167 genes, 0.8% | 5.68e-07 | [MAK21/YDR060W](http://www.yeastgenome.org/cgi-bin/locus.fpl?locus=MAK21), [SSF2/YDR312W](http://www.yeastgenome.org/cgi-bin/locus.fpl?locus=SSF2), [DBP3/YGL078C](http://www.yeastgenome.org/cgi-bin/locus.fpl?locus=DBP3), [NSR1/YGR159C](http://www.yeastgenome.org/cgi-bin/locus.fpl?locus=NSR1), [NMD3/YHR170W](http://www.yeastgenome.org/cgi-bin/locus.fpl?locus=NMD3), [RIX1/YHR197W](http://www.yeastgenome.org/cgi-bin/locus.fpl?locus=RIX1), [SQT1/YIR012W](http://www.yeastgenome.org/cgi-bin/locus.fpl?locus=SQT1), [MAK11/YKL021C](http://www.yeastgenome.org/cgi-bin/locus.fpl?locus=MAK11), [RPF2/YKR081C](http://www.yeastgenome.org/cgi-bin/locus.fpl?locus=RPF2), [MDN1/YLR106C](http://www.yeastgenome.org/cgi-bin/locus.fpl?locus=MDN1), [DBP9/YLR276C](http://www.yeastgenome.org/cgi-bin/locus.fpl?locus=DBP9), [IPI3/YNL182C](http://www.yeastgenome.org/cgi-bin/locus.fpl?locus=IPI3), [BRX1/YOL077C](http://www.yeastgenome.org/cgi-bin/locus.fpl?locus=BRX1), [NOC2/YOR206W](http://www.yeastgenome.org/cgi-bin/locus.fpl?locus=NOC2), [NIP7/YPL211W](http://www.yeastgenome.org/cgi-bin/locus.fpl?locus=NIP7), [BMS1/YPL217C](http://www.yeastgenome.org/cgi-bin/locus.fpl?locus=BMS1) |
| [*metabolic process*](http://www.yeastgenome.org/cgi-bin/GO/goTerm.pl?goid=8152) | 198 out of 292 genes, 67.8% | 3627 out of 7167 genes, 50.6% | 6.22e-07 | [EFB1/YAL003W](http://www.yeastgenome.org/cgi-bin/locus.fpl?locus=EFB1), [PMT2/YAL023C](http://www.yeastgenome.org/cgi-bin/locus.fpl?locus=PMT2), [FUN12/YAL035W](http://www.yeastgenome.org/cgi-bin/locus.fpl?locus=FUN12), [GDH3/YAL062W](http://www.yeastgenome.org/cgi-bin/locus.fpl?locus=GDH3), [ADE1/YAR015W](http://www.yeastgenome.org/cgi-bin/locus.fpl?locus=ADE1), [UTP20/YBL004W](http://www.yeastgenome.org/cgi-bin/locus.fpl?locus=UTP20), [NCL1/YBL024W](http://www.yeastgenome.org/cgi-bin/locus.fpl?locus=NCL1), [URA7/YBL039C](http://www.yeastgenome.org/cgi-bin/locus.fpl?locus=URA7), [TOD6/YBL054W](http://www.yeastgenome.org/cgi-bin/locus.fpl?locus=TOD6), [CDS1/YBR029C](http://www.yeastgenome.org/cgi-bin/locus.fpl?locus=CDS1), [HMT1/YBR034C](http://www.yeastgenome.org/cgi-bin/locus.fpl?locus=HMT1), [RPG1/YBR079C](http://www.yeastgenome.org/cgi-bin/locus.fpl?locus=RPG1), [POL30/YBR088C](http://www.yeastgenome.org/cgi-bin/locus.fpl?locus=POL30), [PHO3/YBR092C](http://www.yeastgenome.org/cgi-bin/locus.fpl?locus=PHO3), [PHO5/YBR093C](http://www.yeastgenome.org/cgi-bin/locus.fpl?locus=PHO5), [TKL2/YBR117C](http://www.yeastgenome.org/cgi-bin/locus.fpl?locus=TKL2), [MAK5/YBR142W](http://www.yeastgenome.org/cgi-bin/locus.fpl?locus=MAK5), [CNS1/YBR155W](http://www.yeastgenome.org/cgi-bin/locus.fpl?locus=CNS1), [DUR1,2/YBR208C](http://www.yeastgenome.org/cgi-bin/locus.fpl?locus=DUR1), [ENP1/YBR247C](http://www.yeastgenome.org/cgi-bin/locus.fpl?locus=ENP1), [SHM1/YBR263W](http://www.yeastgenome.org/cgi-bin/locus.fpl?locus=SHM1), [YCL003W](http://www.yeastgenome.org/cgi-bin/locus.fpl?locus=YCL003W), [KCC4/YCL024W](http://www.yeastgenome.org/cgi-bin/locus.fpl?locus=KCC4), [SRO9/YCL037C](http://www.yeastgenome.org/cgi-bin/locus.fpl?locus=SRO9), [SPB1/YCL054W](http://www.yeastgenome.org/cgi-bin/locus.fpl?locus=SPB1), [CWH43/YCR017C](http://www.yeastgenome.org/cgi-bin/locus.fpl?locus=CWH43), [FEN1/YCR034W](http://www.yeastgenome.org/cgi-bin/locus.fpl?locus=FEN1), [MATALPHA1/YCR040W](http://www.yeastgenome.org/cgi-bin/locus.fpl?locus=MATALPHA1), [IMG1/YCR046C](http://www.yeastgenome.org/cgi-bin/locus.fpl?locus=IMG1), [BUD31/YCR063W](http://www.yeastgenome.org/cgi-bin/locus.fpl?locus=BUD31), [MCD1/YDL003W](http://www.yeastgenome.org/cgi-bin/locus.fpl?locus=MCD1), [TSR1/YDL060W](http://www.yeastgenome.org/cgi-bin/locus.fpl?locus=TSR1), [NOP14/YDL148C](http://www.yeastgenome.org/cgi-bin/locus.fpl?locus=NOP14), [SAS10/YDL153C](http://www.yeastgenome.org/cgi-bin/locus.fpl?locus=SAS10), [TRM8/YDL201W](http://www.yeastgenome.org/cgi-bin/locus.fpl?locus=TRM8), [GDH2/YDL215C](http://www.yeastgenome.org/cgi-bin/locus.fpl?locus=GDH2), [RRP1/YDR087C](http://www.yeastgenome.org/cgi-bin/locus.fpl?locus=RRP1), [MKC7/YDR144C](http://www.yeastgenome.org/cgi-bin/locus.fpl?locus=MKC7), [SUP35/YDR172W](http://www.yeastgenome.org/cgi-bin/locus.fpl?locus=SUP35), [LYS4/YDR234W](http://www.yeastgenome.org/cgi-bin/locus.fpl?locus=LYS4), [ASP1/YDR321W](http://www.yeastgenome.org/cgi-bin/locus.fpl?locus=ASP1), [UTP4/YDR324C](http://www.yeastgenome.org/cgi-bin/locus.fpl?locus=UTP4), [NCB2/YDR397C](http://www.yeastgenome.org/cgi-bin/locus.fpl?locus=NCB2), [UTP5/YDR398W](http://www.yeastgenome.org/cgi-bin/locus.fpl?locus=UTP5), [HPT1/YDR399W](http://www.yeastgenome.org/cgi-bin/locus.fpl?locus=HPT1), [RRP17/YDR412W](http://www.yeastgenome.org/cgi-bin/locus.fpl?locus=RRP17), [TIF35/YDR429C](http://www.yeastgenome.org/cgi-bin/locus.fpl?locus=TIF35), [DOT1/YDR440W](http://www.yeastgenome.org/cgi-bin/locus.fpl?locus=DOT1), [UTP6/YDR449C](http://www.yeastgenome.org/cgi-bin/locus.fpl?locus=UTP6), [RMT2/YDR465C](http://www.yeastgenome.org/cgi-bin/locus.fpl?locus=RMT2), [PUF6/YDR496C](http://www.yeastgenome.org/cgi-bin/locus.fpl?locus=PUF6), [SNU13/YEL026W](http://www.yeastgenome.org/cgi-bin/locus.fpl?locus=SNU13), [ECM10/YEL030W](http://www.yeastgenome.org/cgi-bin/locus.fpl?locus=ECM10), [HYP2/YEL034W](http://www.yeastgenome.org/cgi-bin/locus.fpl?locus=HYP2), [GDA1/YEL042W](http://www.yeastgenome.org/cgi-bin/locus.fpl?locus=GDA1), [HPA3/YEL066W](http://www.yeastgenome.org/cgi-bin/locus.fpl?locus=HPA3), [PMI40/YER003C](http://www.yeastgenome.org/cgi-bin/locus.fpl?locus=PMI40), [NUG1/YER006W](http://www.yeastgenome.org/cgi-bin/locus.fpl?locus=NUG1), [SAH1/YER043C](http://www.yeastgenome.org/cgi-bin/locus.fpl?locus=SAH1), [YER064C](http://www.yeastgenome.org/cgi-bin/locus.fpl?locus=YER064C), [RNR1/YER070W](http://www.yeastgenome.org/cgi-bin/locus.fpl?locus=RNR1), [UTP7/YER082C](http://www.yeastgenome.org/cgi-bin/locus.fpl?locus=UTP7), [BUD27/YFL023W](http://www.yeastgenome.org/cgi-bin/locus.fpl?locus=BUD27), [OLE1/YGL055W](http://www.yeastgenome.org/cgi-bin/locus.fpl?locus=OLE1), [DBP3/YGL078C](http://www.yeastgenome.org/cgi-bin/locus.fpl?locus=DBP3), [PRP43/YGL120C](http://www.yeastgenome.org/cgi-bin/locus.fpl?locus=PRP43), [ADE5,7/YGL234W](http://www.yeastgenome.org/cgi-bin/locus.fpl?locus=ADE5), [HXK2/YGL253W](http://www.yeastgenome.org/cgi-bin/locus.fpl?locus=HXK2), [YGR054W](http://www.yeastgenome.org/cgi-bin/locus.fpl?locus=YGR054W), [ERG25/YGR060W](http://www.yeastgenome.org/cgi-bin/locus.fpl?locus=ERG25), [GCD2/YGR083C](http://www.yeastgenome.org/cgi-bin/locus.fpl?locus=GCD2), [PDC6/YGR087C](http://www.yeastgenome.org/cgi-bin/locus.fpl?locus=PDC6), [NOP7/YGR103W](http://www.yeastgenome.org/cgi-bin/locus.fpl?locus=NOP7), [PPT1/YGR123C](http://www.yeastgenome.org/cgi-bin/locus.fpl?locus=PPT1), [ENP2/YGR145W](http://www.yeastgenome.org/cgi-bin/locus.fpl?locus=ENP2), [CYS4/YGR155W](http://www.yeastgenome.org/cgi-bin/locus.fpl?locus=CYS4), [CHO2/YGR157W](http://www.yeastgenome.org/cgi-bin/locus.fpl?locus=CHO2), [NSR1/YGR159C](http://www.yeastgenome.org/cgi-bin/locus.fpl?locus=NSR1), [TIF4631/YGR162W](http://www.yeastgenome.org/cgi-bin/locus.fpl?locus=TIF4631), [CRH1/YGR189C](http://www.yeastgenome.org/cgi-bin/locus.fpl?locus=CRH1), [PFK1/YGR240C](http://www.yeastgenome.org/cgi-bin/locus.fpl?locus=PFK1), [GND2/YGR256W](http://www.yeastgenome.org/cgi-bin/locus.fpl?locus=GND2), [PXR1/YGR280C](http://www.yeastgenome.org/cgi-bin/locus.fpl?locus=PXR1), [ZUO1/YGR285C](http://www.yeastgenome.org/cgi-bin/locus.fpl?locus=ZUO1), [PRS3/YHL011C](http://www.yeastgenome.org/cgi-bin/locus.fpl?locus=PRS3), [DUR3/YHL016C](http://www.yeastgenome.org/cgi-bin/locus.fpl?locus=DUR3), [NCP1/YHR042W](http://www.yeastgenome.org/cgi-bin/locus.fpl?locus=NCP1), [CIC1/YHR052W](http://www.yeastgenome.org/cgi-bin/locus.fpl?locus=CIC1), [SSZ1/YHR064C](http://www.yeastgenome.org/cgi-bin/locus.fpl?locus=SSZ1), [DYS1/YHR068W](http://www.yeastgenome.org/cgi-bin/locus.fpl?locus=DYS1), [GAR1/YHR089C](http://www.yeastgenome.org/cgi-bin/locus.fpl?locus=GAR1), [GND1/YHR183W](http://www.yeastgenome.org/cgi-bin/locus.fpl?locus=GND1), [RIX1/YHR197W](http://www.yeastgenome.org/cgi-bin/locus.fpl?locus=RIX1), [IMD2/YHR216W](http://www.yeastgenome.org/cgi-bin/locus.fpl?locus=IMD2), [FAA3/YIL009W](http://www.yeastgenome.org/cgi-bin/locus.fpl?locus=FAA3), [CCT2/YIL142W](http://www.yeastgenome.org/cgi-bin/locus.fpl?locus=CCT2), [DAL2/YIR029W](http://www.yeastgenome.org/cgi-bin/locus.fpl?locus=DAL2), [CCT8/YJL008C](http://www.yeastgenome.org/cgi-bin/locus.fpl?locus=CCT8), [RPC17/YJL011C](http://www.yeastgenome.org/cgi-bin/locus.fpl?locus=RPC17), [MTR4/YJL050W](http://www.yeastgenome.org/cgi-bin/locus.fpl?locus=MTR4), [UTP18/YJL069C](http://www.yeastgenome.org/cgi-bin/locus.fpl?locus=UTP18), [SCP160/YJL080C](http://www.yeastgenome.org/cgi-bin/locus.fpl?locus=SCP160), [URA2/YJL130C](http://www.yeastgenome.org/cgi-bin/locus.fpl?locus=URA2), [CPS1/YJL172W](http://www.yeastgenome.org/cgi-bin/locus.fpl?locus=CPS1), [MNN11/YJL183W](http://www.yeastgenome.org/cgi-bin/locus.fpl?locus=MNN11), [MPP10/YJR002W](http://www.yeastgenome.org/cgi-bin/locus.fpl?locus=MPP10), [ANB1/YJR047C](http://www.yeastgenome.org/cgi-bin/locus.fpl?locus=ANB1), [RPA12/YJR063W](http://www.yeastgenome.org/cgi-bin/locus.fpl?locus=RPA12), [LIA1/YJR070C](http://www.yeastgenome.org/cgi-bin/locus.fpl?locus=LIA1), [URA8/YJR103W](http://www.yeastgenome.org/cgi-bin/locus.fpl?locus=URA8), [YJR120W](http://www.yeastgenome.org/cgi-bin/locus.fpl?locus=YJR120W), [BAT2/YJR148W](http://www.yeastgenome.org/cgi-bin/locus.fpl?locus=BAT2), [AUR1/YKL004W](http://www.yeastgenome.org/cgi-bin/locus.fpl?locus=AUR1), [MAK11/YKL021C](http://www.yeastgenome.org/cgi-bin/locus.fpl?locus=MAK11), [TEF4/YKL081W](http://www.yeastgenome.org/cgi-bin/locus.fpl?locus=TEF4), [EBP2/YKL172W](http://www.yeastgenome.org/cgi-bin/locus.fpl?locus=EBP2), [DPH2/YKL191W](http://www.yeastgenome.org/cgi-bin/locus.fpl?locus=DPH2), [FOX2/YKR009C](http://www.yeastgenome.org/cgi-bin/locus.fpl?locus=FOX2), [GAP1/YKR039W](http://www.yeastgenome.org/cgi-bin/locus.fpl?locus=GAP1), [RPF2/YKR081C](http://www.yeastgenome.org/cgi-bin/locus.fpl?locus=RPF2), [RPL8B/YLL045C](http://www.yeastgenome.org/cgi-bin/locus.fpl?locus=RPL8B), [SPT8/YLR055C](http://www.yeastgenome.org/cgi-bin/locus.fpl?locus=SPT8), [ERG3/YLR056W](http://www.yeastgenome.org/cgi-bin/locus.fpl?locus=ERG3), [FRS1/YLR060W](http://www.yeastgenome.org/cgi-bin/locus.fpl?locus=FRS1), [RPL22A/YLR061W](http://www.yeastgenome.org/cgi-bin/locus.fpl?locus=RPL22A), [MDN1/YLR106C](http://www.yeastgenome.org/cgi-bin/locus.fpl?locus=MDN1), [PDC5/YLR134W](http://www.yeastgenome.org/cgi-bin/locus.fpl?locus=PDC5), [PUT1/YLR142W](http://www.yeastgenome.org/cgi-bin/locus.fpl?locus=PUT1), [STM1/YLR150W](http://www.yeastgenome.org/cgi-bin/locus.fpl?locus=STM1), [DPH5/YLR172C](http://www.yeastgenome.org/cgi-bin/locus.fpl?locus=DPH5), [CBF5/YLR175W](http://www.yeastgenome.org/cgi-bin/locus.fpl?locus=CBF5), [PWP1/YLR196W](http://www.yeastgenome.org/cgi-bin/locus.fpl?locus=PWP1), [NOP56/YLR197W](http://www.yeastgenome.org/cgi-bin/locus.fpl?locus=NOP56), [UTP13/YLR222C](http://www.yeastgenome.org/cgi-bin/locus.fpl?locus=UTP13), [DBP9/YLR276C](http://www.yeastgenome.org/cgi-bin/locus.fpl?locus=DBP9), [GSP1/YLR293C](http://www.yeastgenome.org/cgi-bin/locus.fpl?locus=GSP1), [ADE13/YLR359W](http://www.yeastgenome.org/cgi-bin/locus.fpl?locus=ADE13), [CSR1/YLR380W](http://www.yeastgenome.org/cgi-bin/locus.fpl?locus=CSR1), [DUS3/YLR401C](http://www.yeastgenome.org/cgi-bin/locus.fpl?locus=DUS3), [IMD3/YLR432W](http://www.yeastgenome.org/cgi-bin/locus.fpl?locus=IMD3), [FPR4/YLR449W](http://www.yeastgenome.org/cgi-bin/locus.fpl?locus=FPR4), [LEU3/YLR451W](http://www.yeastgenome.org/cgi-bin/locus.fpl?locus=LEU3), [ERG6/YML008C](http://www.yeastgenome.org/cgi-bin/locus.fpl?locus=ERG6), [TSA1/YML028W](http://www.yeastgenome.org/cgi-bin/locus.fpl?locus=TSA1), [ALO1/YML086C](http://www.yeastgenome.org/cgi-bin/locus.fpl?locus=ALO1), [ERB1/YMR049C](http://www.yeastgenome.org/cgi-bin/locus.fpl?locus=ERB1), [PDS5/YMR076C](http://www.yeastgenome.org/cgi-bin/locus.fpl?locus=PDS5), [UTP15/YMR093W](http://www.yeastgenome.org/cgi-bin/locus.fpl?locus=UTP15), [ADE17/YMR120C](http://www.yeastgenome.org/cgi-bin/locus.fpl?locus=ADE17), [ECM16/YMR128W](http://www.yeastgenome.org/cgi-bin/locus.fpl?locus=ECM16), [NDE1/YMR145C](http://www.yeastgenome.org/cgi-bin/locus.fpl?locus=NDE1), [GUA1/YMR217W](http://www.yeastgenome.org/cgi-bin/locus.fpl?locus=GUA1), [GAS1/YMR307W](http://www.yeastgenome.org/cgi-bin/locus.fpl?locus=GAS1), [NIP1/YMR309C](http://www.yeastgenome.org/cgi-bin/locus.fpl?locus=NIP1), [HEF3/YNL014W](http://www.yeastgenome.org/cgi-bin/locus.fpl?locus=HEF3), [NOP2/YNL061W](http://www.yeastgenome.org/cgi-bin/locus.fpl?locus=NOP2), [POL1/YNL102W](http://www.yeastgenome.org/cgi-bin/locus.fpl?locus=POL1), [DBP2/YNL112W](http://www.yeastgenome.org/cgi-bin/locus.fpl?locus=DBP2), [RPC19/YNL113W](http://www.yeastgenome.org/cgi-bin/locus.fpl?locus=RPC19), [AAH1/YNL141W](http://www.yeastgenome.org/cgi-bin/locus.fpl?locus=AAH1), [IPI3/YNL182C](http://www.yeastgenome.org/cgi-bin/locus.fpl?locus=IPI3), [PUS4/YNL292W](http://www.yeastgenome.org/cgi-bin/locus.fpl?locus=PUS4), [KRI1/YNL308C](http://www.yeastgenome.org/cgi-bin/locus.fpl?locus=KRI1), [ACC1/YNR016C](http://www.yeastgenome.org/cgi-bin/locus.fpl?locus=ACC1), [MVD1/YNR043W](http://www.yeastgenome.org/cgi-bin/locus.fpl?locus=MVD1), [TRM112/YNR046W](http://www.yeastgenome.org/cgi-bin/locus.fpl?locus=TRM112), [LYS9/YNR050C](http://www.yeastgenome.org/cgi-bin/locus.fpl?locus=LYS9), [TRM11/YOL124C](http://www.yeastgenome.org/cgi-bin/locus.fpl?locus=TRM11), [CDC33/YOL139C](http://www.yeastgenome.org/cgi-bin/locus.fpl?locus=CDC33), [RRP6/YOR001W](http://www.yeastgenome.org/cgi-bin/locus.fpl?locus=RRP6), [CDC21/YOR074C](http://www.yeastgenome.org/cgi-bin/locus.fpl?locus=CDC21), [RAS1/YOR101W](http://www.yeastgenome.org/cgi-bin/locus.fpl?locus=RAS1), [RPO31/YOR116C](http://www.yeastgenome.org/cgi-bin/locus.fpl?locus=RPO31), [ARP8/YOR141C](http://www.yeastgenome.org/cgi-bin/locus.fpl?locus=ARP8), [PNO1/YOR145C](http://www.yeastgenome.org/cgi-bin/locus.fpl?locus=PNO1), [GLN4/YOR168W](http://www.yeastgenome.org/cgi-bin/locus.fpl?locus=GLN4), [RRS1/YOR294W](http://www.yeastgenome.org/cgi-bin/locus.fpl?locus=RRS1), [NOP58/YOR310C](http://www.yeastgenome.org/cgi-bin/locus.fpl?locus=NOP58), [RPA43/YOR340C](http://www.yeastgenome.org/cgi-bin/locus.fpl?locus=RPA43), [PUT4/YOR348C](http://www.yeastgenome.org/cgi-bin/locus.fpl?locus=PUT4), [GDH1/YOR375C](http://www.yeastgenome.org/cgi-bin/locus.fpl?locus=GDH1), [RRP12/YPL012W](http://www.yeastgenome.org/cgi-bin/locus.fpl?locus=RRP12), [NOP4/YPL043W](http://www.yeastgenome.org/cgi-bin/locus.fpl?locus=NOP4), [ELP3/YPL086C](http://www.yeastgenome.org/cgi-bin/locus.fpl?locus=ELP3), [NOG1/YPL093W](http://www.yeastgenome.org/cgi-bin/locus.fpl?locus=NOG1), [CAR1/YPL111W](http://www.yeastgenome.org/cgi-bin/locus.fpl?locus=CAR1), [NAN1/YPL126W](http://www.yeastgenome.org/cgi-bin/locus.fpl?locus=NAN1), [NOP53/YPL146C](http://www.yeastgenome.org/cgi-bin/locus.fpl?locus=NOP53), [RPL7B/YPL198W](http://www.yeastgenome.org/cgi-bin/locus.fpl?locus=RPL7B), [TYW1/YPL207W](http://www.yeastgenome.org/cgi-bin/locus.fpl?locus=TYW1), [NIP7/YPL211W](http://www.yeastgenome.org/cgi-bin/locus.fpl?locus=NIP7), [BMS1/YPL217C](http://www.yeastgenome.org/cgi-bin/locus.fpl?locus=BMS1), [RPA135/YPR010C](http://www.yeastgenome.org/cgi-bin/locus.fpl?locus=RPA135), [CSR2/YPR030W](http://www.yeastgenome.org/cgi-bin/locus.fpl?locus=CSR2), [GLN1/YPR035W](http://www.yeastgenome.org/cgi-bin/locus.fpl?locus=GLN1), [TKL1/YPR074C](http://www.yeastgenome.org/cgi-bin/locus.fpl?locus=TKL1), [TIF3/YPR163C](http://www.yeastgenome.org/cgi-bin/locus.fpl?locus=TIF3), [RPC82/YPR190C](http://www.yeastgenome.org/cgi-bin/locus.fpl?locus=RPC82) |
| [*ribosomal large subunit assembly*](http://www.yeastgenome.org/cgi-bin/GO/goTerm.pl?goid=27) | 13 out of 292 genes, 4.5% | 38 out of 7167 genes, 0.5% | 9.33e-07 | [MAK21/YDR060W](http://www.yeastgenome.org/cgi-bin/locus.fpl?locus=MAK21), [SSF2/YDR312W](http://www.yeastgenome.org/cgi-bin/locus.fpl?locus=SSF2), [DBP3/YGL078C](http://www.yeastgenome.org/cgi-bin/locus.fpl?locus=DBP3), [NMD3/YHR170W](http://www.yeastgenome.org/cgi-bin/locus.fpl?locus=NMD3), [RIX1/YHR197W](http://www.yeastgenome.org/cgi-bin/locus.fpl?locus=RIX1), [SQT1/YIR012W](http://www.yeastgenome.org/cgi-bin/locus.fpl?locus=SQT1), [MAK11/YKL021C](http://www.yeastgenome.org/cgi-bin/locus.fpl?locus=MAK11), [RPF2/YKR081C](http://www.yeastgenome.org/cgi-bin/locus.fpl?locus=RPF2), [MDN1/YLR106C](http://www.yeastgenome.org/cgi-bin/locus.fpl?locus=MDN1), [DBP9/YLR276C](http://www.yeastgenome.org/cgi-bin/locus.fpl?locus=DBP9), [IPI3/YNL182C](http://www.yeastgenome.org/cgi-bin/locus.fpl?locus=IPI3), [BRX1/YOL077C](http://www.yeastgenome.org/cgi-bin/locus.fpl?locus=BRX1), [NIP7/YPL211W](http://www.yeastgenome.org/cgi-bin/locus.fpl?locus=NIP7) |
| [*cleavage involved in rRNA processing*](http://www.yeastgenome.org/cgi-bin/GO/goTerm.pl?goid=469) | 16 out of 292 genes, 5.5% | 65 out of 7167 genes, 0.9% | 2.68e-06 | [UTP20/YBL004W](http://www.yeastgenome.org/cgi-bin/locus.fpl?locus=UTP20), [ENP1/YBR247C](http://www.yeastgenome.org/cgi-bin/locus.fpl?locus=ENP1), [NOP14/YDL148C](http://www.yeastgenome.org/cgi-bin/locus.fpl?locus=NOP14), [SAS10/YDL153C](http://www.yeastgenome.org/cgi-bin/locus.fpl?locus=SAS10), [UTP6/YDR449C](http://www.yeastgenome.org/cgi-bin/locus.fpl?locus=UTP6), [UTP7/YER082C](http://www.yeastgenome.org/cgi-bin/locus.fpl?locus=UTP7), [MTR4/YJL050W](http://www.yeastgenome.org/cgi-bin/locus.fpl?locus=MTR4), [UTP18/YJL069C](http://www.yeastgenome.org/cgi-bin/locus.fpl?locus=UTP18), [MPP10/YJR002W](http://www.yeastgenome.org/cgi-bin/locus.fpl?locus=MPP10), [UTP13/YLR222C](http://www.yeastgenome.org/cgi-bin/locus.fpl?locus=UTP13), [GSP1/YLR293C](http://www.yeastgenome.org/cgi-bin/locus.fpl?locus=GSP1), [KRI1/YNL308C](http://www.yeastgenome.org/cgi-bin/locus.fpl?locus=KRI1), [RRP6/YOR001W](http://www.yeastgenome.org/cgi-bin/locus.fpl?locus=RRP6), [PNO1/YOR145C](http://www.yeastgenome.org/cgi-bin/locus.fpl?locus=PNO1), [RRS1/YOR294W](http://www.yeastgenome.org/cgi-bin/locus.fpl?locus=RRS1), [NOP58/YOR310C](http://www.yeastgenome.org/cgi-bin/locus.fpl?locus=NOP58) |
| [*RNA metabolic process*](http://www.yeastgenome.org/cgi-bin/GO/goTerm.pl?goid=16070) | 87 out of 292 genes, 29.8% | 1180 out of 7167 genes, 16.5% | 3.20e-06 | [UTP20/YBL004W](http://www.yeastgenome.org/cgi-bin/locus.fpl?locus=UTP20), [NCL1/YBL024W](http://www.yeastgenome.org/cgi-bin/locus.fpl?locus=NCL1), [TOD6/YBL054W](http://www.yeastgenome.org/cgi-bin/locus.fpl?locus=TOD6), [HMT1/YBR034C](http://www.yeastgenome.org/cgi-bin/locus.fpl?locus=HMT1), [POL30/YBR088C](http://www.yeastgenome.org/cgi-bin/locus.fpl?locus=POL30), [MAK5/YBR142W](http://www.yeastgenome.org/cgi-bin/locus.fpl?locus=MAK5), [ENP1/YBR247C](http://www.yeastgenome.org/cgi-bin/locus.fpl?locus=ENP1), [SPB1/YCL054W](http://www.yeastgenome.org/cgi-bin/locus.fpl?locus=SPB1), [MATALPHA1/YCR040W](http://www.yeastgenome.org/cgi-bin/locus.fpl?locus=MATALPHA1), [BUD31/YCR063W](http://www.yeastgenome.org/cgi-bin/locus.fpl?locus=BUD31), [TSR1/YDL060W](http://www.yeastgenome.org/cgi-bin/locus.fpl?locus=TSR1), [NOP14/YDL148C](http://www.yeastgenome.org/cgi-bin/locus.fpl?locus=NOP14), [SAS10/YDL153C](http://www.yeastgenome.org/cgi-bin/locus.fpl?locus=SAS10), [TRM8/YDL201W](http://www.yeastgenome.org/cgi-bin/locus.fpl?locus=TRM8), [RRP1/YDR087C](http://www.yeastgenome.org/cgi-bin/locus.fpl?locus=RRP1), [SUP35/YDR172W](http://www.yeastgenome.org/cgi-bin/locus.fpl?locus=SUP35), [UTP4/YDR324C](http://www.yeastgenome.org/cgi-bin/locus.fpl?locus=UTP4), [NCB2/YDR397C](http://www.yeastgenome.org/cgi-bin/locus.fpl?locus=NCB2), [UTP5/YDR398W](http://www.yeastgenome.org/cgi-bin/locus.fpl?locus=UTP5), [RRP17/YDR412W](http://www.yeastgenome.org/cgi-bin/locus.fpl?locus=RRP17), [DOT1/YDR440W](http://www.yeastgenome.org/cgi-bin/locus.fpl?locus=DOT1), [UTP6/YDR449C](http://www.yeastgenome.org/cgi-bin/locus.fpl?locus=UTP6), [SNU13/YEL026W](http://www.yeastgenome.org/cgi-bin/locus.fpl?locus=SNU13), [NUG1/YER006W](http://www.yeastgenome.org/cgi-bin/locus.fpl?locus=NUG1), [UTP7/YER082C](http://www.yeastgenome.org/cgi-bin/locus.fpl?locus=UTP7), [DBP3/YGL078C](http://www.yeastgenome.org/cgi-bin/locus.fpl?locus=DBP3), [PRP43/YGL120C](http://www.yeastgenome.org/cgi-bin/locus.fpl?locus=PRP43), [HXK2/YGL253W](http://www.yeastgenome.org/cgi-bin/locus.fpl?locus=HXK2), [NOP7/YGR103W](http://www.yeastgenome.org/cgi-bin/locus.fpl?locus=NOP7), [ENP2/YGR145W](http://www.yeastgenome.org/cgi-bin/locus.fpl?locus=ENP2), [NSR1/YGR159C](http://www.yeastgenome.org/cgi-bin/locus.fpl?locus=NSR1), [PXR1/YGR280C](http://www.yeastgenome.org/cgi-bin/locus.fpl?locus=PXR1), [GAR1/YHR089C](http://www.yeastgenome.org/cgi-bin/locus.fpl?locus=GAR1), [RIX1/YHR197W](http://www.yeastgenome.org/cgi-bin/locus.fpl?locus=RIX1), [RPC17/YJL011C](http://www.yeastgenome.org/cgi-bin/locus.fpl?locus=RPC17), [MTR4/YJL050W](http://www.yeastgenome.org/cgi-bin/locus.fpl?locus=MTR4), [UTP18/YJL069C](http://www.yeastgenome.org/cgi-bin/locus.fpl?locus=UTP18), [SCP160/YJL080C](http://www.yeastgenome.org/cgi-bin/locus.fpl?locus=SCP160), [MPP10/YJR002W](http://www.yeastgenome.org/cgi-bin/locus.fpl?locus=MPP10), [RPA12/YJR063W](http://www.yeastgenome.org/cgi-bin/locus.fpl?locus=RPA12), [MAK11/YKL021C](http://www.yeastgenome.org/cgi-bin/locus.fpl?locus=MAK11), [EBP2/YKL172W](http://www.yeastgenome.org/cgi-bin/locus.fpl?locus=EBP2), [RPF2/YKR081C](http://www.yeastgenome.org/cgi-bin/locus.fpl?locus=RPF2), [SPT8/YLR055C](http://www.yeastgenome.org/cgi-bin/locus.fpl?locus=SPT8), [FRS1/YLR060W](http://www.yeastgenome.org/cgi-bin/locus.fpl?locus=FRS1), [MDN1/YLR106C](http://www.yeastgenome.org/cgi-bin/locus.fpl?locus=MDN1), [CBF5/YLR175W](http://www.yeastgenome.org/cgi-bin/locus.fpl?locus=CBF5), [PWP1/YLR196W](http://www.yeastgenome.org/cgi-bin/locus.fpl?locus=PWP1), [NOP56/YLR197W](http://www.yeastgenome.org/cgi-bin/locus.fpl?locus=NOP56), [UTP13/YLR222C](http://www.yeastgenome.org/cgi-bin/locus.fpl?locus=UTP13), [DBP9/YLR276C](http://www.yeastgenome.org/cgi-bin/locus.fpl?locus=DBP9), [GSP1/YLR293C](http://www.yeastgenome.org/cgi-bin/locus.fpl?locus=GSP1), [DUS3/YLR401C](http://www.yeastgenome.org/cgi-bin/locus.fpl?locus=DUS3), [FPR4/YLR449W](http://www.yeastgenome.org/cgi-bin/locus.fpl?locus=FPR4), [LEU3/YLR451W](http://www.yeastgenome.org/cgi-bin/locus.fpl?locus=LEU3), [ERB1/YMR049C](http://www.yeastgenome.org/cgi-bin/locus.fpl?locus=ERB1), [UTP15/YMR093W](http://www.yeastgenome.org/cgi-bin/locus.fpl?locus=UTP15), [ECM16/YMR128W](http://www.yeastgenome.org/cgi-bin/locus.fpl?locus=ECM16), [GAS1/YMR307W](http://www.yeastgenome.org/cgi-bin/locus.fpl?locus=GAS1), [NOP2/YNL061W](http://www.yeastgenome.org/cgi-bin/locus.fpl?locus=NOP2), [DBP2/YNL112W](http://www.yeastgenome.org/cgi-bin/locus.fpl?locus=DBP2), [RPC19/YNL113W](http://www.yeastgenome.org/cgi-bin/locus.fpl?locus=RPC19), [IPI3/YNL182C](http://www.yeastgenome.org/cgi-bin/locus.fpl?locus=IPI3), [PUS4/YNL292W](http://www.yeastgenome.org/cgi-bin/locus.fpl?locus=PUS4), [KRI1/YNL308C](http://www.yeastgenome.org/cgi-bin/locus.fpl?locus=KRI1), [TRM112/YNR046W](http://www.yeastgenome.org/cgi-bin/locus.fpl?locus=TRM112), [TRM11/YOL124C](http://www.yeastgenome.org/cgi-bin/locus.fpl?locus=TRM11), [CDC33/YOL139C](http://www.yeastgenome.org/cgi-bin/locus.fpl?locus=CDC33), [RRP6/YOR001W](http://www.yeastgenome.org/cgi-bin/locus.fpl?locus=RRP6), [RPO31/YOR116C](http://www.yeastgenome.org/cgi-bin/locus.fpl?locus=RPO31), [PNO1/YOR145C](http://www.yeastgenome.org/cgi-bin/locus.fpl?locus=PNO1), [GLN4/YOR168W](http://www.yeastgenome.org/cgi-bin/locus.fpl?locus=GLN4), [RRS1/YOR294W](http://www.yeastgenome.org/cgi-bin/locus.fpl?locus=RRS1), [NOP58/YOR310C](http://www.yeastgenome.org/cgi-bin/locus.fpl?locus=NOP58), [RPA43/YOR340C](http://www.yeastgenome.org/cgi-bin/locus.fpl?locus=RPA43), [RRP12/YPL012W](http://www.yeastgenome.org/cgi-bin/locus.fpl?locus=RRP12), [NOP4/YPL043W](http://www.yeastgenome.org/cgi-bin/locus.fpl?locus=NOP4), [ELP3/YPL086C](http://www.yeastgenome.org/cgi-bin/locus.fpl?locus=ELP3), [NOG1/YPL093W](http://www.yeastgenome.org/cgi-bin/locus.fpl?locus=NOG1), [NAN1/YPL126W](http://www.yeastgenome.org/cgi-bin/locus.fpl?locus=NAN1), [NOP53/YPL146C](http://www.yeastgenome.org/cgi-bin/locus.fpl?locus=NOP53), [TYW1/YPL207W](http://www.yeastgenome.org/cgi-bin/locus.fpl?locus=TYW1), [NIP7/YPL211W](http://www.yeastgenome.org/cgi-bin/locus.fpl?locus=NIP7), [BMS1/YPL217C](http://www.yeastgenome.org/cgi-bin/locus.fpl?locus=BMS1), [RPA135/YPR010C](http://www.yeastgenome.org/cgi-bin/locus.fpl?locus=RPA135), [CSR2/YPR030W](http://www.yeastgenome.org/cgi-bin/locus.fpl?locus=CSR2), [RPC82/YPR190C](http://www.yeastgenome.org/cgi-bin/locus.fpl?locus=RPC82) |
| [*endonucleolytic cleavage in ITS1 to separate SSU-rRNA from 5.8S rRNA and LSU-rRNA from tricistronic rRNA transcript (SSU-rRNA, 5.8S rRNA, LSU-rRNA)*](http://www.yeastgenome.org/cgi-bin/GO/goTerm.pl?goid=447) | 13 out of 292 genes, 4.5% | 43 out of 7167 genes, 0.6% | 5.24e-06 | [UTP20/YBL004W](http://www.yeastgenome.org/cgi-bin/locus.fpl?locus=UTP20), [ENP1/YBR247C](http://www.yeastgenome.org/cgi-bin/locus.fpl?locus=ENP1), [NOP14/YDL148C](http://www.yeastgenome.org/cgi-bin/locus.fpl?locus=NOP14), [SAS10/YDL153C](http://www.yeastgenome.org/cgi-bin/locus.fpl?locus=SAS10), [UTP6/YDR449C](http://www.yeastgenome.org/cgi-bin/locus.fpl?locus=UTP6), [UTP7/YER082C](http://www.yeastgenome.org/cgi-bin/locus.fpl?locus=UTP7), [UTP18/YJL069C](http://www.yeastgenome.org/cgi-bin/locus.fpl?locus=UTP18), [MPP10/YJR002W](http://www.yeastgenome.org/cgi-bin/locus.fpl?locus=MPP10), [UTP13/YLR222C](http://www.yeastgenome.org/cgi-bin/locus.fpl?locus=UTP13), [KRI1/YNL308C](http://www.yeastgenome.org/cgi-bin/locus.fpl?locus=KRI1), [PNO1/YOR145C](http://www.yeastgenome.org/cgi-bin/locus.fpl?locus=PNO1), [RRS1/YOR294W](http://www.yeastgenome.org/cgi-bin/locus.fpl?locus=RRS1), [NOP58/YOR310C](http://www.yeastgenome.org/cgi-bin/locus.fpl?locus=NOP58) |
| [*endonucleolytic cleavage involved in rRNA processing*](http://www.yeastgenome.org/cgi-bin/GO/goTerm.pl?goid=478) | 13 out of 292 genes, 4.5% | 45 out of 7167 genes, 0.6% | 9.72e-06 | [UTP20/YBL004W](http://www.yeastgenome.org/cgi-bin/locus.fpl?locus=UTP20), [ENP1/YBR247C](http://www.yeastgenome.org/cgi-bin/locus.fpl?locus=ENP1), [NOP14/YDL148C](http://www.yeastgenome.org/cgi-bin/locus.fpl?locus=NOP14), [SAS10/YDL153C](http://www.yeastgenome.org/cgi-bin/locus.fpl?locus=SAS10), [UTP6/YDR449C](http://www.yeastgenome.org/cgi-bin/locus.fpl?locus=UTP6), [UTP7/YER082C](http://www.yeastgenome.org/cgi-bin/locus.fpl?locus=UTP7), [UTP18/YJL069C](http://www.yeastgenome.org/cgi-bin/locus.fpl?locus=UTP18), [MPP10/YJR002W](http://www.yeastgenome.org/cgi-bin/locus.fpl?locus=MPP10), [UTP13/YLR222C](http://www.yeastgenome.org/cgi-bin/locus.fpl?locus=UTP13), [KRI1/YNL308C](http://www.yeastgenome.org/cgi-bin/locus.fpl?locus=KRI1), [PNO1/YOR145C](http://www.yeastgenome.org/cgi-bin/locus.fpl?locus=PNO1), [RRS1/YOR294W](http://www.yeastgenome.org/cgi-bin/locus.fpl?locus=RRS1), [NOP58/YOR310C](http://www.yeastgenome.org/cgi-bin/locus.fpl?locus=NOP58) |
| [*endonucleolytic cleavage of tricistronic rRNA transcript (SSU-rRNA, 5.8S rRNA, LSU-rRNA)*](http://www.yeastgenome.org/cgi-bin/GO/goTerm.pl?goid=479) | 13 out of 292 genes, 4.5% | 45 out of 7167 genes, 0.6% | 9.72e-06 | [UTP20/YBL004W](http://www.yeastgenome.org/cgi-bin/locus.fpl?locus=UTP20), [ENP1/YBR247C](http://www.yeastgenome.org/cgi-bin/locus.fpl?locus=ENP1), [NOP14/YDL148C](http://www.yeastgenome.org/cgi-bin/locus.fpl?locus=NOP14), [SAS10/YDL153C](http://www.yeastgenome.org/cgi-bin/locus.fpl?locus=SAS10), [UTP6/YDR449C](http://www.yeastgenome.org/cgi-bin/locus.fpl?locus=UTP6), [UTP7/YER082C](http://www.yeastgenome.org/cgi-bin/locus.fpl?locus=UTP7), [UTP18/YJL069C](http://www.yeastgenome.org/cgi-bin/locus.fpl?locus=UTP18), [MPP10/YJR002W](http://www.yeastgenome.org/cgi-bin/locus.fpl?locus=MPP10), [UTP13/YLR222C](http://www.yeastgenome.org/cgi-bin/locus.fpl?locus=UTP13), [KRI1/YNL308C](http://www.yeastgenome.org/cgi-bin/locus.fpl?locus=KRI1), [PNO1/YOR145C](http://www.yeastgenome.org/cgi-bin/locus.fpl?locus=PNO1), [RRS1/YOR294W](http://www.yeastgenome.org/cgi-bin/locus.fpl?locus=RRS1), [NOP58/YOR310C](http://www.yeastgenome.org/cgi-bin/locus.fpl?locus=NOP58) |
| [*ribosomal subunit assembly*](http://www.yeastgenome.org/cgi-bin/GO/goTerm.pl?goid=42257) | 14 out of 292 genes, 4.8% | 54 out of 7167 genes, 0.8% | 1.25e-05 | [MAK21/YDR060W](http://www.yeastgenome.org/cgi-bin/locus.fpl?locus=MAK21), [SSF2/YDR312W](http://www.yeastgenome.org/cgi-bin/locus.fpl?locus=SSF2), [DBP3/YGL078C](http://www.yeastgenome.org/cgi-bin/locus.fpl?locus=DBP3), [NSR1/YGR159C](http://www.yeastgenome.org/cgi-bin/locus.fpl?locus=NSR1), [NMD3/YHR170W](http://www.yeastgenome.org/cgi-bin/locus.fpl?locus=NMD3), [RIX1/YHR197W](http://www.yeastgenome.org/cgi-bin/locus.fpl?locus=RIX1), [SQT1/YIR012W](http://www.yeastgenome.org/cgi-bin/locus.fpl?locus=SQT1), [MAK11/YKL021C](http://www.yeastgenome.org/cgi-bin/locus.fpl?locus=MAK11), [RPF2/YKR081C](http://www.yeastgenome.org/cgi-bin/locus.fpl?locus=RPF2), [MDN1/YLR106C](http://www.yeastgenome.org/cgi-bin/locus.fpl?locus=MDN1), [DBP9/YLR276C](http://www.yeastgenome.org/cgi-bin/locus.fpl?locus=DBP9), [IPI3/YNL182C](http://www.yeastgenome.org/cgi-bin/locus.fpl?locus=IPI3), [BRX1/YOL077C](http://www.yeastgenome.org/cgi-bin/locus.fpl?locus=BRX1), [NIP7/YPL211W](http://www.yeastgenome.org/cgi-bin/locus.fpl?locus=NIP7) |
