# Supplementary material for: A Plant Virus Movement Protein Regulates the Gcn2p Kinase in Budding Yeast
Source: PLoS One. 2011 Nov 8;6(11):e27409. doi: 10.1371/journal.pone.0027409 (PMC3210792; doi:10.1371/journal.pone.0027409)
Supplement: Table S3 — Functional classification of repressed genes in MPpnrsv versus MPpnrsvΔHR expressing yeast strains. (DOC) [file pone.0027409.s004.doc]

**TABLE S3.** Functional classification of repressed genes in MPpnrsv versus MPpnrsvΔHR expressing yeast strains.

| ***Gene Ontology term*** | ***Cluster frequency*** | ***Genome frequency of use*** | ***Corrected***  ***P-value*** | ***Genes annotated to the term*** |
| --- | --- | --- | --- | --- |
| [*organic acid biosynthetic process*](http://www.yeastgenome.org/cgi-bin/GO/goTerm.pl?goid=16053) | 32 out of 302 genes, 10.6% | 146 out of 7167 genes, 2.0% | 3.10e-12 | [HIS7/YBR248C](http://www.yeastgenome.org/cgi-bin/locus.fpl?locus=HIS7), [TRP4/YDR354W](http://www.yeastgenome.org/cgi-bin/locus.fpl?locus=TRP4), [HOM3/YER052C](http://www.yeastgenome.org/cgi-bin/locus.fpl?locus=HOM3), [ARG5,6/YER069W](http://www.yeastgenome.org/cgi-bin/locus.fpl?locus=ARG5), [ALD5/YER073W](http://www.yeastgenome.org/cgi-bin/locus.fpl?locus=ALD5), [MET6/YER091C](http://www.yeastgenome.org/cgi-bin/locus.fpl?locus=MET6), [LEU1/YGL009C](http://www.yeastgenome.org/cgi-bin/locus.fpl?locus=LEU1), [MET13/YGL125W](http://www.yeastgenome.org/cgi-bin/locus.fpl?locus=MET13), [STR3/YGL184C](http://www.yeastgenome.org/cgi-bin/locus.fpl?locus=STR3), [ARG4/YHR018C](http://www.yeastgenome.org/cgi-bin/locus.fpl?locus=ARG4), [BAT1/YHR208W](http://www.yeastgenome.org/cgi-bin/locus.fpl?locus=BAT1), [VHR1/YIL056W](http://www.yeastgenome.org/cgi-bin/locus.fpl?locus=VHR1), [HIS5/YIL116W](http://www.yeastgenome.org/cgi-bin/locus.fpl?locus=HIS5), [MET28/YIR017C](http://www.yeastgenome.org/cgi-bin/locus.fpl?locus=MET28), [BNA3/YJL060W](http://www.yeastgenome.org/cgi-bin/locus.fpl?locus=BNA3), [ARG2/YJL071W](http://www.yeastgenome.org/cgi-bin/locus.fpl?locus=ARG2), [ARG3/YJL088W](http://www.yeastgenome.org/cgi-bin/locus.fpl?locus=ARG3), [ILV3/YJR016C](http://www.yeastgenome.org/cgi-bin/locus.fpl?locus=ILV3), [MET1/YKR069W](http://www.yeastgenome.org/cgi-bin/locus.fpl?locus=MET1), [ILV5/YLR355C](http://www.yeastgenome.org/cgi-bin/locus.fpl?locus=ILV5), [ARG7/YMR062C](http://www.yeastgenome.org/cgi-bin/locus.fpl?locus=ARG7), [ILV2/YMR108W](http://www.yeastgenome.org/cgi-bin/locus.fpl?locus=ILV2), [LEU4/YNL104C](http://www.yeastgenome.org/cgi-bin/locus.fpl?locus=LEU4), [MET2/YNL277W](http://www.yeastgenome.org/cgi-bin/locus.fpl?locus=MET2), [BIO5/YNR056C](http://www.yeastgenome.org/cgi-bin/locus.fpl?locus=BIO5), [BIO4/YNR057C](http://www.yeastgenome.org/cgi-bin/locus.fpl?locus=BIO4), [BIO3/YNR058W](http://www.yeastgenome.org/cgi-bin/locus.fpl?locus=BIO3), [ARG8/YOL140W](http://www.yeastgenome.org/cgi-bin/locus.fpl?locus=ARG8), [LEU9/YOR108W](http://www.yeastgenome.org/cgi-bin/locus.fpl?locus=LEU9), [ORT1/YOR130C](http://www.yeastgenome.org/cgi-bin/locus.fpl?locus=ORT1), [HIS3/YOR202W](http://www.yeastgenome.org/cgi-bin/locus.fpl?locus=HIS3), [CPA1/YOR303W](http://www.yeastgenome.org/cgi-bin/locus.fpl?locus=CPA1) |
| [*carboxylic acid biosynthetic process*](http://www.yeastgenome.org/cgi-bin/GO/goTerm.pl?goid=46394) | 32 out of 302 genes, 10.6% | 146 out of 7167 genes, 2.0% | 3.10e-12 | [HIS7/YBR248C](http://www.yeastgenome.org/cgi-bin/locus.fpl?locus=HIS7), [TRP4/YDR354W](http://www.yeastgenome.org/cgi-bin/locus.fpl?locus=TRP4), [HOM3/YER052C](http://www.yeastgenome.org/cgi-bin/locus.fpl?locus=HOM3), [ARG5,6/YER069W](http://www.yeastgenome.org/cgi-bin/locus.fpl?locus=ARG5), [ALD5/YER073W](http://www.yeastgenome.org/cgi-bin/locus.fpl?locus=ALD5), [MET6/YER091C](http://www.yeastgenome.org/cgi-bin/locus.fpl?locus=MET6), [LEU1/YGL009C](http://www.yeastgenome.org/cgi-bin/locus.fpl?locus=LEU1), [MET13/YGL125W](http://www.yeastgenome.org/cgi-bin/locus.fpl?locus=MET13), [STR3/YGL184C](http://www.yeastgenome.org/cgi-bin/locus.fpl?locus=STR3), [ARG4/YHR018C](http://www.yeastgenome.org/cgi-bin/locus.fpl?locus=ARG4), [BAT1/YHR208W](http://www.yeastgenome.org/cgi-bin/locus.fpl?locus=BAT1), [VHR1/YIL056W](http://www.yeastgenome.org/cgi-bin/locus.fpl?locus=VHR1), [HIS5/YIL116W](http://www.yeastgenome.org/cgi-bin/locus.fpl?locus=HIS5), [MET28/YIR017C](http://www.yeastgenome.org/cgi-bin/locus.fpl?locus=MET28), [BNA3/YJL060W](http://www.yeastgenome.org/cgi-bin/locus.fpl?locus=BNA3), [ARG2/YJL071W](http://www.yeastgenome.org/cgi-bin/locus.fpl?locus=ARG2), [ARG3/YJL088W](http://www.yeastgenome.org/cgi-bin/locus.fpl?locus=ARG3), [ILV3/YJR016C](http://www.yeastgenome.org/cgi-bin/locus.fpl?locus=ILV3), [MET1/YKR069W](http://www.yeastgenome.org/cgi-bin/locus.fpl?locus=MET1), [ILV5/YLR355C](http://www.yeastgenome.org/cgi-bin/locus.fpl?locus=ILV5), [ARG7/YMR062C](http://www.yeastgenome.org/cgi-bin/locus.fpl?locus=ARG7), [ILV2/YMR108W](http://www.yeastgenome.org/cgi-bin/locus.fpl?locus=ILV2), [LEU4/YNL104C](http://www.yeastgenome.org/cgi-bin/locus.fpl?locus=LEU4), [MET2/YNL277W](http://www.yeastgenome.org/cgi-bin/locus.fpl?locus=MET2), [BIO5/YNR056C](http://www.yeastgenome.org/cgi-bin/locus.fpl?locus=BIO5), [BIO4/YNR057C](http://www.yeastgenome.org/cgi-bin/locus.fpl?locus=BIO4), [BIO3/YNR058W](http://www.yeastgenome.org/cgi-bin/locus.fpl?locus=BIO3), [ARG8/YOL140W](http://www.yeastgenome.org/cgi-bin/locus.fpl?locus=ARG8), [LEU9/YOR108W](http://www.yeastgenome.org/cgi-bin/locus.fpl?locus=LEU9), [ORT1/YOR130C](http://www.yeastgenome.org/cgi-bin/locus.fpl?locus=ORT1), [HIS3/YOR202W](http://www.yeastgenome.org/cgi-bin/locus.fpl?locus=HIS3), [CPA1/YOR303W](http://www.yeastgenome.org/cgi-bin/locus.fpl?locus=CPA1) |
| [*small molecule metabolic process*](http://www.yeastgenome.org/cgi-bin/GO/goTerm.pl?goid=44281) | 81 out of 302 genes, 26.8% | 839 out of 7167 genes, 11.7% | 8.85e-11 | [UGA2/YBR006W](http://www.yeastgenome.org/cgi-bin/locus.fpl?locus=UGA2), [PDX3/YBR035C](http://www.yeastgenome.org/cgi-bin/locus.fpl?locus=PDX3), [VID24/YBR105C](http://www.yeastgenome.org/cgi-bin/locus.fpl?locus=VID24), [YSA1/YBR111C](http://www.yeastgenome.org/cgi-bin/locus.fpl?locus=YSA1), [HIS7/YBR248C](http://www.yeastgenome.org/cgi-bin/locus.fpl?locus=HIS7), [RIB5/YBR256C](http://www.yeastgenome.org/cgi-bin/locus.fpl?locus=RIB5), [GLK1/YCL040W](http://www.yeastgenome.org/cgi-bin/locus.fpl?locus=GLK1), [HSP30/YCR021C](http://www.yeastgenome.org/cgi-bin/locus.fpl?locus=HSP30), [UGA3/YDL170W](http://www.yeastgenome.org/cgi-bin/locus.fpl?locus=UGA3), [NRG1/YDR043C](http://www.yeastgenome.org/cgi-bin/locus.fpl?locus=NRG1), [TRP4/YDR354W](http://www.yeastgenome.org/cgi-bin/locus.fpl?locus=TRP4), [RIB3/YDR487C](http://www.yeastgenome.org/cgi-bin/locus.fpl?locus=RIB3), [BUD16/YEL029C](http://www.yeastgenome.org/cgi-bin/locus.fpl?locus=BUD16), [YAT2/YER024W](http://www.yeastgenome.org/cgi-bin/locus.fpl?locus=YAT2), [HOM3/YER052C](http://www.yeastgenome.org/cgi-bin/locus.fpl?locus=HOM3), [ARG5,6/YER069W](http://www.yeastgenome.org/cgi-bin/locus.fpl?locus=ARG5), [ALD5/YER073W](http://www.yeastgenome.org/cgi-bin/locus.fpl?locus=ALD5), [MET6/YER091C](http://www.yeastgenome.org/cgi-bin/locus.fpl?locus=MET6), [GSY1/YFR015C](http://www.yeastgenome.org/cgi-bin/locus.fpl?locus=GSY1), [LEU1/YGL009C](http://www.yeastgenome.org/cgi-bin/locus.fpl?locus=LEU1), [PNC1/YGL037C](http://www.yeastgenome.org/cgi-bin/locus.fpl?locus=PNC1), [MET13/YGL125W](http://www.yeastgenome.org/cgi-bin/locus.fpl?locus=MET13), [STR3/YGL184C](http://www.yeastgenome.org/cgi-bin/locus.fpl?locus=STR3), [STF2/YGR008C](http://www.yeastgenome.org/cgi-bin/locus.fpl?locus=STF2), [SOL4/YGR248W](http://www.yeastgenome.org/cgi-bin/locus.fpl?locus=SOL4), [FOL2/YGR267C](http://www.yeastgenome.org/cgi-bin/locus.fpl?locus=FOL2), [ARG4/YHR018C](http://www.yeastgenome.org/cgi-bin/locus.fpl?locus=ARG4), [PAN5/YHR063C](http://www.yeastgenome.org/cgi-bin/locus.fpl?locus=PAN5), [BAT1/YHR208W](http://www.yeastgenome.org/cgi-bin/locus.fpl?locus=BAT1), [VHR1/YIL056W](http://www.yeastgenome.org/cgi-bin/locus.fpl?locus=VHR1), [HIS5/YIL116W](http://www.yeastgenome.org/cgi-bin/locus.fpl?locus=HIS5), [MET28/YIR017C](http://www.yeastgenome.org/cgi-bin/locus.fpl?locus=MET28), [BNA3/YJL060W](http://www.yeastgenome.org/cgi-bin/locus.fpl?locus=BNA3), [ARG2/YJL071W](http://www.yeastgenome.org/cgi-bin/locus.fpl?locus=ARG2), [ARG3/YJL088W](http://www.yeastgenome.org/cgi-bin/locus.fpl?locus=ARG3), [SIP4/YJL089W](http://www.yeastgenome.org/cgi-bin/locus.fpl?locus=SIP4), [MET3/YJR010W](http://www.yeastgenome.org/cgi-bin/locus.fpl?locus=MET3), [ILV3/YJR016C](http://www.yeastgenome.org/cgi-bin/locus.fpl?locus=ILV3), [BNA1/YJR025C](http://www.yeastgenome.org/cgi-bin/locus.fpl?locus=BNA1), [STR2/YJR130C](http://www.yeastgenome.org/cgi-bin/locus.fpl?locus=STR2), [MET14/YKL001C](http://www.yeastgenome.org/cgi-bin/locus.fpl?locus=MET14), [UGP1/YKL035W](http://www.yeastgenome.org/cgi-bin/locus.fpl?locus=UGP1), [YJU3/YKL094W](http://www.yeastgenome.org/cgi-bin/locus.fpl?locus=YJU3), [MCR1/YKL150W](http://www.yeastgenome.org/cgi-bin/locus.fpl?locus=MCR1), [SRY1/YKL218C](http://www.yeastgenome.org/cgi-bin/locus.fpl?locus=SRY1), [GLG1/YKR058W](http://www.yeastgenome.org/cgi-bin/locus.fpl?locus=GLG1), [MET1/YKR069W](http://www.yeastgenome.org/cgi-bin/locus.fpl?locus=MET1), [ARV1/YLR242C](http://www.yeastgenome.org/cgi-bin/locus.fpl?locus=ARV1), [SYM1/YLR251W](http://www.yeastgenome.org/cgi-bin/locus.fpl?locus=SYM1), [GSY2/YLR258W](http://www.yeastgenome.org/cgi-bin/locus.fpl?locus=GSY2), [ILV5/YLR355C](http://www.yeastgenome.org/cgi-bin/locus.fpl?locus=ILV5), [FMS1/YMR020W](http://www.yeastgenome.org/cgi-bin/locus.fpl?locus=FMS1), [ARG7/YMR062C](http://www.yeastgenome.org/cgi-bin/locus.fpl?locus=ARG7), [SNZ1/YMR096W](http://www.yeastgenome.org/cgi-bin/locus.fpl?locus=SNZ1), [PGM2/YMR105C](http://www.yeastgenome.org/cgi-bin/locus.fpl?locus=PGM2), [ILV2/YMR108W](http://www.yeastgenome.org/cgi-bin/locus.fpl?locus=ILV2), [GAD1/YMR250W](http://www.yeastgenome.org/cgi-bin/locus.fpl?locus=GAD1), [URA10/YMR271C](http://www.yeastgenome.org/cgi-bin/locus.fpl?locus=URA10), [LEU4/YNL104C](http://www.yeastgenome.org/cgi-bin/locus.fpl?locus=LEU4), [NRK1/YNL129W](http://www.yeastgenome.org/cgi-bin/locus.fpl?locus=NRK1), [ZWF1/YNL241C](http://www.yeastgenome.org/cgi-bin/locus.fpl?locus=ZWF1), [MET2/YNL277W](http://www.yeastgenome.org/cgi-bin/locus.fpl?locus=MET2), [BIO5/YNR056C](http://www.yeastgenome.org/cgi-bin/locus.fpl?locus=BIO5), [BIO4/YNR057C](http://www.yeastgenome.org/cgi-bin/locus.fpl?locus=BIO4), [BIO3/YNR058W](http://www.yeastgenome.org/cgi-bin/locus.fpl?locus=BIO3), [OPI10/YOL032W](http://www.yeastgenome.org/cgi-bin/locus.fpl?locus=OPI10), [ARG8/YOL140W](http://www.yeastgenome.org/cgi-bin/locus.fpl?locus=ARG8), [LEU9/YOR108W](http://www.yeastgenome.org/cgi-bin/locus.fpl?locus=LEU9), [GCY1/YOR120W](http://www.yeastgenome.org/cgi-bin/locus.fpl?locus=GCY1), [ORT1/YOR130C](http://www.yeastgenome.org/cgi-bin/locus.fpl?locus=ORT1), [HIS3/YOR202W](http://www.yeastgenome.org/cgi-bin/locus.fpl?locus=HIS3), [CPA1/YOR303W](http://www.yeastgenome.org/cgi-bin/locus.fpl?locus=CPA1), [PIP2/YOR363C](http://www.yeastgenome.org/cgi-bin/locus.fpl?locus=PIP2), [SRL4/YPL033C](http://www.yeastgenome.org/cgi-bin/locus.fpl?locus=SRL4), [PDR12/YPL058C](http://www.yeastgenome.org/cgi-bin/locus.fpl?locus=PDR12), [EEB1/YPL095C](http://www.yeastgenome.org/cgi-bin/locus.fpl?locus=EEB1), [POS5/YPL188W](http://www.yeastgenome.org/cgi-bin/locus.fpl?locus=POS5), [YAH1/YPL252C](http://www.yeastgenome.org/cgi-bin/locus.fpl?locus=YAH1), [CIT3/YPR001W](http://www.yeastgenome.org/cgi-bin/locus.fpl?locus=CIT3), [MET16/YPR167C](http://www.yeastgenome.org/cgi-bin/locus.fpl?locus=MET16), [GDB1/YPR184W](http://www.yeastgenome.org/cgi-bin/locus.fpl?locus=GDB1) |
| [*carboxylic acid metabolic process*](http://www.yeastgenome.org/cgi-bin/GO/goTerm.pl?goid=19752) | 47 out of 302 genes, 15.6% | 344 out of 7167 genes, 4.8% | 1.99e-10 | [UGA2/YBR006W](http://www.yeastgenome.org/cgi-bin/locus.fpl?locus=UGA2), [PDX3/YBR035C](http://www.yeastgenome.org/cgi-bin/locus.fpl?locus=PDX3), [VID24/YBR105C](http://www.yeastgenome.org/cgi-bin/locus.fpl?locus=VID24), [HIS7/YBR248C](http://www.yeastgenome.org/cgi-bin/locus.fpl?locus=HIS7), [TRP4/YDR354W](http://www.yeastgenome.org/cgi-bin/locus.fpl?locus=TRP4), [YAT2/YER024W](http://www.yeastgenome.org/cgi-bin/locus.fpl?locus=YAT2), [HOM3/YER052C](http://www.yeastgenome.org/cgi-bin/locus.fpl?locus=HOM3), [ARG5,6/YER069W](http://www.yeastgenome.org/cgi-bin/locus.fpl?locus=ARG5), [ALD5/YER073W](http://www.yeastgenome.org/cgi-bin/locus.fpl?locus=ALD5), [MET6/YER091C](http://www.yeastgenome.org/cgi-bin/locus.fpl?locus=MET6), [LEU1/YGL009C](http://www.yeastgenome.org/cgi-bin/locus.fpl?locus=LEU1), [MET13/YGL125W](http://www.yeastgenome.org/cgi-bin/locus.fpl?locus=MET13), [STR3/YGL184C](http://www.yeastgenome.org/cgi-bin/locus.fpl?locus=STR3), [FOL2/YGR267C](http://www.yeastgenome.org/cgi-bin/locus.fpl?locus=FOL2), [ARG4/YHR018C](http://www.yeastgenome.org/cgi-bin/locus.fpl?locus=ARG4), [BAT1/YHR208W](http://www.yeastgenome.org/cgi-bin/locus.fpl?locus=BAT1), [VHR1/YIL056W](http://www.yeastgenome.org/cgi-bin/locus.fpl?locus=VHR1), [HIS5/YIL116W](http://www.yeastgenome.org/cgi-bin/locus.fpl?locus=HIS5), [MET28/YIR017C](http://www.yeastgenome.org/cgi-bin/locus.fpl?locus=MET28), [BNA3/YJL060W](http://www.yeastgenome.org/cgi-bin/locus.fpl?locus=BNA3), [ARG2/YJL071W](http://www.yeastgenome.org/cgi-bin/locus.fpl?locus=ARG2), [ARG3/YJL088W](http://www.yeastgenome.org/cgi-bin/locus.fpl?locus=ARG3), [SIP4/YJL089W](http://www.yeastgenome.org/cgi-bin/locus.fpl?locus=SIP4), [MET3/YJR010W](http://www.yeastgenome.org/cgi-bin/locus.fpl?locus=MET3), [ILV3/YJR016C](http://www.yeastgenome.org/cgi-bin/locus.fpl?locus=ILV3), [STR2/YJR130C](http://www.yeastgenome.org/cgi-bin/locus.fpl?locus=STR2), [MET14/YKL001C](http://www.yeastgenome.org/cgi-bin/locus.fpl?locus=MET14), [MET1/YKR069W](http://www.yeastgenome.org/cgi-bin/locus.fpl?locus=MET1), [ILV5/YLR355C](http://www.yeastgenome.org/cgi-bin/locus.fpl?locus=ILV5), [ARG7/YMR062C](http://www.yeastgenome.org/cgi-bin/locus.fpl?locus=ARG7), [ILV2/YMR108W](http://www.yeastgenome.org/cgi-bin/locus.fpl?locus=ILV2), [GAD1/YMR250W](http://www.yeastgenome.org/cgi-bin/locus.fpl?locus=GAD1), [LEU4/YNL104C](http://www.yeastgenome.org/cgi-bin/locus.fpl?locus=LEU4), [MET2/YNL277W](http://www.yeastgenome.org/cgi-bin/locus.fpl?locus=MET2), [BIO5/YNR056C](http://www.yeastgenome.org/cgi-bin/locus.fpl?locus=BIO5), [BIO4/YNR057C](http://www.yeastgenome.org/cgi-bin/locus.fpl?locus=BIO4), [BIO3/YNR058W](http://www.yeastgenome.org/cgi-bin/locus.fpl?locus=BIO3), [ARG8/YOL140W](http://www.yeastgenome.org/cgi-bin/locus.fpl?locus=ARG8), [LEU9/YOR108W](http://www.yeastgenome.org/cgi-bin/locus.fpl?locus=LEU9), [ORT1/YOR130C](http://www.yeastgenome.org/cgi-bin/locus.fpl?locus=ORT1), [HIS3/YOR202W](http://www.yeastgenome.org/cgi-bin/locus.fpl?locus=HIS3), [CPA1/YOR303W](http://www.yeastgenome.org/cgi-bin/locus.fpl?locus=CPA1), [PIP2/YOR363C](http://www.yeastgenome.org/cgi-bin/locus.fpl?locus=PIP2), [PDR12/YPL058C](http://www.yeastgenome.org/cgi-bin/locus.fpl?locus=PDR12), [EEB1/YPL095C](http://www.yeastgenome.org/cgi-bin/locus.fpl?locus=EEB1), [CIT3/YPR001W](http://www.yeastgenome.org/cgi-bin/locus.fpl?locus=CIT3), [MET16/YPR167C](http://www.yeastgenome.org/cgi-bin/locus.fpl?locus=MET16) |
| [*oxoacid metabolic process*](http://www.yeastgenome.org/cgi-bin/GO/goTerm.pl?goid=43436) | 47 out of 302 genes, 15.6% | 344 out of 7167 genes, 4.8% | 1.99e-10 | [UGA2/YBR006W](http://www.yeastgenome.org/cgi-bin/locus.fpl?locus=UGA2), [PDX3/YBR035C](http://www.yeastgenome.org/cgi-bin/locus.fpl?locus=PDX3), [VID24/YBR105C](http://www.yeastgenome.org/cgi-bin/locus.fpl?locus=VID24), [HIS7/YBR248C](http://www.yeastgenome.org/cgi-bin/locus.fpl?locus=HIS7), [TRP4/YDR354W](http://www.yeastgenome.org/cgi-bin/locus.fpl?locus=TRP4), [YAT2/YER024W](http://www.yeastgenome.org/cgi-bin/locus.fpl?locus=YAT2), [HOM3/YER052C](http://www.yeastgenome.org/cgi-bin/locus.fpl?locus=HOM3), [ARG5,6/YER069W](http://www.yeastgenome.org/cgi-bin/locus.fpl?locus=ARG5), [ALD5/YER073W](http://www.yeastgenome.org/cgi-bin/locus.fpl?locus=ALD5), [MET6/YER091C](http://www.yeastgenome.org/cgi-bin/locus.fpl?locus=MET6), [LEU1/YGL009C](http://www.yeastgenome.org/cgi-bin/locus.fpl?locus=LEU1), [MET13/YGL125W](http://www.yeastgenome.org/cgi-bin/locus.fpl?locus=MET13), [STR3/YGL184C](http://www.yeastgenome.org/cgi-bin/locus.fpl?locus=STR3), [FOL2/YGR267C](http://www.yeastgenome.org/cgi-bin/locus.fpl?locus=FOL2), [ARG4/YHR018C](http://www.yeastgenome.org/cgi-bin/locus.fpl?locus=ARG4), [BAT1/YHR208W](http://www.yeastgenome.org/cgi-bin/locus.fpl?locus=BAT1), [VHR1/YIL056W](http://www.yeastgenome.org/cgi-bin/locus.fpl?locus=VHR1), [HIS5/YIL116W](http://www.yeastgenome.org/cgi-bin/locus.fpl?locus=HIS5), [MET28/YIR017C](http://www.yeastgenome.org/cgi-bin/locus.fpl?locus=MET28), [BNA3/YJL060W](http://www.yeastgenome.org/cgi-bin/locus.fpl?locus=BNA3), [ARG2/YJL071W](http://www.yeastgenome.org/cgi-bin/locus.fpl?locus=ARG2), [ARG3/YJL088W](http://www.yeastgenome.org/cgi-bin/locus.fpl?locus=ARG3), [SIP4/YJL089W](http://www.yeastgenome.org/cgi-bin/locus.fpl?locus=SIP4), [MET3/YJR010W](http://www.yeastgenome.org/cgi-bin/locus.fpl?locus=MET3), [ILV3/YJR016C](http://www.yeastgenome.org/cgi-bin/locus.fpl?locus=ILV3), [STR2/YJR130C](http://www.yeastgenome.org/cgi-bin/locus.fpl?locus=STR2), [MET14/YKL001C](http://www.yeastgenome.org/cgi-bin/locus.fpl?locus=MET14), [MET1/YKR069W](http://www.yeastgenome.org/cgi-bin/locus.fpl?locus=MET1), [ILV5/YLR355C](http://www.yeastgenome.org/cgi-bin/locus.fpl?locus=ILV5), [ARG7/YMR062C](http://www.yeastgenome.org/cgi-bin/locus.fpl?locus=ARG7), [ILV2/YMR108W](http://www.yeastgenome.org/cgi-bin/locus.fpl?locus=ILV2), [GAD1/YMR250W](http://www.yeastgenome.org/cgi-bin/locus.fpl?locus=GAD1), [LEU4/YNL104C](http://www.yeastgenome.org/cgi-bin/locus.fpl?locus=LEU4), [MET2/YNL277W](http://www.yeastgenome.org/cgi-bin/locus.fpl?locus=MET2), [BIO5/YNR056C](http://www.yeastgenome.org/cgi-bin/locus.fpl?locus=BIO5), [BIO4/YNR057C](http://www.yeastgenome.org/cgi-bin/locus.fpl?locus=BIO4), [BIO3/YNR058W](http://www.yeastgenome.org/cgi-bin/locus.fpl?locus=BIO3), [ARG8/YOL140W](http://www.yeastgenome.org/cgi-bin/locus.fpl?locus=ARG8), [LEU9/YOR108W](http://www.yeastgenome.org/cgi-bin/locus.fpl?locus=LEU9), [ORT1/YOR130C](http://www.yeastgenome.org/cgi-bin/locus.fpl?locus=ORT1), [HIS3/YOR202W](http://www.yeastgenome.org/cgi-bin/locus.fpl?locus=HIS3), [CPA1/YOR303W](http://www.yeastgenome.org/cgi-bin/locus.fpl?locus=CPA1), [PIP2/YOR363C](http://www.yeastgenome.org/cgi-bin/locus.fpl?locus=PIP2), [PDR12/YPL058C](http://www.yeastgenome.org/cgi-bin/locus.fpl?locus=PDR12), [EEB1/YPL095C](http://www.yeastgenome.org/cgi-bin/locus.fpl?locus=EEB1), [CIT3/YPR001W](http://www.yeastgenome.org/cgi-bin/locus.fpl?locus=CIT3), [MET16/YPR167C](http://www.yeastgenome.org/cgi-bin/locus.fpl?locus=MET16) |
| [*organic acid metabolic process*](http://www.yeastgenome.org/cgi-bin/GO/goTerm.pl?goid=6082) | 47 out of 302 genes, 15.6% | 345 out of 7167 genes, 4.8% | 2.22e-10 | [UGA2/YBR006W](http://www.yeastgenome.org/cgi-bin/locus.fpl?locus=UGA2), [PDX3/YBR035C](http://www.yeastgenome.org/cgi-bin/locus.fpl?locus=PDX3), [VID24/YBR105C](http://www.yeastgenome.org/cgi-bin/locus.fpl?locus=VID24), [HIS7/YBR248C](http://www.yeastgenome.org/cgi-bin/locus.fpl?locus=HIS7), [TRP4/YDR354W](http://www.yeastgenome.org/cgi-bin/locus.fpl?locus=TRP4), [YAT2/YER024W](http://www.yeastgenome.org/cgi-bin/locus.fpl?locus=YAT2), [HOM3/YER052C](http://www.yeastgenome.org/cgi-bin/locus.fpl?locus=HOM3), [ARG5,6/YER069W](http://www.yeastgenome.org/cgi-bin/locus.fpl?locus=ARG5), [ALD5/YER073W](http://www.yeastgenome.org/cgi-bin/locus.fpl?locus=ALD5), [MET6/YER091C](http://www.yeastgenome.org/cgi-bin/locus.fpl?locus=MET6), [LEU1/YGL009C](http://www.yeastgenome.org/cgi-bin/locus.fpl?locus=LEU1), [MET13/YGL125W](http://www.yeastgenome.org/cgi-bin/locus.fpl?locus=MET13), [STR3/YGL184C](http://www.yeastgenome.org/cgi-bin/locus.fpl?locus=STR3), [FOL2/YGR267C](http://www.yeastgenome.org/cgi-bin/locus.fpl?locus=FOL2), [ARG4/YHR018C](http://www.yeastgenome.org/cgi-bin/locus.fpl?locus=ARG4), [BAT1/YHR208W](http://www.yeastgenome.org/cgi-bin/locus.fpl?locus=BAT1), [VHR1/YIL056W](http://www.yeastgenome.org/cgi-bin/locus.fpl?locus=VHR1), [HIS5/YIL116W](http://www.yeastgenome.org/cgi-bin/locus.fpl?locus=HIS5), [MET28/YIR017C](http://www.yeastgenome.org/cgi-bin/locus.fpl?locus=MET28), [BNA3/YJL060W](http://www.yeastgenome.org/cgi-bin/locus.fpl?locus=BNA3), [ARG2/YJL071W](http://www.yeastgenome.org/cgi-bin/locus.fpl?locus=ARG2), [ARG3/YJL088W](http://www.yeastgenome.org/cgi-bin/locus.fpl?locus=ARG3), [SIP4/YJL089W](http://www.yeastgenome.org/cgi-bin/locus.fpl?locus=SIP4), [MET3/YJR010W](http://www.yeastgenome.org/cgi-bin/locus.fpl?locus=MET3), [ILV3/YJR016C](http://www.yeastgenome.org/cgi-bin/locus.fpl?locus=ILV3), [STR2/YJR130C](http://www.yeastgenome.org/cgi-bin/locus.fpl?locus=STR2), [MET14/YKL001C](http://www.yeastgenome.org/cgi-bin/locus.fpl?locus=MET14), [MET1/YKR069W](http://www.yeastgenome.org/cgi-bin/locus.fpl?locus=MET1), [ILV5/YLR355C](http://www.yeastgenome.org/cgi-bin/locus.fpl?locus=ILV5), [ARG7/YMR062C](http://www.yeastgenome.org/cgi-bin/locus.fpl?locus=ARG7), [ILV2/YMR108W](http://www.yeastgenome.org/cgi-bin/locus.fpl?locus=ILV2), [GAD1/YMR250W](http://www.yeastgenome.org/cgi-bin/locus.fpl?locus=GAD1), [LEU4/YNL104C](http://www.yeastgenome.org/cgi-bin/locus.fpl?locus=LEU4), [MET2/YNL277W](http://www.yeastgenome.org/cgi-bin/locus.fpl?locus=MET2), [BIO5/YNR056C](http://www.yeastgenome.org/cgi-bin/locus.fpl?locus=BIO5), [BIO4/YNR057C](http://www.yeastgenome.org/cgi-bin/locus.fpl?locus=BIO4), [BIO3/YNR058W](http://www.yeastgenome.org/cgi-bin/locus.fpl?locus=BIO3), [ARG8/YOL140W](http://www.yeastgenome.org/cgi-bin/locus.fpl?locus=ARG8), [LEU9/YOR108W](http://www.yeastgenome.org/cgi-bin/locus.fpl?locus=LEU9), [ORT1/YOR130C](http://www.yeastgenome.org/cgi-bin/locus.fpl?locus=ORT1), [HIS3/YOR202W](http://www.yeastgenome.org/cgi-bin/locus.fpl?locus=HIS3), [CPA1/YOR303W](http://www.yeastgenome.org/cgi-bin/locus.fpl?locus=CPA1), [PIP2/YOR363C](http://www.yeastgenome.org/cgi-bin/locus.fpl?locus=PIP2), [PDR12/YPL058C](http://www.yeastgenome.org/cgi-bin/locus.fpl?locus=PDR12), [EEB1/YPL095C](http://www.yeastgenome.org/cgi-bin/locus.fpl?locus=EEB1), [CIT3/YPR001W](http://www.yeastgenome.org/cgi-bin/locus.fpl?locus=CIT3), [MET16/YPR167C](http://www.yeastgenome.org/cgi-bin/locus.fpl?locus=MET16) |
| [*cellular ketone metabolic process*](http://www.yeastgenome.org/cgi-bin/GO/goTerm.pl?goid=42180) | 48 out of 302 genes, 15.9% | 359 out of 7167 genes, 5.0% | 2.46e-10 | [UGA2/YBR006W](http://www.yeastgenome.org/cgi-bin/locus.fpl?locus=UGA2), [PDX3/YBR035C](http://www.yeastgenome.org/cgi-bin/locus.fpl?locus=PDX3), [VID24/YBR105C](http://www.yeastgenome.org/cgi-bin/locus.fpl?locus=VID24), [HIS7/YBR248C](http://www.yeastgenome.org/cgi-bin/locus.fpl?locus=HIS7), [TRP4/YDR354W](http://www.yeastgenome.org/cgi-bin/locus.fpl?locus=TRP4), [YAT2/YER024W](http://www.yeastgenome.org/cgi-bin/locus.fpl?locus=YAT2), [HOM3/YER052C](http://www.yeastgenome.org/cgi-bin/locus.fpl?locus=HOM3), [ARG5,6/YER069W](http://www.yeastgenome.org/cgi-bin/locus.fpl?locus=ARG5), [ALD5/YER073W](http://www.yeastgenome.org/cgi-bin/locus.fpl?locus=ALD5), [MET6/YER091C](http://www.yeastgenome.org/cgi-bin/locus.fpl?locus=MET6), [LEU1/YGL009C](http://www.yeastgenome.org/cgi-bin/locus.fpl?locus=LEU1), [MET13/YGL125W](http://www.yeastgenome.org/cgi-bin/locus.fpl?locus=MET13), [STR3/YGL184C](http://www.yeastgenome.org/cgi-bin/locus.fpl?locus=STR3), [FOL2/YGR267C](http://www.yeastgenome.org/cgi-bin/locus.fpl?locus=FOL2), [ARG4/YHR018C](http://www.yeastgenome.org/cgi-bin/locus.fpl?locus=ARG4), [BAT1/YHR208W](http://www.yeastgenome.org/cgi-bin/locus.fpl?locus=BAT1), [VHR1/YIL056W](http://www.yeastgenome.org/cgi-bin/locus.fpl?locus=VHR1), [HIS5/YIL116W](http://www.yeastgenome.org/cgi-bin/locus.fpl?locus=HIS5), [MET28/YIR017C](http://www.yeastgenome.org/cgi-bin/locus.fpl?locus=MET28), [BNA3/YJL060W](http://www.yeastgenome.org/cgi-bin/locus.fpl?locus=BNA3), [ARG2/YJL071W](http://www.yeastgenome.org/cgi-bin/locus.fpl?locus=ARG2), [ARG3/YJL088W](http://www.yeastgenome.org/cgi-bin/locus.fpl?locus=ARG3), [SIP4/YJL089W](http://www.yeastgenome.org/cgi-bin/locus.fpl?locus=SIP4), [MET3/YJR010W](http://www.yeastgenome.org/cgi-bin/locus.fpl?locus=MET3), [ILV3/YJR016C](http://www.yeastgenome.org/cgi-bin/locus.fpl?locus=ILV3), [STR2/YJR130C](http://www.yeastgenome.org/cgi-bin/locus.fpl?locus=STR2), [MET14/YKL001C](http://www.yeastgenome.org/cgi-bin/locus.fpl?locus=MET14), [MET1/YKR069W](http://www.yeastgenome.org/cgi-bin/locus.fpl?locus=MET1), [ILV5/YLR355C](http://www.yeastgenome.org/cgi-bin/locus.fpl?locus=ILV5), [ARG7/YMR062C](http://www.yeastgenome.org/cgi-bin/locus.fpl?locus=ARG7), [ILV2/YMR108W](http://www.yeastgenome.org/cgi-bin/locus.fpl?locus=ILV2), [GAD1/YMR250W](http://www.yeastgenome.org/cgi-bin/locus.fpl?locus=GAD1), [LEU4/YNL104C](http://www.yeastgenome.org/cgi-bin/locus.fpl?locus=LEU4), [MET2/YNL277W](http://www.yeastgenome.org/cgi-bin/locus.fpl?locus=MET2), [BIO5/YNR056C](http://www.yeastgenome.org/cgi-bin/locus.fpl?locus=BIO5), [BIO4/YNR057C](http://www.yeastgenome.org/cgi-bin/locus.fpl?locus=BIO4), [BIO3/YNR058W](http://www.yeastgenome.org/cgi-bin/locus.fpl?locus=BIO3), [ARG8/YOL140W](http://www.yeastgenome.org/cgi-bin/locus.fpl?locus=ARG8), [LEU9/YOR108W](http://www.yeastgenome.org/cgi-bin/locus.fpl?locus=LEU9), [ORT1/YOR130C](http://www.yeastgenome.org/cgi-bin/locus.fpl?locus=ORT1), [HIS3/YOR202W](http://www.yeastgenome.org/cgi-bin/locus.fpl?locus=HIS3), [CPA1/YOR303W](http://www.yeastgenome.org/cgi-bin/locus.fpl?locus=CPA1), [PIP2/YOR363C](http://www.yeastgenome.org/cgi-bin/locus.fpl?locus=PIP2), [PDR12/YPL058C](http://www.yeastgenome.org/cgi-bin/locus.fpl?locus=PDR12), [EEB1/YPL095C](http://www.yeastgenome.org/cgi-bin/locus.fpl?locus=EEB1), [YAH1/YPL252C](http://www.yeastgenome.org/cgi-bin/locus.fpl?locus=YAH1), [CIT3/YPR001W](http://www.yeastgenome.org/cgi-bin/locus.fpl?locus=CIT3), [MET16/YPR167C](http://www.yeastgenome.org/cgi-bin/locus.fpl?locus=MET16) |
| [*cellular amino acid biosynthetic process*](http://www.yeastgenome.org/cgi-bin/GO/goTerm.pl?goid=8652) | 26 out of 302 genes, 8.6% | 112 out of 7167 genes, 1.6% | 3.11e-10 | [HIS7/YBR248C](http://www.yeastgenome.org/cgi-bin/locus.fpl?locus=HIS7), [TRP4/YDR354W](http://www.yeastgenome.org/cgi-bin/locus.fpl?locus=TRP4), [HOM3/YER052C](http://www.yeastgenome.org/cgi-bin/locus.fpl?locus=HOM3), [ARG5,6/YER069W](http://www.yeastgenome.org/cgi-bin/locus.fpl?locus=ARG5), [MET6/YER091C](http://www.yeastgenome.org/cgi-bin/locus.fpl?locus=MET6), [LEU1/YGL009C](http://www.yeastgenome.org/cgi-bin/locus.fpl?locus=LEU1), [MET13/YGL125W](http://www.yeastgenome.org/cgi-bin/locus.fpl?locus=MET13), [STR3/YGL184C](http://www.yeastgenome.org/cgi-bin/locus.fpl?locus=STR3), [ARG4/YHR018C](http://www.yeastgenome.org/cgi-bin/locus.fpl?locus=ARG4), [BAT1/YHR208W](http://www.yeastgenome.org/cgi-bin/locus.fpl?locus=BAT1), [HIS5/YIL116W](http://www.yeastgenome.org/cgi-bin/locus.fpl?locus=HIS5), [MET28/YIR017C](http://www.yeastgenome.org/cgi-bin/locus.fpl?locus=MET28), [ARG2/YJL071W](http://www.yeastgenome.org/cgi-bin/locus.fpl?locus=ARG2), [ARG3/YJL088W](http://www.yeastgenome.org/cgi-bin/locus.fpl?locus=ARG3), [ILV3/YJR016C](http://www.yeastgenome.org/cgi-bin/locus.fpl?locus=ILV3), [MET1/YKR069W](http://www.yeastgenome.org/cgi-bin/locus.fpl?locus=MET1), [ILV5/YLR355C](http://www.yeastgenome.org/cgi-bin/locus.fpl?locus=ILV5), [ARG7/YMR062C](http://www.yeastgenome.org/cgi-bin/locus.fpl?locus=ARG7), [ILV2/YMR108W](http://www.yeastgenome.org/cgi-bin/locus.fpl?locus=ILV2), [LEU4/YNL104C](http://www.yeastgenome.org/cgi-bin/locus.fpl?locus=LEU4), [MET2/YNL277W](http://www.yeastgenome.org/cgi-bin/locus.fpl?locus=MET2), [ARG8/YOL140W](http://www.yeastgenome.org/cgi-bin/locus.fpl?locus=ARG8), [LEU9/YOR108W](http://www.yeastgenome.org/cgi-bin/locus.fpl?locus=LEU9), [ORT1/YOR130C](http://www.yeastgenome.org/cgi-bin/locus.fpl?locus=ORT1), [HIS3/YOR202W](http://www.yeastgenome.org/cgi-bin/locus.fpl?locus=HIS3), [CPA1/YOR303W](http://www.yeastgenome.org/cgi-bin/locus.fpl?locus=CPA1) |
| [*amine biosynthetic process*](http://www.yeastgenome.org/cgi-bin/GO/goTerm.pl?goid=9309) | 26 out of 302 genes, 8.6% | 120 out of 7167 genes, 1.7% | 1.73e-09 | [HIS7/YBR248C](http://www.yeastgenome.org/cgi-bin/locus.fpl?locus=HIS7), [TRP4/YDR354W](http://www.yeastgenome.org/cgi-bin/locus.fpl?locus=TRP4), [HOM3/YER052C](http://www.yeastgenome.org/cgi-bin/locus.fpl?locus=HOM3), [ARG5,6/YER069W](http://www.yeastgenome.org/cgi-bin/locus.fpl?locus=ARG5), [MET6/YER091C](http://www.yeastgenome.org/cgi-bin/locus.fpl?locus=MET6), [LEU1/YGL009C](http://www.yeastgenome.org/cgi-bin/locus.fpl?locus=LEU1), [MET13/YGL125W](http://www.yeastgenome.org/cgi-bin/locus.fpl?locus=MET13), [STR3/YGL184C](http://www.yeastgenome.org/cgi-bin/locus.fpl?locus=STR3), [ARG4/YHR018C](http://www.yeastgenome.org/cgi-bin/locus.fpl?locus=ARG4), [BAT1/YHR208W](http://www.yeastgenome.org/cgi-bin/locus.fpl?locus=BAT1), [HIS5/YIL116W](http://www.yeastgenome.org/cgi-bin/locus.fpl?locus=HIS5), [MET28/YIR017C](http://www.yeastgenome.org/cgi-bin/locus.fpl?locus=MET28), [ARG2/YJL071W](http://www.yeastgenome.org/cgi-bin/locus.fpl?locus=ARG2), [ARG3/YJL088W](http://www.yeastgenome.org/cgi-bin/locus.fpl?locus=ARG3), [ILV3/YJR016C](http://www.yeastgenome.org/cgi-bin/locus.fpl?locus=ILV3), [MET1/YKR069W](http://www.yeastgenome.org/cgi-bin/locus.fpl?locus=MET1), [ILV5/YLR355C](http://www.yeastgenome.org/cgi-bin/locus.fpl?locus=ILV5), [ARG7/YMR062C](http://www.yeastgenome.org/cgi-bin/locus.fpl?locus=ARG7), [ILV2/YMR108W](http://www.yeastgenome.org/cgi-bin/locus.fpl?locus=ILV2), [LEU4/YNL104C](http://www.yeastgenome.org/cgi-bin/locus.fpl?locus=LEU4), [MET2/YNL277W](http://www.yeastgenome.org/cgi-bin/locus.fpl?locus=MET2), [ARG8/YOL140W](http://www.yeastgenome.org/cgi-bin/locus.fpl?locus=ARG8), [LEU9/YOR108W](http://www.yeastgenome.org/cgi-bin/locus.fpl?locus=LEU9), [ORT1/YOR130C](http://www.yeastgenome.org/cgi-bin/locus.fpl?locus=ORT1), [HIS3/YOR202W](http://www.yeastgenome.org/cgi-bin/locus.fpl?locus=HIS3), [CPA1/YOR303W](http://www.yeastgenome.org/cgi-bin/locus.fpl?locus=CPA1) |
| [*cellular nitrogen compound biosynthetic process*](http://www.yeastgenome.org/cgi-bin/GO/goTerm.pl?goid=44271) | 38 out of 302 genes, 12.6% | 253 out of 7167 genes, 3.5% | 2.57e-09 | [HIS7/YBR248C](http://www.yeastgenome.org/cgi-bin/locus.fpl?locus=HIS7), [RIB5/YBR256C](http://www.yeastgenome.org/cgi-bin/locus.fpl?locus=RIB5), [TRP4/YDR354W](http://www.yeastgenome.org/cgi-bin/locus.fpl?locus=TRP4), [RIB3/YDR487C](http://www.yeastgenome.org/cgi-bin/locus.fpl?locus=RIB3), [HOM3/YER052C](http://www.yeastgenome.org/cgi-bin/locus.fpl?locus=HOM3), [ARG5,6/YER069W](http://www.yeastgenome.org/cgi-bin/locus.fpl?locus=ARG5), [MET6/YER091C](http://www.yeastgenome.org/cgi-bin/locus.fpl?locus=MET6), [LEU1/YGL009C](http://www.yeastgenome.org/cgi-bin/locus.fpl?locus=LEU1), [PNC1/YGL037C](http://www.yeastgenome.org/cgi-bin/locus.fpl?locus=PNC1), [MET13/YGL125W](http://www.yeastgenome.org/cgi-bin/locus.fpl?locus=MET13), [STR3/YGL184C](http://www.yeastgenome.org/cgi-bin/locus.fpl?locus=STR3), [ARG4/YHR018C](http://www.yeastgenome.org/cgi-bin/locus.fpl?locus=ARG4), [BAT1/YHR208W](http://www.yeastgenome.org/cgi-bin/locus.fpl?locus=BAT1), [VHR1/YIL056W](http://www.yeastgenome.org/cgi-bin/locus.fpl?locus=VHR1), [HIS5/YIL116W](http://www.yeastgenome.org/cgi-bin/locus.fpl?locus=HIS5), [MET28/YIR017C](http://www.yeastgenome.org/cgi-bin/locus.fpl?locus=MET28), [ARG2/YJL071W](http://www.yeastgenome.org/cgi-bin/locus.fpl?locus=ARG2), [ARG3/YJL088W](http://www.yeastgenome.org/cgi-bin/locus.fpl?locus=ARG3), [ILV3/YJR016C](http://www.yeastgenome.org/cgi-bin/locus.fpl?locus=ILV3), [BNA1/YJR025C](http://www.yeastgenome.org/cgi-bin/locus.fpl?locus=BNA1), [MET1/YKR069W](http://www.yeastgenome.org/cgi-bin/locus.fpl?locus=MET1), [ILV5/YLR355C](http://www.yeastgenome.org/cgi-bin/locus.fpl?locus=ILV5), [ARG7/YMR062C](http://www.yeastgenome.org/cgi-bin/locus.fpl?locus=ARG7), [ILV2/YMR108W](http://www.yeastgenome.org/cgi-bin/locus.fpl?locus=ILV2), [URA10/YMR271C](http://www.yeastgenome.org/cgi-bin/locus.fpl?locus=URA10), [LEU4/YNL104C](http://www.yeastgenome.org/cgi-bin/locus.fpl?locus=LEU4), [NRK1/YNL129W](http://www.yeastgenome.org/cgi-bin/locus.fpl?locus=NRK1), [MET2/YNL277W](http://www.yeastgenome.org/cgi-bin/locus.fpl?locus=MET2), [BIO5/YNR056C](http://www.yeastgenome.org/cgi-bin/locus.fpl?locus=BIO5), [BIO4/YNR057C](http://www.yeastgenome.org/cgi-bin/locus.fpl?locus=BIO4), [BIO3/YNR058W](http://www.yeastgenome.org/cgi-bin/locus.fpl?locus=BIO3), [ARG8/YOL140W](http://www.yeastgenome.org/cgi-bin/locus.fpl?locus=ARG8), [LEU9/YOR108W](http://www.yeastgenome.org/cgi-bin/locus.fpl?locus=LEU9), [ORT1/YOR130C](http://www.yeastgenome.org/cgi-bin/locus.fpl?locus=ORT1), [HIS3/YOR202W](http://www.yeastgenome.org/cgi-bin/locus.fpl?locus=HIS3), [CPA1/YOR303W](http://www.yeastgenome.org/cgi-bin/locus.fpl?locus=CPA1), [POS5/YPL188W](http://www.yeastgenome.org/cgi-bin/locus.fpl?locus=POS5), [YAH1/YPL252C](http://www.yeastgenome.org/cgi-bin/locus.fpl?locus=YAH1) |
| [*small molecule biosynthetic process*](http://www.yeastgenome.org/cgi-bin/GO/goTerm.pl?goid=44283) | 44 out of 302 genes, 14.6% | 334 out of 7167 genes, 4.7% | 4.51e-09 | [VID24/YBR105C](http://www.yeastgenome.org/cgi-bin/locus.fpl?locus=VID24), [HIS7/YBR248C](http://www.yeastgenome.org/cgi-bin/locus.fpl?locus=HIS7), [RIB5/YBR256C](http://www.yeastgenome.org/cgi-bin/locus.fpl?locus=RIB5), [TRP4/YDR354W](http://www.yeastgenome.org/cgi-bin/locus.fpl?locus=TRP4), [RIB3/YDR487C](http://www.yeastgenome.org/cgi-bin/locus.fpl?locus=RIB3), [BUD16/YEL029C](http://www.yeastgenome.org/cgi-bin/locus.fpl?locus=BUD16), [HOM3/YER052C](http://www.yeastgenome.org/cgi-bin/locus.fpl?locus=HOM3), [ARG5,6/YER069W](http://www.yeastgenome.org/cgi-bin/locus.fpl?locus=ARG5), [ALD5/YER073W](http://www.yeastgenome.org/cgi-bin/locus.fpl?locus=ALD5), [MET6/YER091C](http://www.yeastgenome.org/cgi-bin/locus.fpl?locus=MET6), [LEU1/YGL009C](http://www.yeastgenome.org/cgi-bin/locus.fpl?locus=LEU1), [PNC1/YGL037C](http://www.yeastgenome.org/cgi-bin/locus.fpl?locus=PNC1), [MET13/YGL125W](http://www.yeastgenome.org/cgi-bin/locus.fpl?locus=MET13), [STR3/YGL184C](http://www.yeastgenome.org/cgi-bin/locus.fpl?locus=STR3), [ARG4/YHR018C](http://www.yeastgenome.org/cgi-bin/locus.fpl?locus=ARG4), [PAN5/YHR063C](http://www.yeastgenome.org/cgi-bin/locus.fpl?locus=PAN5), [BAT1/YHR208W](http://www.yeastgenome.org/cgi-bin/locus.fpl?locus=BAT1), [VHR1/YIL056W](http://www.yeastgenome.org/cgi-bin/locus.fpl?locus=VHR1), [HIS5/YIL116W](http://www.yeastgenome.org/cgi-bin/locus.fpl?locus=HIS5), [MET28/YIR017C](http://www.yeastgenome.org/cgi-bin/locus.fpl?locus=MET28), [BNA3/YJL060W](http://www.yeastgenome.org/cgi-bin/locus.fpl?locus=BNA3), [ARG2/YJL071W](http://www.yeastgenome.org/cgi-bin/locus.fpl?locus=ARG2), [ARG3/YJL088W](http://www.yeastgenome.org/cgi-bin/locus.fpl?locus=ARG3), [SIP4/YJL089W](http://www.yeastgenome.org/cgi-bin/locus.fpl?locus=SIP4), [ILV3/YJR016C](http://www.yeastgenome.org/cgi-bin/locus.fpl?locus=ILV3), [BNA1/YJR025C](http://www.yeastgenome.org/cgi-bin/locus.fpl?locus=BNA1), [MET1/YKR069W](http://www.yeastgenome.org/cgi-bin/locus.fpl?locus=MET1), [ILV5/YLR355C](http://www.yeastgenome.org/cgi-bin/locus.fpl?locus=ILV5), [FMS1/YMR020W](http://www.yeastgenome.org/cgi-bin/locus.fpl?locus=FMS1), [ARG7/YMR062C](http://www.yeastgenome.org/cgi-bin/locus.fpl?locus=ARG7), [ILV2/YMR108W](http://www.yeastgenome.org/cgi-bin/locus.fpl?locus=ILV2), [URA10/YMR271C](http://www.yeastgenome.org/cgi-bin/locus.fpl?locus=URA10), [LEU4/YNL104C](http://www.yeastgenome.org/cgi-bin/locus.fpl?locus=LEU4), [NRK1/YNL129W](http://www.yeastgenome.org/cgi-bin/locus.fpl?locus=NRK1), [MET2/YNL277W](http://www.yeastgenome.org/cgi-bin/locus.fpl?locus=MET2), [BIO5/YNR056C](http://www.yeastgenome.org/cgi-bin/locus.fpl?locus=BIO5), [BIO4/YNR057C](http://www.yeastgenome.org/cgi-bin/locus.fpl?locus=BIO4), [BIO3/YNR058W](http://www.yeastgenome.org/cgi-bin/locus.fpl?locus=BIO3), [ARG8/YOL140W](http://www.yeastgenome.org/cgi-bin/locus.fpl?locus=ARG8), [LEU9/YOR108W](http://www.yeastgenome.org/cgi-bin/locus.fpl?locus=LEU9), [ORT1/YOR130C](http://www.yeastgenome.org/cgi-bin/locus.fpl?locus=ORT1), [HIS3/YOR202W](http://www.yeastgenome.org/cgi-bin/locus.fpl?locus=HIS3), [CPA1/YOR303W](http://www.yeastgenome.org/cgi-bin/locus.fpl?locus=CPA1), [POS5/YPL188W](http://www.yeastgenome.org/cgi-bin/locus.fpl?locus=POS5) |
| [*cellular amino acid and derivative metabolic process*](http://www.yeastgenome.org/cgi-bin/GO/goTerm.pl?goid=6519) | 37 out of 302 genes, 12.3% | 255 out of 7167 genes, 3.6% | 1.45e-08 | [UGA2/YBR006W](http://www.yeastgenome.org/cgi-bin/locus.fpl?locus=UGA2), [HIS7/YBR248C](http://www.yeastgenome.org/cgi-bin/locus.fpl?locus=HIS7), [UGA3/YDL170W](http://www.yeastgenome.org/cgi-bin/locus.fpl?locus=UGA3), [TRP4/YDR354W](http://www.yeastgenome.org/cgi-bin/locus.fpl?locus=TRP4), [YAT2/YER024W](http://www.yeastgenome.org/cgi-bin/locus.fpl?locus=YAT2), [HOM3/YER052C](http://www.yeastgenome.org/cgi-bin/locus.fpl?locus=HOM3), [ARG5,6/YER069W](http://www.yeastgenome.org/cgi-bin/locus.fpl?locus=ARG5), [MET6/YER091C](http://www.yeastgenome.org/cgi-bin/locus.fpl?locus=MET6), [LEU1/YGL009C](http://www.yeastgenome.org/cgi-bin/locus.fpl?locus=LEU1), [MET13/YGL125W](http://www.yeastgenome.org/cgi-bin/locus.fpl?locus=MET13), [STR3/YGL184C](http://www.yeastgenome.org/cgi-bin/locus.fpl?locus=STR3), [ARG4/YHR018C](http://www.yeastgenome.org/cgi-bin/locus.fpl?locus=ARG4), [PAN5/YHR063C](http://www.yeastgenome.org/cgi-bin/locus.fpl?locus=PAN5), [BAT1/YHR208W](http://www.yeastgenome.org/cgi-bin/locus.fpl?locus=BAT1), [HIS5/YIL116W](http://www.yeastgenome.org/cgi-bin/locus.fpl?locus=HIS5), [MET28/YIR017C](http://www.yeastgenome.org/cgi-bin/locus.fpl?locus=MET28), [ARG2/YJL071W](http://www.yeastgenome.org/cgi-bin/locus.fpl?locus=ARG2), [ARG3/YJL088W](http://www.yeastgenome.org/cgi-bin/locus.fpl?locus=ARG3), [MET3/YJR010W](http://www.yeastgenome.org/cgi-bin/locus.fpl?locus=MET3), [ILV3/YJR016C](http://www.yeastgenome.org/cgi-bin/locus.fpl?locus=ILV3), [STR2/YJR130C](http://www.yeastgenome.org/cgi-bin/locus.fpl?locus=STR2), [MET14/YKL001C](http://www.yeastgenome.org/cgi-bin/locus.fpl?locus=MET14), [SRY1/YKL218C](http://www.yeastgenome.org/cgi-bin/locus.fpl?locus=SRY1), [MET1/YKR069W](http://www.yeastgenome.org/cgi-bin/locus.fpl?locus=MET1), [ILV5/YLR355C](http://www.yeastgenome.org/cgi-bin/locus.fpl?locus=ILV5), [FMS1/YMR020W](http://www.yeastgenome.org/cgi-bin/locus.fpl?locus=FMS1), [ARG7/YMR062C](http://www.yeastgenome.org/cgi-bin/locus.fpl?locus=ARG7), [ILV2/YMR108W](http://www.yeastgenome.org/cgi-bin/locus.fpl?locus=ILV2), [GAD1/YMR250W](http://www.yeastgenome.org/cgi-bin/locus.fpl?locus=GAD1), [LEU4/YNL104C](http://www.yeastgenome.org/cgi-bin/locus.fpl?locus=LEU4), [MET2/YNL277W](http://www.yeastgenome.org/cgi-bin/locus.fpl?locus=MET2), [ARG8/YOL140W](http://www.yeastgenome.org/cgi-bin/locus.fpl?locus=ARG8), [LEU9/YOR108W](http://www.yeastgenome.org/cgi-bin/locus.fpl?locus=LEU9), [ORT1/YOR130C](http://www.yeastgenome.org/cgi-bin/locus.fpl?locus=ORT1), [HIS3/YOR202W](http://www.yeastgenome.org/cgi-bin/locus.fpl?locus=HIS3), [CPA1/YOR303W](http://www.yeastgenome.org/cgi-bin/locus.fpl?locus=CPA1), [MET16/YPR167C](http://www.yeastgenome.org/cgi-bin/locus.fpl?locus=MET16) |
| [*cellular amine metabolic process*](http://www.yeastgenome.org/cgi-bin/GO/goTerm.pl?goid=44106) | 34 out of 302 genes, 11.3% | 232 out of 7167 genes, 3.2% | 7.68e-08 | [UGA2/YBR006W](http://www.yeastgenome.org/cgi-bin/locus.fpl?locus=UGA2), [HIS7/YBR248C](http://www.yeastgenome.org/cgi-bin/locus.fpl?locus=HIS7), [TRP4/YDR354W](http://www.yeastgenome.org/cgi-bin/locus.fpl?locus=TRP4), [YAT2/YER024W](http://www.yeastgenome.org/cgi-bin/locus.fpl?locus=YAT2), [HOM3/YER052C](http://www.yeastgenome.org/cgi-bin/locus.fpl?locus=HOM3), [ARG5,6/YER069W](http://www.yeastgenome.org/cgi-bin/locus.fpl?locus=ARG5), [MET6/YER091C](http://www.yeastgenome.org/cgi-bin/locus.fpl?locus=MET6), [LEU1/YGL009C](http://www.yeastgenome.org/cgi-bin/locus.fpl?locus=LEU1), [MET13/YGL125W](http://www.yeastgenome.org/cgi-bin/locus.fpl?locus=MET13), [STR3/YGL184C](http://www.yeastgenome.org/cgi-bin/locus.fpl?locus=STR3), [ARG4/YHR018C](http://www.yeastgenome.org/cgi-bin/locus.fpl?locus=ARG4), [BAT1/YHR208W](http://www.yeastgenome.org/cgi-bin/locus.fpl?locus=BAT1), [HIS5/YIL116W](http://www.yeastgenome.org/cgi-bin/locus.fpl?locus=HIS5), [MET28/YIR017C](http://www.yeastgenome.org/cgi-bin/locus.fpl?locus=MET28), [ARG2/YJL071W](http://www.yeastgenome.org/cgi-bin/locus.fpl?locus=ARG2), [ARG3/YJL088W](http://www.yeastgenome.org/cgi-bin/locus.fpl?locus=ARG3), [MET3/YJR010W](http://www.yeastgenome.org/cgi-bin/locus.fpl?locus=MET3), [ILV3/YJR016C](http://www.yeastgenome.org/cgi-bin/locus.fpl?locus=ILV3), [STR2/YJR130C](http://www.yeastgenome.org/cgi-bin/locus.fpl?locus=STR2), [MET14/YKL001C](http://www.yeastgenome.org/cgi-bin/locus.fpl?locus=MET14), [MET1/YKR069W](http://www.yeastgenome.org/cgi-bin/locus.fpl?locus=MET1), [ILV5/YLR355C](http://www.yeastgenome.org/cgi-bin/locus.fpl?locus=ILV5), [FMS1/YMR020W](http://www.yeastgenome.org/cgi-bin/locus.fpl?locus=FMS1), [ARG7/YMR062C](http://www.yeastgenome.org/cgi-bin/locus.fpl?locus=ARG7), [ILV2/YMR108W](http://www.yeastgenome.org/cgi-bin/locus.fpl?locus=ILV2), [GAD1/YMR250W](http://www.yeastgenome.org/cgi-bin/locus.fpl?locus=GAD1), [LEU4/YNL104C](http://www.yeastgenome.org/cgi-bin/locus.fpl?locus=LEU4), [MET2/YNL277W](http://www.yeastgenome.org/cgi-bin/locus.fpl?locus=MET2), [ARG8/YOL140W](http://www.yeastgenome.org/cgi-bin/locus.fpl?locus=ARG8), [LEU9/YOR108W](http://www.yeastgenome.org/cgi-bin/locus.fpl?locus=LEU9), [ORT1/YOR130C](http://www.yeastgenome.org/cgi-bin/locus.fpl?locus=ORT1), [HIS3/YOR202W](http://www.yeastgenome.org/cgi-bin/locus.fpl?locus=HIS3), [CPA1/YOR303W](http://www.yeastgenome.org/cgi-bin/locus.fpl?locus=CPA1), [MET16/YPR167C](http://www.yeastgenome.org/cgi-bin/locus.fpl?locus=MET16) |
| [*cellular amino acid metabolic process*](http://www.yeastgenome.org/cgi-bin/GO/goTerm.pl?goid=6520) | 32 out of 302 genes, 10.6% | 210 out of 7167 genes, 2.9% | 1.00e-07 | [UGA2/YBR006W](http://www.yeastgenome.org/cgi-bin/locus.fpl?locus=UGA2), [HIS7/YBR248C](http://www.yeastgenome.org/cgi-bin/locus.fpl?locus=HIS7), [TRP4/YDR354W](http://www.yeastgenome.org/cgi-bin/locus.fpl?locus=TRP4), [HOM3/YER052C](http://www.yeastgenome.org/cgi-bin/locus.fpl?locus=HOM3), [ARG5,6/YER069W](http://www.yeastgenome.org/cgi-bin/locus.fpl?locus=ARG5), [MET6/YER091C](http://www.yeastgenome.org/cgi-bin/locus.fpl?locus=MET6), [LEU1/YGL009C](http://www.yeastgenome.org/cgi-bin/locus.fpl?locus=LEU1), [MET13/YGL125W](http://www.yeastgenome.org/cgi-bin/locus.fpl?locus=MET13), [STR3/YGL184C](http://www.yeastgenome.org/cgi-bin/locus.fpl?locus=STR3), [ARG4/YHR018C](http://www.yeastgenome.org/cgi-bin/locus.fpl?locus=ARG4), [BAT1/YHR208W](http://www.yeastgenome.org/cgi-bin/locus.fpl?locus=BAT1), [HIS5/YIL116W](http://www.yeastgenome.org/cgi-bin/locus.fpl?locus=HIS5), [MET28/YIR017C](http://www.yeastgenome.org/cgi-bin/locus.fpl?locus=MET28), [ARG2/YJL071W](http://www.yeastgenome.org/cgi-bin/locus.fpl?locus=ARG2), [ARG3/YJL088W](http://www.yeastgenome.org/cgi-bin/locus.fpl?locus=ARG3), [MET3/YJR010W](http://www.yeastgenome.org/cgi-bin/locus.fpl?locus=MET3), [ILV3/YJR016C](http://www.yeastgenome.org/cgi-bin/locus.fpl?locus=ILV3), [STR2/YJR130C](http://www.yeastgenome.org/cgi-bin/locus.fpl?locus=STR2), [MET14/YKL001C](http://www.yeastgenome.org/cgi-bin/locus.fpl?locus=MET14), [MET1/YKR069W](http://www.yeastgenome.org/cgi-bin/locus.fpl?locus=MET1), [ILV5/YLR355C](http://www.yeastgenome.org/cgi-bin/locus.fpl?locus=ILV5), [ARG7/YMR062C](http://www.yeastgenome.org/cgi-bin/locus.fpl?locus=ARG7), [ILV2/YMR108W](http://www.yeastgenome.org/cgi-bin/locus.fpl?locus=ILV2), [GAD1/YMR250W](http://www.yeastgenome.org/cgi-bin/locus.fpl?locus=GAD1), [LEU4/YNL104C](http://www.yeastgenome.org/cgi-bin/locus.fpl?locus=LEU4), [MET2/YNL277W](http://www.yeastgenome.org/cgi-bin/locus.fpl?locus=MET2), [ARG8/YOL140W](http://www.yeastgenome.org/cgi-bin/locus.fpl?locus=ARG8), [LEU9/YOR108W](http://www.yeastgenome.org/cgi-bin/locus.fpl?locus=LEU9), [ORT1/YOR130C](http://www.yeastgenome.org/cgi-bin/locus.fpl?locus=ORT1), [HIS3/YOR202W](http://www.yeastgenome.org/cgi-bin/locus.fpl?locus=HIS3), [CPA1/YOR303W](http://www.yeastgenome.org/cgi-bin/locus.fpl?locus=CPA1), [MET16/YPR167C](http://www.yeastgenome.org/cgi-bin/locus.fpl?locus=MET16) |
| [*amine metabolic process*](http://www.yeastgenome.org/cgi-bin/GO/goTerm.pl?goid=9308) | 35 out of 302 genes, 11.6% | 262 out of 7167 genes, 3.7% | 5.41e-07 | [UGA2/YBR006W](http://www.yeastgenome.org/cgi-bin/locus.fpl?locus=UGA2), [HIS7/YBR248C](http://www.yeastgenome.org/cgi-bin/locus.fpl?locus=HIS7), [UGA3/YDL170W](http://www.yeastgenome.org/cgi-bin/locus.fpl?locus=UGA3), [TRP4/YDR354W](http://www.yeastgenome.org/cgi-bin/locus.fpl?locus=TRP4), [YAT2/YER024W](http://www.yeastgenome.org/cgi-bin/locus.fpl?locus=YAT2), [HOM3/YER052C](http://www.yeastgenome.org/cgi-bin/locus.fpl?locus=HOM3), [ARG5,6/YER069W](http://www.yeastgenome.org/cgi-bin/locus.fpl?locus=ARG5), [MET6/YER091C](http://www.yeastgenome.org/cgi-bin/locus.fpl?locus=MET6), [LEU1/YGL009C](http://www.yeastgenome.org/cgi-bin/locus.fpl?locus=LEU1), [MET13/YGL125W](http://www.yeastgenome.org/cgi-bin/locus.fpl?locus=MET13), [STR3/YGL184C](http://www.yeastgenome.org/cgi-bin/locus.fpl?locus=STR3), [ARG4/YHR018C](http://www.yeastgenome.org/cgi-bin/locus.fpl?locus=ARG4), [BAT1/YHR208W](http://www.yeastgenome.org/cgi-bin/locus.fpl?locus=BAT1), [HIS5/YIL116W](http://www.yeastgenome.org/cgi-bin/locus.fpl?locus=HIS5), [MET28/YIR017C](http://www.yeastgenome.org/cgi-bin/locus.fpl?locus=MET28), [ARG2/YJL071W](http://www.yeastgenome.org/cgi-bin/locus.fpl?locus=ARG2), [ARG3/YJL088W](http://www.yeastgenome.org/cgi-bin/locus.fpl?locus=ARG3), [MET3/YJR010W](http://www.yeastgenome.org/cgi-bin/locus.fpl?locus=MET3), [ILV3/YJR016C](http://www.yeastgenome.org/cgi-bin/locus.fpl?locus=ILV3), [STR2/YJR130C](http://www.yeastgenome.org/cgi-bin/locus.fpl?locus=STR2), [MET14/YKL001C](http://www.yeastgenome.org/cgi-bin/locus.fpl?locus=MET14), [MET1/YKR069W](http://www.yeastgenome.org/cgi-bin/locus.fpl?locus=MET1), [ILV5/YLR355C](http://www.yeastgenome.org/cgi-bin/locus.fpl?locus=ILV5), [FMS1/YMR020W](http://www.yeastgenome.org/cgi-bin/locus.fpl?locus=FMS1), [ARG7/YMR062C](http://www.yeastgenome.org/cgi-bin/locus.fpl?locus=ARG7), [ILV2/YMR108W](http://www.yeastgenome.org/cgi-bin/locus.fpl?locus=ILV2), [GAD1/YMR250W](http://www.yeastgenome.org/cgi-bin/locus.fpl?locus=GAD1), [LEU4/YNL104C](http://www.yeastgenome.org/cgi-bin/locus.fpl?locus=LEU4), [MET2/YNL277W](http://www.yeastgenome.org/cgi-bin/locus.fpl?locus=MET2), [ARG8/YOL140W](http://www.yeastgenome.org/cgi-bin/locus.fpl?locus=ARG8), [LEU9/YOR108W](http://www.yeastgenome.org/cgi-bin/locus.fpl?locus=LEU9), [ORT1/YOR130C](http://www.yeastgenome.org/cgi-bin/locus.fpl?locus=ORT1), [HIS3/YOR202W](http://www.yeastgenome.org/cgi-bin/locus.fpl?locus=HIS3), [CPA1/YOR303W](http://www.yeastgenome.org/cgi-bin/locus.fpl?locus=CPA1), [MET16/YPR167C](http://www.yeastgenome.org/cgi-bin/locus.fpl?locus=MET16) |
| [*arginine biosynthetic process*](http://www.yeastgenome.org/cgi-bin/GO/goTerm.pl?goid=6526) | 8 out of 302 genes, 2.6% | 11 out of 7167 genes, 0.2% | 9.05e-07 | [ARG5,6/YER069W](http://www.yeastgenome.org/cgi-bin/locus.fpl?locus=ARG5), [ARG4/YHR018C](http://www.yeastgenome.org/cgi-bin/locus.fpl?locus=ARG4), [ARG2/YJL071W](http://www.yeastgenome.org/cgi-bin/locus.fpl?locus=ARG2), [ARG3/YJL088W](http://www.yeastgenome.org/cgi-bin/locus.fpl?locus=ARG3), [ARG7/YMR062C](http://www.yeastgenome.org/cgi-bin/locus.fpl?locus=ARG7), [ARG8/YOL140W](http://www.yeastgenome.org/cgi-bin/locus.fpl?locus=ARG8), [ORT1/YOR130C](http://www.yeastgenome.org/cgi-bin/locus.fpl?locus=ORT1), [CPA1/YOR303W](http://www.yeastgenome.org/cgi-bin/locus.fpl?locus=CPA1) |
| [*sulfur metabolic process*](http://www.yeastgenome.org/cgi-bin/GO/goTerm.pl?goid=6790) | 19 out of 302 genes, 6.3% | 96 out of 7167 genes, 1.3% | 8.38e-06 | [HOM3/YER052C](http://www.yeastgenome.org/cgi-bin/locus.fpl?locus=HOM3), [MET6/YER091C](http://www.yeastgenome.org/cgi-bin/locus.fpl?locus=MET6), [MET10/YFR030W](http://www.yeastgenome.org/cgi-bin/locus.fpl?locus=MET10), [MET13/YGL125W](http://www.yeastgenome.org/cgi-bin/locus.fpl?locus=MET13), [STR3/YGL184C](http://www.yeastgenome.org/cgi-bin/locus.fpl?locus=STR3), [VHR1/YIL056W](http://www.yeastgenome.org/cgi-bin/locus.fpl?locus=VHR1), [MET28/YIR017C](http://www.yeastgenome.org/cgi-bin/locus.fpl?locus=MET28), [MET3/YJR010W](http://www.yeastgenome.org/cgi-bin/locus.fpl?locus=MET3), [STR2/YJR130C](http://www.yeastgenome.org/cgi-bin/locus.fpl?locus=STR2), [MET5/YJR137C](http://www.yeastgenome.org/cgi-bin/locus.fpl?locus=MET5), [MET14/YKL001C](http://www.yeastgenome.org/cgi-bin/locus.fpl?locus=MET14), [MET1/YKR069W](http://www.yeastgenome.org/cgi-bin/locus.fpl?locus=MET1), [JLP1/YLL057C](http://www.yeastgenome.org/cgi-bin/locus.fpl?locus=JLP1), [YLL058W](http://www.yeastgenome.org/cgi-bin/locus.fpl?locus=YLL058W), [MET2/YNL277W](http://www.yeastgenome.org/cgi-bin/locus.fpl?locus=MET2), [BIO5/YNR056C](http://www.yeastgenome.org/cgi-bin/locus.fpl?locus=BIO5), [BIO4/YNR057C](http://www.yeastgenome.org/cgi-bin/locus.fpl?locus=BIO4), [BIO3/YNR058W](http://www.yeastgenome.org/cgi-bin/locus.fpl?locus=BIO3), [MET16/YPR167C](http://www.yeastgenome.org/cgi-bin/locus.fpl?locus=MET16) |
| [*response to chemical stimulus*](http://www.yeastgenome.org/cgi-bin/GO/goTerm.pl?goid=42221) | 36 out of 302 genes, 11.9% | 317 out of 7167 genes, 4.4% | 2.53e-05 | [UGA2/YBR006W](http://www.yeastgenome.org/cgi-bin/locus.fpl?locus=UGA2), [FUS1/YCL027W](http://www.yeastgenome.org/cgi-bin/locus.fpl?locus=FUS1), [SNQ2/YDR011W](http://www.yeastgenome.org/cgi-bin/locus.fpl?locus=SNQ2), [AFR1/YDR085C](http://www.yeastgenome.org/cgi-bin/locus.fpl?locus=AFR1), [STE5/YDR103W](http://www.yeastgenome.org/cgi-bin/locus.fpl?locus=STE5), [GLN3/YER040W](http://www.yeastgenome.org/cgi-bin/locus.fpl?locus=GLN3), [DSE1/YER124C](http://www.yeastgenome.org/cgi-bin/locus.fpl?locus=DSE1), [SPI1/YER150W](http://www.yeastgenome.org/cgi-bin/locus.fpl?locus=SPI1), [HSP12/YFL014W](http://www.yeastgenome.org/cgi-bin/locus.fpl?locus=HSP12), [GAT1/YFL021W](http://www.yeastgenome.org/cgi-bin/locus.fpl?locus=GAT1), [STE2/YFL026W](http://www.yeastgenome.org/cgi-bin/locus.fpl?locus=STE2), [AGA2/YGL032C](http://www.yeastgenome.org/cgi-bin/locus.fpl?locus=AGA2), [CTT1/YGR088W](http://www.yeastgenome.org/cgi-bin/locus.fpl?locus=CTT1), [YOR1/YGR281W](http://www.yeastgenome.org/cgi-bin/locus.fpl?locus=YOR1), [YHI9/YHR029C](http://www.yeastgenome.org/cgi-bin/locus.fpl?locus=YHI9), [CUP1-1/YHR053C](http://www.yeastgenome.org/cgi-bin/locus.fpl?locus=CUP1), [CUP1-2/YHR055C](http://www.yeastgenome.org/cgi-bin/locus.fpl?locus=CUP1), [QDR1/YIL120W](http://www.yeastgenome.org/cgi-bin/locus.fpl?locus=QDR1), [QDR2/YIL121W](http://www.yeastgenome.org/cgi-bin/locus.fpl?locus=QDR2), [YJL055W](http://www.yeastgenome.org/cgi-bin/locus.fpl?locus=YJL055W), [YJL144W](http://www.yeastgenome.org/cgi-bin/locus.fpl?locus=YJL144W), [MCR1/YKL150W](http://www.yeastgenome.org/cgi-bin/locus.fpl?locus=MCR1), [HSP104/YLL026W](http://www.yeastgenome.org/cgi-bin/locus.fpl?locus=HSP104), [MID2/YLR332W](http://www.yeastgenome.org/cgi-bin/locus.fpl?locus=MID2), [SST2/YLR452C](http://www.yeastgenome.org/cgi-bin/locus.fpl?locus=SST2), [ATR1/YML116W](http://www.yeastgenome.org/cgi-bin/locus.fpl?locus=ATR1), [GAD1/YMR250W](http://www.yeastgenome.org/cgi-bin/locus.fpl?locus=GAD1), [NCE103/YNL036W](http://www.yeastgenome.org/cgi-bin/locus.fpl?locus=NCE103), [MFA2/YNL145W](http://www.yeastgenome.org/cgi-bin/locus.fpl?locus=MFA2), [URE2/YNL229C](http://www.yeastgenome.org/cgi-bin/locus.fpl?locus=URE2), [ZWF1/YNL241C](http://www.yeastgenome.org/cgi-bin/locus.fpl?locus=ZWF1), [PRM1/YNL279W](http://www.yeastgenome.org/cgi-bin/locus.fpl?locus=PRM1), [AGA1/YNR044W](http://www.yeastgenome.org/cgi-bin/locus.fpl?locus=AGA1), [AIF1/YNR074C](http://www.yeastgenome.org/cgi-bin/locus.fpl?locus=AIF1), [GCY1/YOR120W](http://www.yeastgenome.org/cgi-bin/locus.fpl?locus=GCY1), [POS5/YPL188W](http://www.yeastgenome.org/cgi-bin/locus.fpl?locus=POS5) |
| [*arginine metabolic process*](http://www.yeastgenome.org/cgi-bin/GO/goTerm.pl?goid=6525) | 8 out of 302 genes, 2.6% | 16 out of 7167 genes, 0.2% | 5.86e-05 | [ARG5,6/YER069W](http://www.yeastgenome.org/cgi-bin/locus.fpl?locus=ARG5), [ARG4/YHR018C](http://www.yeastgenome.org/cgi-bin/locus.fpl?locus=ARG4), [ARG2/YJL071W](http://www.yeastgenome.org/cgi-bin/locus.fpl?locus=ARG2), [ARG3/YJL088W](http://www.yeastgenome.org/cgi-bin/locus.fpl?locus=ARG3), [ARG7/YMR062C](http://www.yeastgenome.org/cgi-bin/locus.fpl?locus=ARG7), [ARG8/YOL140W](http://www.yeastgenome.org/cgi-bin/locus.fpl?locus=ARG8), [ORT1/YOR130C](http://www.yeastgenome.org/cgi-bin/locus.fpl?locus=ORT1), [CPA1/YOR303W](http://www.yeastgenome.org/cgi-bin/locus.fpl?locus=CPA1) |
| [*sulfur amino acid metabolic process*](http://www.yeastgenome.org/cgi-bin/GO/goTerm.pl?goid=96) | 11 out of 302 genes, 3.6% | 37 out of 7167 genes, 0.5% | 0.00013 | [HOM3/YER052C](http://www.yeastgenome.org/cgi-bin/locus.fpl?locus=HOM3), [MET6/YER091C](http://www.yeastgenome.org/cgi-bin/locus.fpl?locus=MET6), [MET13/YGL125W](http://www.yeastgenome.org/cgi-bin/locus.fpl?locus=MET13), [STR3/YGL184C](http://www.yeastgenome.org/cgi-bin/locus.fpl?locus=STR3), [MET28/YIR017C](http://www.yeastgenome.org/cgi-bin/locus.fpl?locus=MET28), [MET3/YJR010W](http://www.yeastgenome.org/cgi-bin/locus.fpl?locus=MET3), [STR2/YJR130C](http://www.yeastgenome.org/cgi-bin/locus.fpl?locus=STR2), [MET14/YKL001C](http://www.yeastgenome.org/cgi-bin/locus.fpl?locus=MET14), [MET1/YKR069W](http://www.yeastgenome.org/cgi-bin/locus.fpl?locus=MET1), [MET2/YNL277W](http://www.yeastgenome.org/cgi-bin/locus.fpl?locus=MET2), [MET16/YPR167C](http://www.yeastgenome.org/cgi-bin/locus.fpl?locus=MET16) |
| [*branched chain family amino acid biosynthetic process*](http://www.yeastgenome.org/cgi-bin/GO/goTerm.pl?goid=9082) | 7 out of 302 genes, 2.3% | 13 out of 7167 genes, 0.2% | 0.00021 | [LEU1/YGL009C](http://www.yeastgenome.org/cgi-bin/locus.fpl?locus=LEU1), [BAT1/YHR208W](http://www.yeastgenome.org/cgi-bin/locus.fpl?locus=BAT1), [ILV3/YJR016C](http://www.yeastgenome.org/cgi-bin/locus.fpl?locus=ILV3), [ILV5/YLR355C](http://www.yeastgenome.org/cgi-bin/locus.fpl?locus=ILV5), [ILV2/YMR108W](http://www.yeastgenome.org/cgi-bin/locus.fpl?locus=ILV2), [LEU4/YNL104C](http://www.yeastgenome.org/cgi-bin/locus.fpl?locus=LEU4), [LEU9/YOR108W](http://www.yeastgenome.org/cgi-bin/locus.fpl?locus=LEU9) |
| [*methionine metabolic process*](http://www.yeastgenome.org/cgi-bin/GO/goTerm.pl?goid=6555) | 9 out of 302 genes, 3.0% | 26 out of 7167 genes, 0.4% | 0.00042 | [HOM3/YER052C](http://www.yeastgenome.org/cgi-bin/locus.fpl?locus=HOM3), [MET6/YER091C](http://www.yeastgenome.org/cgi-bin/locus.fpl?locus=MET6), [MET13/YGL125W](http://www.yeastgenome.org/cgi-bin/locus.fpl?locus=MET13), [STR3/YGL184C](http://www.yeastgenome.org/cgi-bin/locus.fpl?locus=STR3), [MET3/YJR010W](http://www.yeastgenome.org/cgi-bin/locus.fpl?locus=MET3), [MET14/YKL001C](http://www.yeastgenome.org/cgi-bin/locus.fpl?locus=MET14), [MET1/YKR069W](http://www.yeastgenome.org/cgi-bin/locus.fpl?locus=MET1), [MET2/YNL277W](http://www.yeastgenome.org/cgi-bin/locus.fpl?locus=MET2), [MET16/YPR167C](http://www.yeastgenome.org/cgi-bin/locus.fpl?locus=MET16) |
| [*sulfate assimilation*](http://www.yeastgenome.org/cgi-bin/GO/goTerm.pl?goid=103) | 6 out of 302 genes, 2.0% | 10 out of 7167 genes, 0.1% | 0.00065 | [MET10/YFR030W](http://www.yeastgenome.org/cgi-bin/locus.fpl?locus=MET10), [MET3/YJR010W](http://www.yeastgenome.org/cgi-bin/locus.fpl?locus=MET3), [MET5/YJR137C](http://www.yeastgenome.org/cgi-bin/locus.fpl?locus=MET5), [MET14/YKL001C](http://www.yeastgenome.org/cgi-bin/locus.fpl?locus=MET14), [MET1/YKR069W](http://www.yeastgenome.org/cgi-bin/locus.fpl?locus=MET1), [MET16/YPR167C](http://www.yeastgenome.org/cgi-bin/locus.fpl?locus=MET16) |
| [*response to reactive oxygen species*](http://www.yeastgenome.org/cgi-bin/GO/goTerm.pl?goid=302) | 7 out of 302 genes, 2.3% | 16 out of 7167 genes, 0.2% | 0.00122 | [SNQ2/YDR011W](http://www.yeastgenome.org/cgi-bin/locus.fpl?locus=SNQ2), [CTT1/YGR088W](http://www.yeastgenome.org/cgi-bin/locus.fpl?locus=CTT1), [CUP1-1/YHR053C](http://www.yeastgenome.org/cgi-bin/locus.fpl?locus=CUP1), [CUP1-2/YHR055C](http://www.yeastgenome.org/cgi-bin/locus.fpl?locus=CUP1), [HSP104/YLL026W](http://www.yeastgenome.org/cgi-bin/locus.fpl?locus=HSP104), [ZWF1/YNL241C](http://www.yeastgenome.org/cgi-bin/locus.fpl?locus=ZWF1), [AIF1/YNR074C](http://www.yeastgenome.org/cgi-bin/locus.fpl?locus=AIF1) |
| [*vitamin metabolic process*](http://www.yeastgenome.org/cgi-bin/GO/goTerm.pl?goid=6766) | 13 out of 302 genes, 4.3% | 65 out of 7167 genes, 0.9% | 0.00157 | [RIB5/YBR256C](http://www.yeastgenome.org/cgi-bin/locus.fpl?locus=RIB5), [RIB3/YDR487C](http://www.yeastgenome.org/cgi-bin/locus.fpl?locus=RIB3), [BUD16/YEL029C](http://www.yeastgenome.org/cgi-bin/locus.fpl?locus=BUD16), [YAT2/YER024W](http://www.yeastgenome.org/cgi-bin/locus.fpl?locus=YAT2), [PNC1/YGL037C](http://www.yeastgenome.org/cgi-bin/locus.fpl?locus=PNC1), [VHR1/YIL056W](http://www.yeastgenome.org/cgi-bin/locus.fpl?locus=VHR1), [BNA1/YJR025C](http://www.yeastgenome.org/cgi-bin/locus.fpl?locus=BNA1), [SNZ1/YMR096W](http://www.yeastgenome.org/cgi-bin/locus.fpl?locus=SNZ1), [NRK1/YNL129W](http://www.yeastgenome.org/cgi-bin/locus.fpl?locus=NRK1), [BIO5/YNR056C](http://www.yeastgenome.org/cgi-bin/locus.fpl?locus=BIO5), [BIO4/YNR057C](http://www.yeastgenome.org/cgi-bin/locus.fpl?locus=BIO4), [BIO3/YNR058W](http://www.yeastgenome.org/cgi-bin/locus.fpl?locus=BIO3), [POS5/YPL188W](http://www.yeastgenome.org/cgi-bin/locus.fpl?locus=POS5) |
| [*water-soluble vitamin metabolic process*](http://www.yeastgenome.org/cgi-bin/GO/goTerm.pl?goid=6767) | 13 out of 302 genes, 4.3% | 65 out of 7167 genes, 0.9% | 0.00157 | [RIB5/YBR256C](http://www.yeastgenome.org/cgi-bin/locus.fpl?locus=RIB5), [RIB3/YDR487C](http://www.yeastgenome.org/cgi-bin/locus.fpl?locus=RIB3), [BUD16/YEL029C](http://www.yeastgenome.org/cgi-bin/locus.fpl?locus=BUD16), [YAT2/YER024W](http://www.yeastgenome.org/cgi-bin/locus.fpl?locus=YAT2), [PNC1/YGL037C](http://www.yeastgenome.org/cgi-bin/locus.fpl?locus=PNC1), [VHR1/YIL056W](http://www.yeastgenome.org/cgi-bin/locus.fpl?locus=VHR1), [BNA1/YJR025C](http://www.yeastgenome.org/cgi-bin/locus.fpl?locus=BNA1), [SNZ1/YMR096W](http://www.yeastgenome.org/cgi-bin/locus.fpl?locus=SNZ1), [NRK1/YNL129W](http://www.yeastgenome.org/cgi-bin/locus.fpl?locus=NRK1), [BIO5/YNR056C](http://www.yeastgenome.org/cgi-bin/locus.fpl?locus=BIO5), [BIO4/YNR057C](http://www.yeastgenome.org/cgi-bin/locus.fpl?locus=BIO4), [BIO3/YNR058W](http://www.yeastgenome.org/cgi-bin/locus.fpl?locus=BIO3), [POS5/YPL188W](http://www.yeastgenome.org/cgi-bin/locus.fpl?locus=POS5) |
| [*ornithine metabolic process*](http://www.yeastgenome.org/cgi-bin/GO/goTerm.pl?goid=6591) | 5 out of 302 genes, 1.7% | 7 out of 7167 genes, 0.1% | 0.00170 | [ARG5,6/YER069W](http://www.yeastgenome.org/cgi-bin/locus.fpl?locus=ARG5), [ARG2/YJL071W](http://www.yeastgenome.org/cgi-bin/locus.fpl?locus=ARG2), [ARG3/YJL088W](http://www.yeastgenome.org/cgi-bin/locus.fpl?locus=ARG3), [ARG7/YMR062C](http://www.yeastgenome.org/cgi-bin/locus.fpl?locus=ARG7), [ARG8/YOL140W](http://www.yeastgenome.org/cgi-bin/locus.fpl?locus=ARG8) |
| [*branched chain family amino acid metabolic process*](http://www.yeastgenome.org/cgi-bin/GO/goTerm.pl?goid=9081) | 7 out of 302 genes, 2.3% | 17 out of 7167 genes, 0.2% | 0.00201 | [LEU1/YGL009C](http://www.yeastgenome.org/cgi-bin/locus.fpl?locus=LEU1), [BAT1/YHR208W](http://www.yeastgenome.org/cgi-bin/locus.fpl?locus=BAT1), [ILV3/YJR016C](http://www.yeastgenome.org/cgi-bin/locus.fpl?locus=ILV3), [ILV5/YLR355C](http://www.yeastgenome.org/cgi-bin/locus.fpl?locus=ILV5), [ILV2/YMR108W](http://www.yeastgenome.org/cgi-bin/locus.fpl?locus=ILV2), [LEU4/YNL104C](http://www.yeastgenome.org/cgi-bin/locus.fpl?locus=LEU4), [LEU9/YOR108W](http://www.yeastgenome.org/cgi-bin/locus.fpl?locus=LEU9) |
| [*ornithine biosynthetic process*](http://www.yeastgenome.org/cgi-bin/GO/goTerm.pl?goid=6592) | 4 out of 302 genes, 1.3% | 4 out of 7167 genes, 0.1% | 0.00209 | [ARG5,6/YER069W](http://www.yeastgenome.org/cgi-bin/locus.fpl?locus=ARG5), [ARG2/YJL071W](http://www.yeastgenome.org/cgi-bin/locus.fpl?locus=ARG2), [ARG7/YMR062C](http://www.yeastgenome.org/cgi-bin/locus.fpl?locus=ARG7), [ARG8/YOL140W](http://www.yeastgenome.org/cgi-bin/locus.fpl?locus=ARG8) |
| [*cellular response to chemical stimulus*](http://www.yeastgenome.org/cgi-bin/GO/goTerm.pl?goid=70887) | 24 out of 302 genes, 7.9% | 214 out of 7167 genes, 3.0% | 0.00671 | [UGA2/YBR006W](http://www.yeastgenome.org/cgi-bin/locus.fpl?locus=UGA2), [FUS1/YCL027W](http://www.yeastgenome.org/cgi-bin/locus.fpl?locus=FUS1), [AFR1/YDR085C](http://www.yeastgenome.org/cgi-bin/locus.fpl?locus=AFR1), [STE5/YDR103W](http://www.yeastgenome.org/cgi-bin/locus.fpl?locus=STE5), [GLN3/YER040W](http://www.yeastgenome.org/cgi-bin/locus.fpl?locus=GLN3), [DSE1/YER124C](http://www.yeastgenome.org/cgi-bin/locus.fpl?locus=DSE1), [HSP12/YFL014W](http://www.yeastgenome.org/cgi-bin/locus.fpl?locus=HSP12), [GAT1/YFL021W](http://www.yeastgenome.org/cgi-bin/locus.fpl?locus=GAT1), [STE2/YFL026W](http://www.yeastgenome.org/cgi-bin/locus.fpl?locus=STE2), [AGA2/YGL032C](http://www.yeastgenome.org/cgi-bin/locus.fpl?locus=AGA2), [CTT1/YGR088W](http://www.yeastgenome.org/cgi-bin/locus.fpl?locus=CTT1), [YHI9/YHR029C](http://www.yeastgenome.org/cgi-bin/locus.fpl?locus=YHI9), [CUP1-1/YHR053C](http://www.yeastgenome.org/cgi-bin/locus.fpl?locus=CUP1), [CUP1-2/YHR055C](http://www.yeastgenome.org/cgi-bin/locus.fpl?locus=CUP1), [YJL144W](http://www.yeastgenome.org/cgi-bin/locus.fpl?locus=YJL144W), [MCR1/YKL150W](http://www.yeastgenome.org/cgi-bin/locus.fpl?locus=MCR1), [MID2/YLR332W](http://www.yeastgenome.org/cgi-bin/locus.fpl?locus=MID2), [SST2/YLR452C](http://www.yeastgenome.org/cgi-bin/locus.fpl?locus=SST2), [GAD1/YMR250W](http://www.yeastgenome.org/cgi-bin/locus.fpl?locus=GAD1), [NCE103/YNL036W](http://www.yeastgenome.org/cgi-bin/locus.fpl?locus=NCE103), [MFA2/YNL145W](http://www.yeastgenome.org/cgi-bin/locus.fpl?locus=MFA2), [AGA1/YNR044W](http://www.yeastgenome.org/cgi-bin/locus.fpl?locus=AGA1), [GCY1/YOR120W](http://www.yeastgenome.org/cgi-bin/locus.fpl?locus=GCY1), [POS5/YPL188W](http://www.yeastgenome.org/cgi-bin/locus.fpl?locus=POS5) |
