# Supplementary material for: A Plant Virus Movement Protein Regulates the Gcn2p Kinase in Budding Yeast
Source: PLoS One. 2011 Nov 8;6(11):e27409. doi: 10.1371/journal.pone.0027409 (PMC3210792; doi:10.1371/journal.pone.0027409)
Supplement: Table S4 — Percentage of unbudded cells in yeasts transformed with the empty vector (pCM) and MPpnrsv (MP) expressing plasmids. (DOC) [file pone.0027409.s005.doc]

**TABLE S4.** Percentage of unbudded cells in yeasts transformed with the empty vector (pCM) and MPpnrsv expressing plasmid.

| Time(h) | pCM | | MPpnrsv | |
| --- | --- | --- | --- | --- |
|  | +DOX* | -DOX | +DOX | -DOX |
| 0 | 36+4 | 37+2 | 28+2 | 36+1 |
| 3 | 40+4 | 40+4 | 47+4 | 53+2 |

Yeast cultures were grown for 3 hours in SD medium with or without DOX. Unbudded (%) cells were scored by microscopic observation. Data represent the average +/- standard error (s.e.) of two independent experiments each one done in triplicate.

*DOX: Doxicycline
