# Supplementary material for: A Plant Virus Movement Protein Regulates the Gcn2p Kinase in Budding Yeast
Source: PLoS One. 2011 Nov 8;6(11):e27409. doi: 10.1371/journal.pone.0027409 (PMC3210792; doi:10.1371/journal.pone.0027409)
Supplement: Table S5 — Primers used in this study. Restriction sites Pme I and Pst I are underlined. (DOC) [file pone.0027409.s006.doc]

**TABLE S5.** Primers used in this study. Restriction sites *Pme* I *and Pst* I are underlined

| primer | sequence | Viral protein amplified |
| --- | --- | --- |
| VP765 | GTTTAAACATGGCCGGTGTC AGTAAAAC | PNRSV MP and MPpΔHR |
| VP766 | CTGCAGAGCACTTCTAAAACTACCGAC | PNRSV MP and MPpΔHR |
| VP1278 | GTTTAAACATGTCTAACATAGTTTCTC | BMV MP |
| VP1279 | CTGCAGTTTAATTCTAAGCGTAGG | BMV MP |
| VP1284 | GTTTAAACATGGCTTTCCAAGGTAC | CMV MP |
| VP1285 | CTGCAGAAGACCGTTAACCACCTG | CMV MP |
| VP1280 | GTTTAAACATGGCTCTAGTTGTTAAAG | TMV MP |
| VP1281 | CTGCAGAAACGAATCCGATTCGGC | TMV MP |
| VP1286 | GTTTAAACATGGATGGGAAGACTACC | GFLV MP |
| VP1287 | CTGCAGTCTCACGGTTGAGCTCAG | GFLV MP |
