# Supplementary material for: A Plant Virus Movement Protein Regulates the Gcn2p Kinase in Budding Yeast
Source: PLoS One. 2011 Nov 8;6(11):e27409. doi: 10.1371/journal.pone.0027409 (PMC3210792; doi:10.1371/journal.pone.0027409)
Supplement: Table S6 — Yeast Strains used in this study. (DOC) [file pone.0027409.s007.doc]

**TABLE S6.** Yeast Strains used in this study.

| Strain name | Relevant genotype | Reference |
| --- | --- | --- |
| BY4741 | MATα leu2Δ0, his3Δ0, met15Δ0, ura3Δ0 | EUROSCAF |
| *gcn2Δ* | BY4741, gcn2::KANMX4 | EUROSCAF |
| *gcn4Δ* | BY4741, gcn4::KANMX4 | EUROSCAF |
| pcm | BY4741, pcm262 | this study |
| MPpnrsv | BY4741, pcm262:MPpnrsv | this study |
| MPpnrsvΔHR | BY4741, pcm262:MPpnrsvΔHR | this study |
| MP BMV | BY4741, pcm262:MP BMV | this study |
| MP CMV | BY4741, pcm262:MP CMV | this study |
| MP TMV | BY4741, pcm262:MP TMV | this study |
| MP GFLV | BY4741, pcm262:MP GFLV | this study |
